# Restoration of catalytic activity by the preservation of ligand structure: Cu-catalysed asymmetric conjugate addition with 1,1-diborylmethane†

**DOI:** 10.1039/d0sc06543a

**Published:** 2021-01-21

**Authors:** Changhee Kim, Byeongdo Roh, Hong Geun Lee

**Affiliations:** Department of Chemistry, Seoul National University Seoul 08826 Republic of Korea hgleee@snu.ac.kr

## Abstract

Reported herein is a novel reaction engineering protocol to enhance the efficiency of a transition metal-catalysed process by strategically preventing ligand degradation. Based on spectroscopic investigations, a decomposition pathway of a chiral phosphoramidite ligand during a Cu-catalysed reaction was identified. The involvement of the destructive process could be minimized under the modified reaction conditions that control the amount of nucleophilic alkoxide base, which is the origin of ligand decomposition. Overall, the strategy has been successfully applied to a new class of asymmetric conjugate addition reactions with bis[(pinacolato)boryl]methane, in which α,β-unsaturated enones are utilised as substrates.

## Abstract 2

A novel Cu-catalysed asymmetric conjugate addition reaction with bis[(pinacolato)boryl]methane using α,β-unsaturated enones as substrates has been developed on the basis of strategic preservation of the supporting ligand.

## Graphical abstract

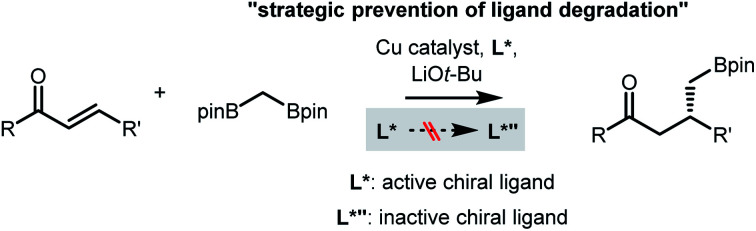

## Introduction

**Scheme 1:**
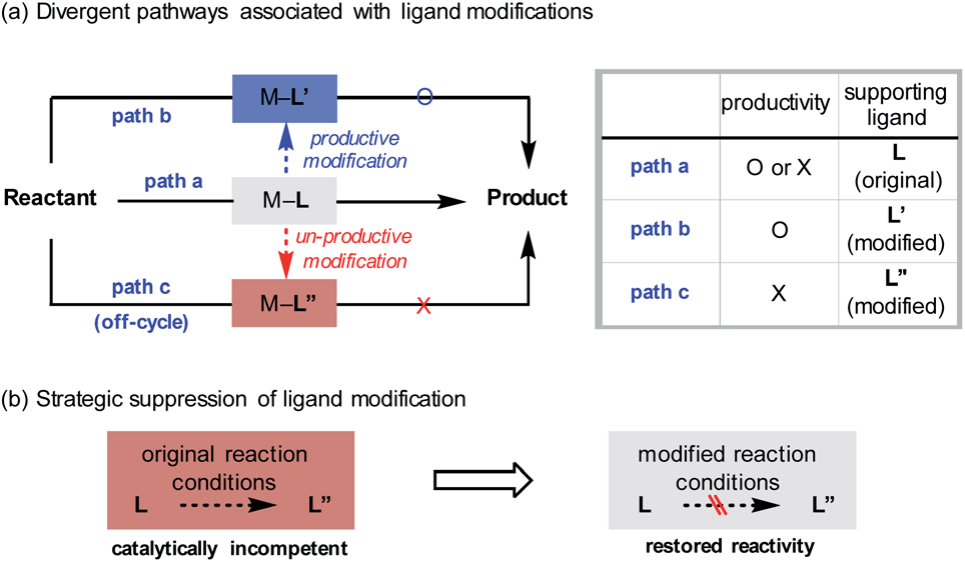
Ligand modifications in transition metal-catalysed reactions.

The introduction of novel supporting ligands for transition metals has contributed significantly to the development of homogeneous catalysis based on transition metals. The ligands not only ensure the stability of the metal centre, but also contribute to the activity of the system by performing various catalytically relevant functions, such as stereochemical induction or modulation of electronic properties.^1^ While it is generally accepted that the ligands are structurally invariant species over the course of their catalytic function (Scheme 1a, path a), processes that involve changes in their architecture can take place under the given reaction conditions. For instance, constructive *in situ* ligand modifications have been reported in the context of cross-coupling^2^ or addition reactions of organometallic species (Scheme 1a, path b).^3^ In such cases, the spontaneous conversion of the ligand (**L** to **L′**) eventually facilitated the desired reactivity of the catalytic system. If, on the other hand, the ligand originally administered had been converted into an inactive state by structural modifications (**L** to **L′′**), the catalytic system would lose its ability to mediate the targeted reaction (Scheme 1a, path c). In these circumstances, it can be difficult to assess the catalyst performance accurately, as either the inherent incompetence of the system or the destruction of the given ligand structure can be the cause of the substandard reactivity. From another point of view, however, this would offer an opportunity for redeeming the reactivity of an unsuccessful system if the origin of malfunction is the latter (Scheme 1b). By strategically preventing the undesirable modification process using suitable reaction conditions, the maximum capacity of the system can be recovered. This could avoid the costly incorporation of a large excess of valuable ligands or tedious re-evaluation of a new ligand system, the success of which is not guaranteed.

**Scheme 2:**
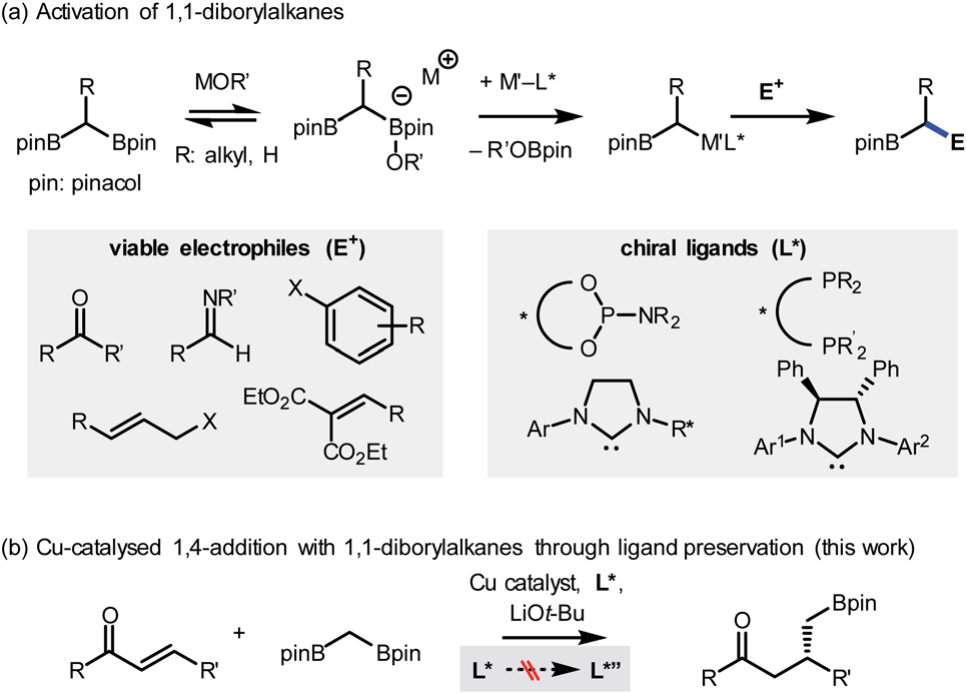
Stereoselective reactions with 1,1-diborylalkanes.

Here, the possibility of restoring the reactivity of a catalytic system was identified from a transition metal-catalysed stereoselective addition reaction employing 1,1-diborylalkanes (Scheme 2a).^4^ The synthetically important process was initiated by the activation of a C–B bond through the action of oxygen-based nucleophiles. Subsequent transmetalation to the transition metal catalyst, such as Pd, Cu, or Ir, generates an organometallic species that can react with a variety of electrophilic counterparts to form stereochemically enriched alkylboronate products, a class of compounds that can be diversely functionalized.^5^ Over the course of the reaction, the involvement of chiral ligands, including bidentate phosphines,^5*a*^ N-heterocyclic carbenes,^5*b*,*c*^ and most commonly phosphoramidites,^5*d*–*h*^ plays a critical role in terms of stereochemical induction. In view of the documented susceptibility of phosphoramidite ligands to hydroxide or alkoxide nucleophiles,^6^ it has been recognized that an undesirable ligand degradation pathway may be involved in reactions with phosphoramidite supporting ligands, especially if the reaction outcome is suboptimal. Accordingly, we evaluated the feasibility of using the ligand preservation approach to promote the insufficient reactivities of reactions with 1,1-diborylalkanes that are based on transition metal-phosphoramidite complexes (Scheme 2b). As a model reaction for investigation, the underdeveloped Cu-catalysed conjugate addition with 1,1-diborylmethane to an α,β-unsaturated ketone substrate was selected.^7^

## Results and discussion

An initial evaluation of the catalyst system with a range of chiral ligands was performed using *trans*-chalcone as a substrate (Table 1). The stereoselective conjugate addition reaction with bis[(pinacolato)boryl]methane pronucleophile was carried out in the presence of a Cu(i) source, a chiral supporting ligand, and LiO*t*-Bu, which induces C–B bond cleavage. Ligands with a phosphoramidite backbone exhibited the most pronounced reactivity with concomitant formation of the boron-Wittig product, which accounts for the remainder of the reactivity (entries 2–6).^8^ Among them, (*S*)-MonoPhos (**L1**) demonstrated the best performance in terms of product formation and stereoinduction, although the result was not synthetically ideal (entry 2). Catalyst systems based on other chiral ligands suffered from low levels of conversion (entries 7–9) and/or competitive boron-Wittig reactions. The use of a large excess of ligand, a commonly utilised technique that aids in facilitated ligand ligation, could not promote the reactivity to a practically useful level (entry 3). When attempting to identify conditions with superior reactivity, we found that incorporating Li(acac) into the reaction mixture significantly improved both product yield and enantiomeric excess (entry 10), and additional studies with variations in the catalyst loading showed that 10 mol% catalyst loading was required for the best performance (entries 11 and 12).^9^ However, the use of other lithium salts, acetylacetonate derivatives, or Lewis acidic entities was not as effective (entries 13–21).^10^ Eventually, the (*S*)-MonoPhos-based catalytic system with the Li(acac) additive was chosen as the optimal system.

**Table 1:** Optimization of reaction conditions

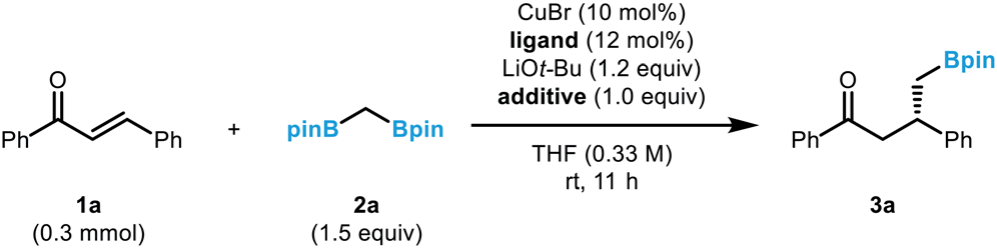					
Entry^a^	Ligand	Additive	Conv. (%)	Yield^b^ (%)	ee^c^ (%)
1	—	—	>99	20	—
2	**L1**	—	>99	63	76
3	**L1** d	—	>99	68	79
4	**L2**	—	>99	40	55
5	**L3**	—	74	19	6
6	**L4**	—	98	40	4
7	**L5**	—	<5	Trace	—
8	**L6**	—	28	Trace	—
9	**L7**	—	29	Trace	—
10	**L1**	Li(acac)	>99	70	92
11^e^	**L1**	Li(acac)	>99	62	86
12^f^	**L1**	Li(acac)	>99	35	83
13	**L1**	LiF	81	76	77
14	**L1**	LiCl	30	29	<5
15	**L1**	LiBr	28	28	<5
16	**L1**	LiClO_4_	<5	Trace	—
17	**L1**	Li(TMHD)	44	35	<5
18	**L1**	Na(hfacH)	32	13	70
19	**L1**	TMSCl	36	24	7
20	**L1**	BF_3_·OEt_2_	<5	Trace	—
21	**L1**	ZnBr_2_	<5	Trace	—
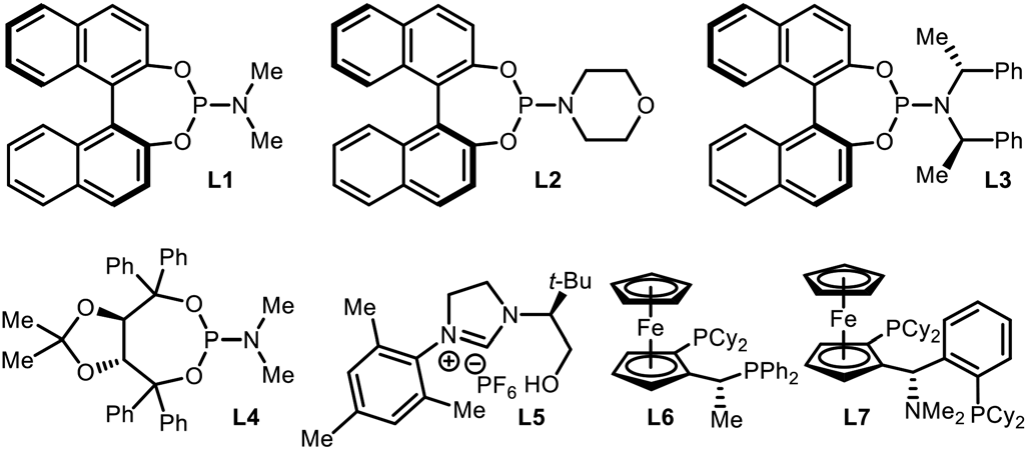					

a Reaction conditions: CuBr (10 mol%), ligand (12 mol%), LiO*t*-Bu (1.2 equiv.), additive (1.0 equiv.), **2a** (1.5 equiv.), and **1a** (0.3 mmol, 0.33 M) in THF (0.9 mL).

b Determined by GC analysis with *n*-dodecane as an internal standard.

c Determined by HPLC analysis.

d 24 mol% of **L1** was used.

e 5 mol% of CuBr and 6 mol% of **L1** were used.

f 2.5 mol% of CuBr and 3 mol% of **L1** were used. acac = acetylacetone, TMHD = 2,2,6,6-tetramethyl-3,5-heptanedione, hfacH = hexafluoroacetylacetone.

**Fig. 1:**
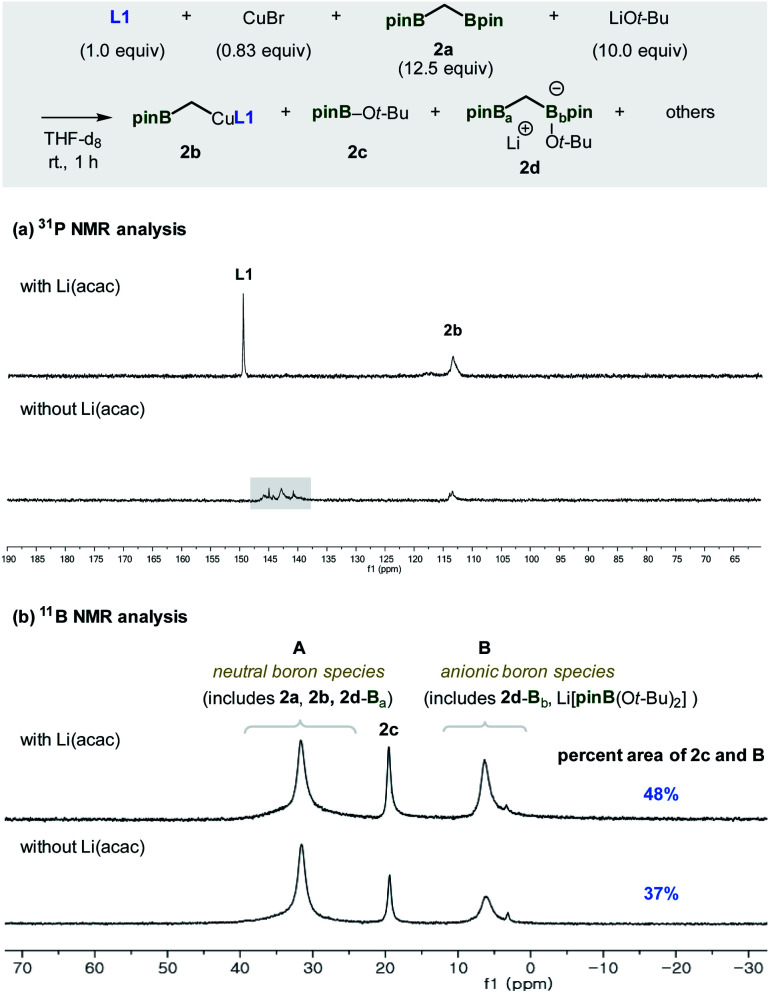
Spectroscopic analysis of the catalytic system. Reaction conditions: **L1** (1.0 equiv., 0.012 mmol), CuBr (0.83 equiv.), **2a** (12.5 equiv.), LiO*t*-Bu (10 equiv.), and Li(acac) (8.3 equiv.) in THF-d_8_ (0.5 mL).

To better understand the factors that contribute to the successful catalytic system containing Li(acac), catalytically relevant species were prepared and examined using ^31^P and ^11^B NMR experiments (Fig. 1). When all the reaction components, including Li(acac), were introduced in the absence of the enone substrate, the active nucleophilic species (**2b**) was formed. Based on the observed reactivity towards the enone substrates and ^31^P NMR analysis, the structure of the reactive species was assigned to be the ligand-bound alkyl copper complex (Fig. 1a).^11,12^ Besides **2b**, the uncoordinated ligand **L1** was the only significant species that was detected. On the other hand, in the case of an identical mixture that did not contain Li(acac), no free ligand was observed. More importantly, the relative mass balance of **2b** diminished significantly. Instead, unidentified chemical species were found (*δ* 137–147 ppm, box), indicating decomposition of catalytically responsible species based on **L1** (*vide infra*).^13^

The origin of the observed catalyst decomposition could be deduced using ^11^B NMR studies (Fig. 1b). The ^11^B NMR spectra of the reaction mixture consists of three major signals originating from C-ligated neutral Bpin species, including **2a**, **2b**, and **2d**-B_a_ (**A**), heteroatom-bound neutral Bpin species (**2c**), and anionic complexes, such as **2d**-B_b_ or Li[pinB(O*t*-Bu)_2_] (**B**).^14^ Complete quantitative analysis of all relevant species was difficult to perform because of the complexity of the system. It is evident, however, that the application of Li(acac) drove the equilibrium in the direction of increasing the amount of **2c** and **B**. Since the formation of **2c** and **B** stems from the coordination of the alkoxide at the boron centre, the result indicates an escalated level of the entrapped alkoxide nucleophile in the reaction mixture. Eventually, a reduced amount of alkoxide is available for ligand degradation to enhance the catalytic activity of the system.

**Fig. 2:**
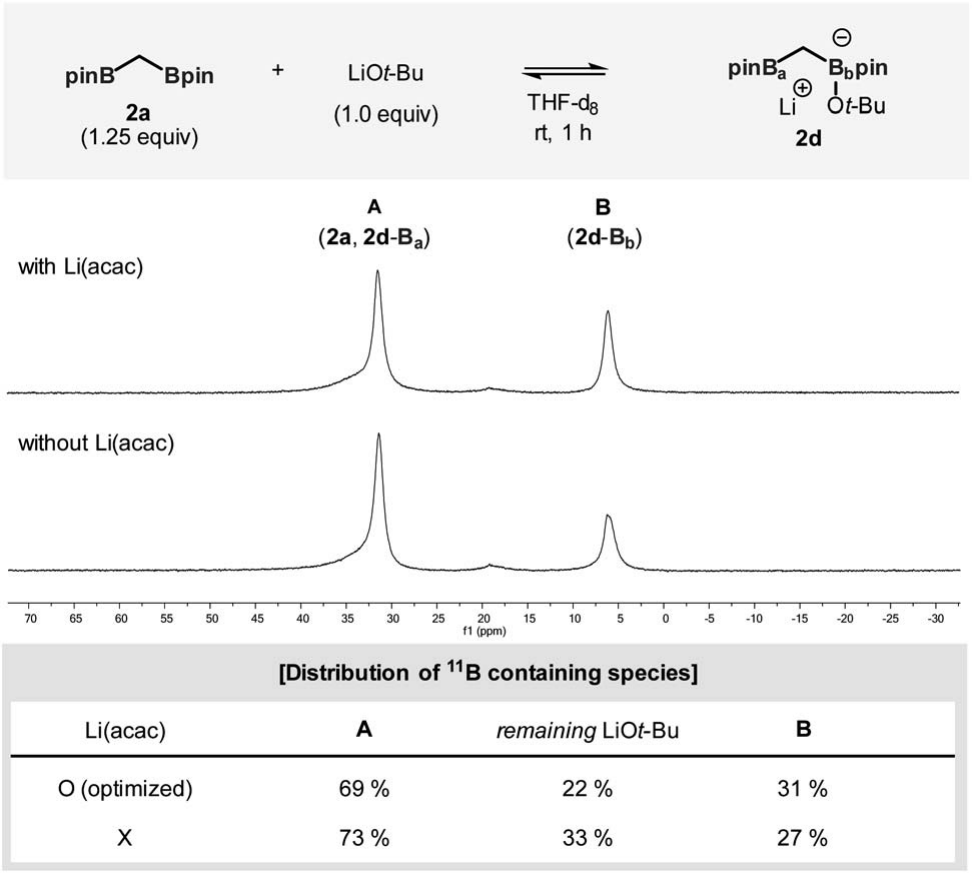
Equilibrium of bis[(pinacolato)boryl]methane with LiO*t*-Bu monitored by ^11^B NMR. Reaction conditions: **2a** (1.25 equiv., 0.15 mmol), LiO*t*-Bu (1.0 equiv.), and Li(acac) (0.83 equiv.) in THF-d_8_ (0.5 mL).

The impact of added Li(acac) was further investigated in a more controlled experiment in which **2a** was independently exposed to the LiO*t*-Bu nucleophile (Fig. 2). The addition of Li(acac) shifted the equilibrium to the direction of the ate-complex formation (**2d**). During the reaction, a significant portion of the initially administered LiO*t*-Bu nucleophiles was consumed: in the case of the Li(acac)-free conditions the effective concentration of LiO*t*-Bu is 50% higher than that of the conditions with Li(acac).^15^

The complete decomposition pathway of the phosphoramidite ligand remains elusive. However, preliminary mass spectroscopic analysis of the reaction mixture suggests the involvement of P–O or P–N bond cleavage in the backbone structure of **L1** in the presence of LiO*t*-Bu.^16^ It is believed that the initial nucleophilic attack of LiO*t*-Bu generates an intermediate that leads to other decomposition products.

**Fig. 3:**
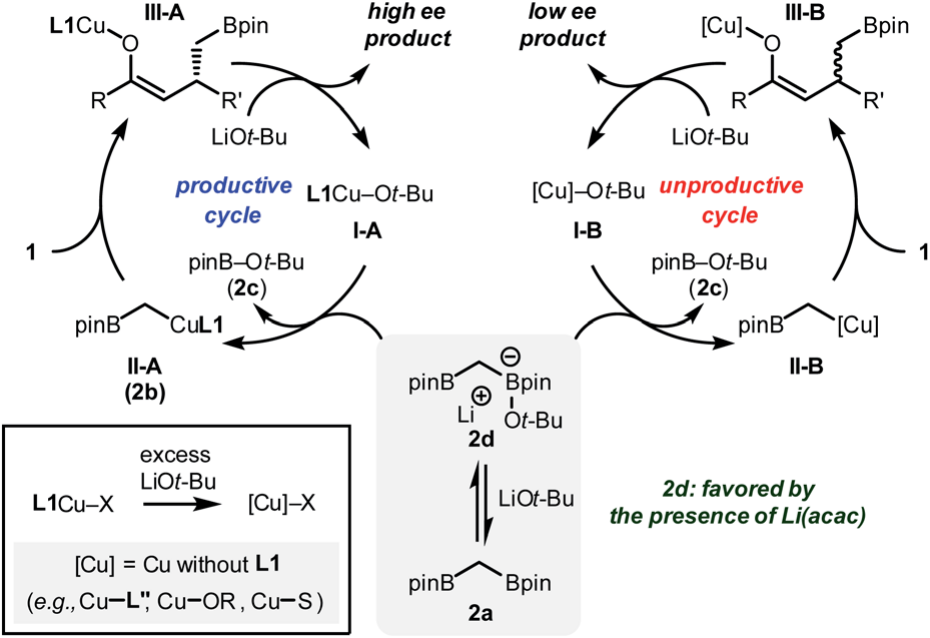
Plausible mechanistic scenario.

Based on these observations, a mechanistic scenario that consists of two independent catalytic cycles is presented (Fig. 3). In the productive cycle, the active catalyst **I-A**, which is generated by the combination of **L1**, CuBr, and LiO*t*-Bu, undergoes transmetalation with **2a** to generate a copper alkyl species **II-A** (Fig. 1, **2b**). The reactive carbon-based nucleophile should undergo an enantioselective conjugate addition reaction with enone substrate **1** to provide a copper enolate intermediate **III-A**, an immediate precursor of the desired product with high stereochemical integrity. During the operation of the major catalytic cycle, **2a** should be in equilibrium with its ate-complex form **2d**, a species responsible for the rate-determining transmetalation, by the involvement of LiO*t*-Bu.^17^ Ultimately, the equilibrium controls the amount of the alkoxide base in the reaction mixture that is available to destroy active catalytic species. The process includes the irreversible modification of the phosphoramidite ligand **L1**, which contributes to furnishing catalytically incompetent species ([Cu]). These species include Cu-complexes ligated with the decomposed ligand (**L′′**), solvent (S), and/or other heteroatom-containing species present in the reaction mixture. Consequently, an alternative catalytic cycle with lower stereoselectivity operates with the intermediates **II-B** and **III-B**. As determined from the NMR experiments, the added Li(acac) controls the extent of the unproductive catalytic cycle by controlling the equilibrium of **2a**, LiO*t*-Bu, and **2d**.^18^

With the optimized conditions in hand, we evaluated the scope of the developed reaction using various α,β-unsaturated enone substrates (Table 2). Reactions with chalcone derivatives bearing electron-donating or electron-withdrawing substituents on the arene ring at the β position of the enones afforded the desired boronic esters with good yield and enantioselectivity (**3a–3g**), although the placement of an *ortho* substituent of the arene resulted in substantially diminished yield (**3c**). It is noteworthy that the efficiency of the reaction could be successfully conserved in a larger-scale reaction, which demonstrates the robustness of the reaction (**3a**). Variations on the phenyl ring directly attached to the carbonyl group were also tolerated (**3h–3j**). Importantly, the presence of halogen substituents, which can be used as a handle for further functionalization through cross-coupling reactions, did not affect the outcome of the conjugate addition reaction (**3f**, **3g**, **3i**, **3j**). Moreover, biologically important heterocycles such as furan or thiofuran could be successfully incorporated into the substrate structure (**3k–3n**). Additionally, the reactivity could be extended to alkyl-substituted α,β-unsaturated enones. When a diverse array of alkyl groups was introduced at the β-position of the enone substrate, the optimized conditions operated smoothly (**3o–3r**). Meanwhile, an alkyl group at the carbonyl carbon significantly decreased the efficiency of the product formation due to competitive 1,2-addition reactions. Only the substrate containing a bulky *t*-butyl group was a viable substrate for the conjugate addition (**3s**). Finally, an enone with an extended π-system afforded the desired 1,4-addition products (**3t**). A side product originating from the competing 1,6-addition was not observed.

**Table 2:** Enantioselective conjugate addition with 1,1-diborylmethane to α,β-unsaturated enones^a^

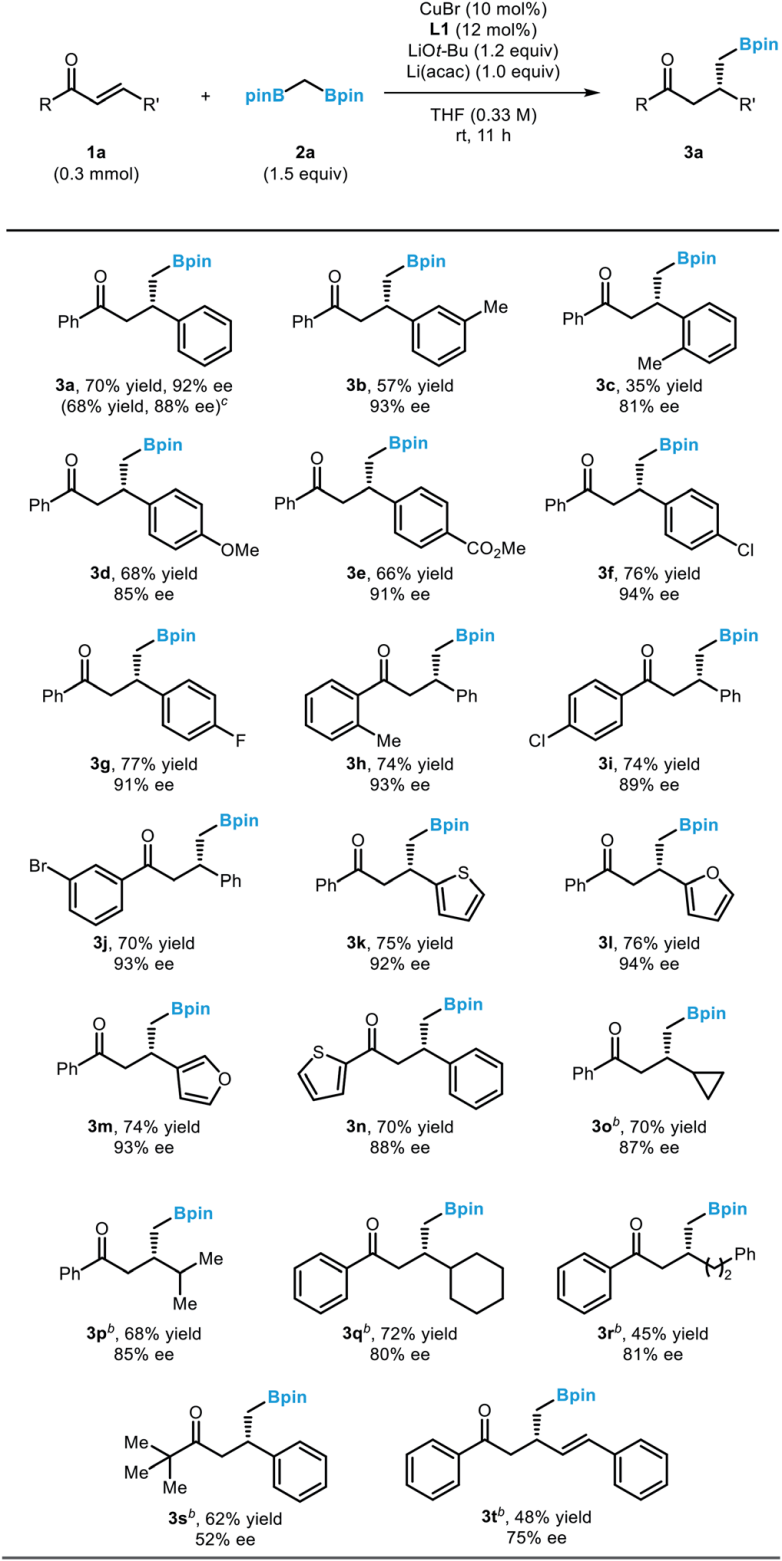

a Reaction conditions: **1a** (0.3 mmol), **2a** (1.5 equiv.), CuBr (10 mol%), **L1** (12 mol%), LiO*t*-Bu (1.2 equiv.), and Li(acac) (1.0 equiv.) in THF (0.9 mL), rt, 11 h, under nitrogen.

b The reaction was conducted for 36 h.

c The reaction was carried out on a 3 mmol scale.

**Scheme 3:**
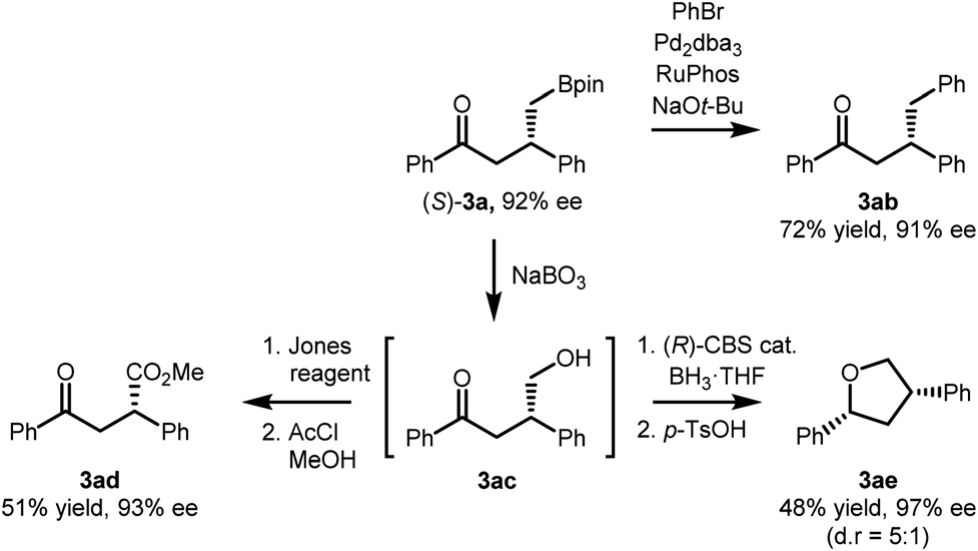
Transformation of chiral (*S*)-**3a**.

The synthetic utility of the developed reaction was further demonstrated based on the versatility of the stereochemically enriched γ-ketoboronic ester product (Scheme 3). The Suzuki–Miyaura cross coupling reaction of (*S*)-**3a** and bromobenzene afforded the corresponding γ-phenyl ketone product in 72% yield (**3ab**).^19^ During the C–C bond-forming reaction, the stereochemical integrity of the stereogenic centre remained unaffected. Moreover, the C–B bond of (*S*)-**3a** could be readily oxidized in the presence of NaBO_3_·4H_2_O to provide a γ-hydroxy ketone (**3ac**), which in turn was functionalized into more complex products. A two-step sequence involving Jones oxidation and Fisher esterification furnished the corresponding methyl ester in 51% overall yield (**3ad**). Alternatively, an enantioselective reduction of the carbonyl group in the presence of the (*R*)-oxazaborolidine catalyst,^20^ followed by stereospecific benzylic displacement^21^ afforded marine natural product calyxolane B in its enantiomeric form (**3ae**).^22^

## Conclusions

In conclusion, a novel reaction engineering strategy has been developed to allow for full utilisation of the catalytic activity of a transition-metal-catalysed reaction. By identifying the ligand decomposition pathway and using an additive-based restoration method, the efficiency of an underdeveloped reaction could be brought to synthetically useful levels. The method has been successfully applied to an enantioselective conjugate addition reaction with 1,1-bis[(pinacolato)boryl]methane catalysed by a Cu-phosphoramidite catalyst system. We believe that the strategy should have a broader impact on reactions catalysed by related metal–ligand complexes. It is anticipated that analogous reaction engineering systems will ultimately be implemented for transition metal catalysis in general.

## Conflicts of interest

There are no conflicts to declare.

## Supplementary Material

SC-012-D0SC06543A-s001
