# Supplementary material for: Restoration of catalytic activity by the preservation of ligand structure: Cu-catalysed asymmetric conjugate addition with 1,1-diborylmethane
Source: Chem Sci. 2021 Jan 21;12(10):3668–73. doi: 10.1039/d0sc06543a (PMC8179450; doi:10.1039/d0sc06543a)

Electronic Supplementary Material (ESI) for Chemical Science.
This journal is © The Royal Society of Chemistry 2021

Restoration of Catalytic Activity by the Preservation of Ligand Structure: Cu-catalysed Asymmetric Conjugate Addition with 1,1-Diborylmethane

*Changhee Kim, Byeongdo Roh, and Hong Geun Lee**

Department of Chemistry, Seoul National University, Seoul 08826, Republic of Korea

E-mail: hgleee@snu.ac.kr

SUPPORTING INFORMATION

Table of Contents

1. General experimental details S3

2. General procedure for preparation of starting materials S4

3. Evaluation on reaction conditions S5

4. General procedure for enantioselective conjugate addition of bis[(pinacolato)boryl]methane to α,β-unsaturated enones S13

5. General procedure for further derivatizations of (*S*)-**3a** S53

6. General procedure for ^{31}P, ^{11}B NMR experiments S59

7. Preliminary mass spectroscopic data (HRMS) S70

8. References S75

9. ^{1}H and ^{13}C NMR spectra S76

1. General experimental details

Commercially available reagents were purchased from Sigma Aldrich, TCI, Alfa Aesar, Acros, Fluka and Strem, and used without further purification, unless otherwise noted. 1,1-bis[(pinacolato)boryl]methane and (*S*)-MonoPhos were purchased from Angene and Chemscene, respectively. Dry tetrahydrofuran (THF) was obtained by passing through an activated alumina column of solvent purification system, and further degassed by repeated sonication under house vacuum and vigorous purging with nitrogen gas. Yields represent chromatographically homogeneous product. All reactions were monitored by thin-layers chromatography (TLC) with 0.25 mm E. Merck silica gel plates (60F-254) using UV light or staining solution (potassium permangnate and *p*-anisaldehyde) for visualization. NMR spectra were recorded on an Agilent 400-MR DD2 Magnetic Resonance System, JNM-ECX 400 Spectrometer (JEOL) or/and Varian/Oxford As-500 instrument and calibrated using residual undeuterated solvent ($CHCl_3$ at δ7.26 ppm for ^1H NMR and δ 77.16 ppm for ^{13}C NMR) as internal reference. $BF_3{\cdot}OEt_2$ (15% w/w in $CDCl_3$) and H_3PO_4 (85% w/w in H_2O) solution were used as external standards for obtaining ^{11}B and ^{31}P NMR, respectively. For ^{19}F NMR spectra, fluorobenzene was used as an external standard (–113.15 ppm). Chemical shifts (δ) are reported in parts per million (ppm). Coupling constants (*J*) are reported in hertz (Hz). For NMR spectra, multiplicities are given as: s = singlet, d = doublet, t = triplet, q = quartet, m = multiplet, br = broad. Enantiomeric excess (ee) were determined by employing High-Performance Liquid Chromatography (HPLC) with columns containing chiral stationary phase and HPLC-grade solvent (*n*-hexane and isopropanol) as eluents. HPLC equipment was C196-E061W (Shimadzu, degassing unit : DGU-20A5R, pump : LC-20AD, auto sampler : SIL-20A, communication bus module : CBM-20A, UV/Vis detector : SPD-20A, and column oven : CTO-20A), unless otherwise noted. Optical rotations were record on JASCO P1030 polarimeter (D line of sodium vapor lamp) with a cylindrical glass cell from the same company. High resolution mass spectra (HRMS) were recorded on HRMS-ESI Q-TOF 5600 spectrometer at National Instrumentation Center for Environmental Management (NICEM) of Seoul National University.

2. General procedure for the preparation of starting materials (Scheme S1)

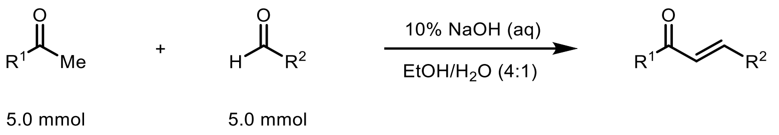

For preparation of enone substrates **2a, 2b, 2c, 2d, 2f, 2g, 2h, 2i, 2j, 2k, 2l, 2m, 2n, 2o, 2q, 2s, 2t** the following procedure was utilised.

To a 50 mL round bottom flask containing a magnetic stir bar ware added the ketone (5.0 mmol), aldehyde (5.0 mmol) and 10 ml of ethanol/H_2O mixture (v/v = 4:1). The reaction mixture was vigorously stirred for 10 min. After further stirring for 5 min at 0 °C, aqueous sodium hydroxide solution (10% w/w, 5 mL) was added dropwise. The reaction system was warmed to room temperature and the progress was monitored by TLC. Upon completion of the reaction, ethanol was evaporated under reduced pressure and the remaining aqueous phase was diluted with ethyl acetate (30 mL) and saturated aqueous solution of ammonium chloride (30 mL). The aqueous phase was separated and extracted with ethyl acetate (3 × 30 mL). The combined organic phase was dried over anhydrous sodium sulfate (Na_2SO_4), filtered, and concentrated under reduced pressure. The crude product was purified by flash column chromatography on silica gel (EtOAc/hexanes).

2e[1], **2p**[2] and **2r**[3] were prepared according to the literature procedures.

3. Evaluation on reaction conditions

Table S1. Screening of metal and base

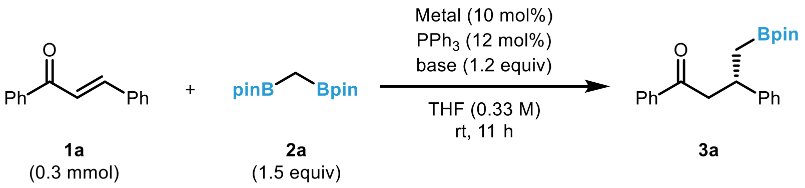

Entry[a]	Metal	Base	Conv. (%)	Yield[b] (%)	ee (%)
1	CuBr	LiO*t*-Bu	>99	45	-
2	CuI	LiO*t*-Bu	>99	27	-
3	CuTC	LiO*t*-Bu	70	32	-
4	CuOAc	LiO*t*-Bu	65	13	-
5	CuCN	LiO*t*-Bu	57	25	-
6	Cu(CF_3-acac)$_2$	LiO*t*-Bu	77	31	-
7	Cu(OAC)$_2$	LiO*t*-Bu	>99	38	-
8	$CuBr_2$	LiO*t*-Bu	>99	40	-
9	CuO	LiO*t*-Bu	87	<5	-
10	AgOTf	*n*-BuLi	56	15	-
11	AgOTf	NaO*t*-Bu	95	37	-

[a]Reaction conditions: Metal (10 mol%), PPh_3 (12 mol%), base (1.2 equiv), **2a** (1.5 equiv), and **1a** (0.3 mmol, 0.33 M) in THF (0.9 mL). [b]Determined by GC analysis with n-dodecane as an internal standard. CF3-acac = trifluoroacetylacetone.

Table S2. Screening of ligands

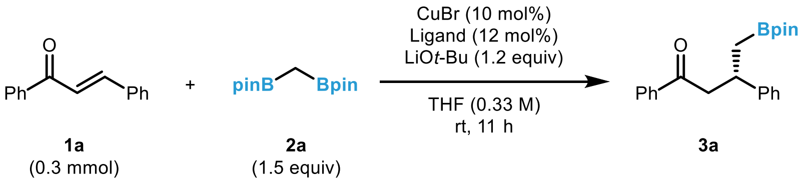

Entry[a]	Ligand	Conv. (%)	Yield[b] (%)	ee (%)
1	-	>99	20	-
2	P*n*-Bu$_3$	81	5	-
3	PPh$_3$	>99	45	-
4	XPhos	>99	25	-
5	dppb	>99	22	-
6	dppe	23	<5	-
7	dppf	61	<5	-
8	*rac*-BINAP	>99	56	-
9	BBBPY	>99	21	-
10	1,10-Phenanthroline	>99	18	-
11	(1*R*,2*R*)-DPEDA	61	20	-
12	(*S*)-MonoPhos	>99	63	-

[a]Reaction conditions: CuBr (10 mol%), ligand (12 mol%), LiO*t*-Bu (1.2 equiv), **2a** (1.5 equiv), and **1a** (0.3 mmol, 0.33 M) in THF (0.9 mL). [b]Determined by GC analysis with n-dodecane as an internal standard.

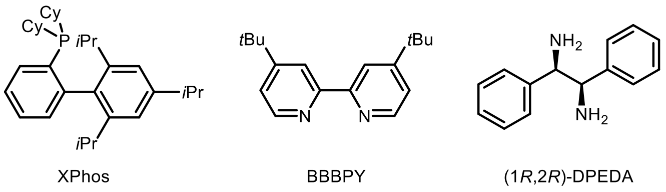

Table S3. Screening of chiral ligands

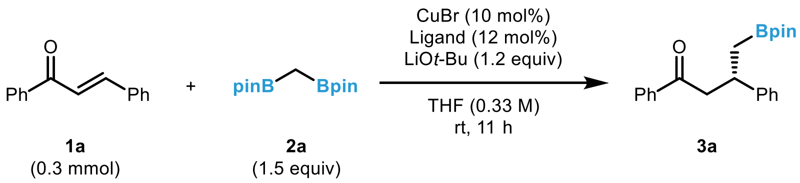

Entry[a]	Ligand	Conv. (%)	Yield[b] (%)	ee[c] (%)
1	**L1**	>99	63	76
2[d]	**L1**	>99	68	79
3	**L2**	>99	40	55
4	**L3**	74	19	6
5	**L4**	98	40	4
6	**L5**	<5	trace	-
7	**L6**	28	trace	-
8	**L7**	29	trace	-
9	**L8**	<5	trace	-
10	**L9**	<5	trace	-
11	**L10**	<5	trace	-
12	**L11**	98	34	17
13	**L12**	>99	28	–1
14	**L13**	79	41	63

[a]Reaction conditions: CuBr (10 mol%), ligand (12 mol%), LiO*t*-Bu (1.2 equiv), **2a** (1.5 equiv), and **1a** (0.3 mmol, 0.33 M) in THF (0.9 mL). [b]Determined by GC analysis with n-dodecane as an internal standard. [c]Determined by HPLC analysis. [d]**L1** of 24 mol% was used.

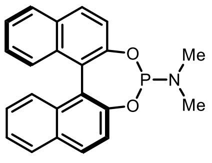

L1

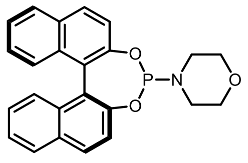

L2

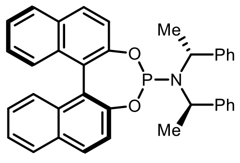

L3

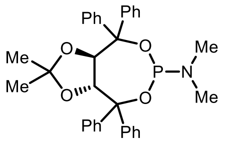

L4

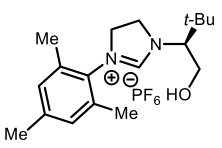

L5

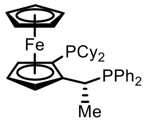

L6

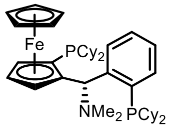

L7

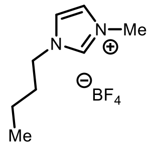

L8

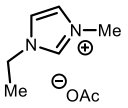

L9

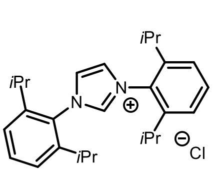

L10

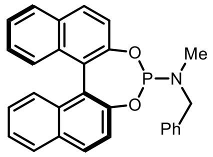

L11

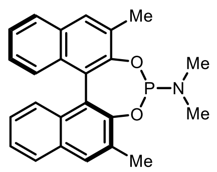

L12

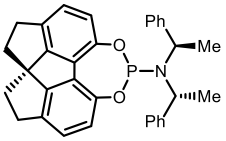

L13

Table S4. Screening of additives

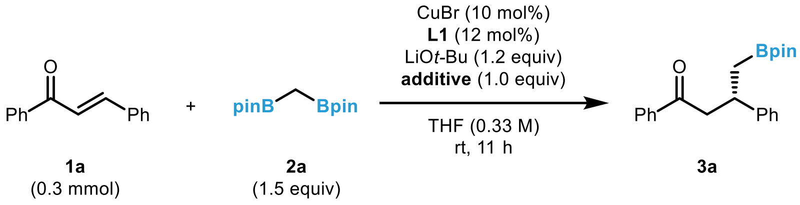

Entry[a]	Ligand	Additive	Conv. (%)	Yield[b] (%)	ee[c] (%)
1	-	-	>99	20	-
2	**L1**	-	>99	63	76
3	**L1**[d]	-	>99	68	79
4	**L1**	Li(acac)	>99	70	92
5	**L1**	LiF	81	76	77
6	**L1**	LiCl	30	29	<5
7	**L1**	LiBr	28	28	<5
8	**L1**	$LiClO_4$	<5	trace	-
9	**L1**	Li(TMHD)	44	35	<5
10	**L1**	Na(hfacH)	32	13	70
11	**L1**	TMSCl	36	24	7
12	**L1**	$BF_3{\cdot}OEt_2$	<5	trace	-
13	**L1**	$ZnBr_2$	<5	trace	-

[a]Reaction conditions: CuBr (10 mol%), **L1** (12 mol%), LiO*t*-Bu (1.2 equiv), additive (1.0 equiv), **2a** (1.5 equiv), and **1a** (0.3 mmol, 0.33 M) in THF (0.9 mL). [b]Determined by GC analysis with n-dodecane as an internal standard. [c]Determined by HPLC analysis. [d]**L1** of 24 mol% was used. acac = acetylacetone, TMHD = 2,2,6,6-tetramethyl-3,5-heptanedione, hfacH = hexafluoroacetylacetone.

Scheme S2. Versatility of the reaction

- Large scale (3.0 mmol scale)

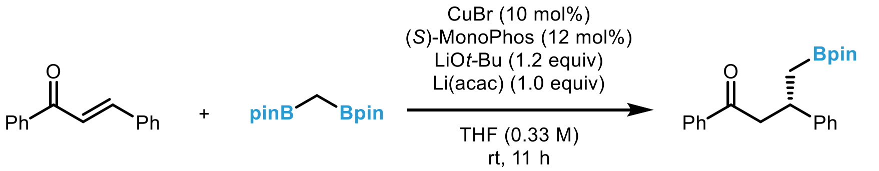

68% yield, 88% ee
(70% yield, 92% ee on 0.3 mmol)

- Lower catalyst loading (0.3 mmol scale); ***limited***

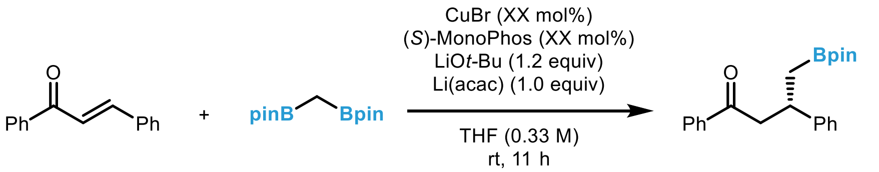

Cu/L = **2.5/3.0**, 35% yield, 83% ee
Cu/L = **5.0/6.0**, 62% yield, 86% ee
Cu/L = **10/12**, 70% yield, 92% ee

- Formation of two adjacent stereocenters (0.3 mmol scale); ***limited***

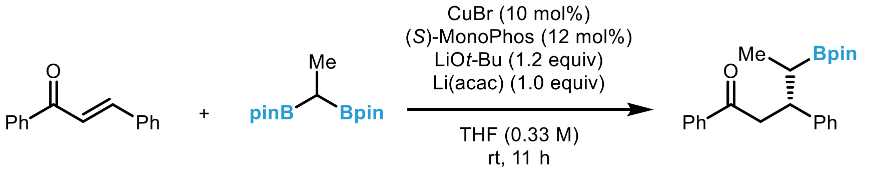

no reaction

- Sterically encumbered electrophile (0.3 mmol scale); ***limited***

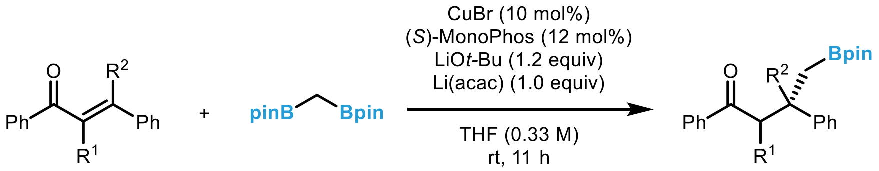

R^1= Me, R^2 = H
R^1= H, R^2 = Me
R^1= H, R^2 = Ph

no reaction

Scheme S3. Quantitative analysis of the side product.

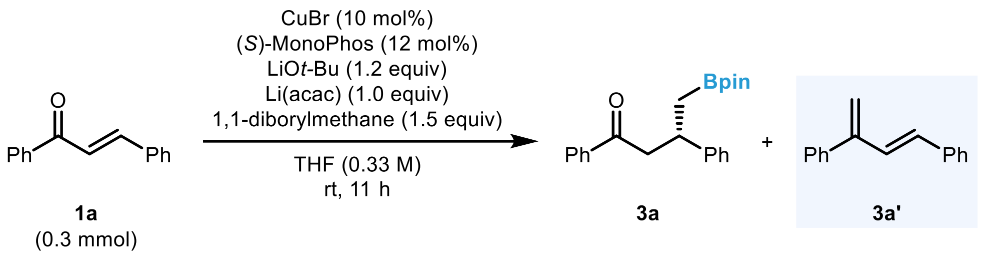

Entry[a]	Ligand	Additive	Conv. (%)	**3a**[b] (%)	**3a'**[c] (%)	ee[d] (%)
1	-	-	>99	20	60	-
2	(*S*)-MonoPhos	-	>99	63	15	76
10	(*S*)-MonoPhos	Li(acac)	>99	70	18	92

[a]Reaction conditions: CuBr (10 mol%), ligand (12 mol%), LiO*t*-Bu (1.2 equiv), Li(acac) (1.0 equiv), 1,1-diborylmethane (1.5 equiv), and **1a** (0.3 mmol, 0.33 M) in THF (0.9 mL). [b,c]Determined by NMR with 1,1,2,2-tetrachloroethane as an internal standard. [d]Determined by HPLC analysis. acac = acetylacetone.

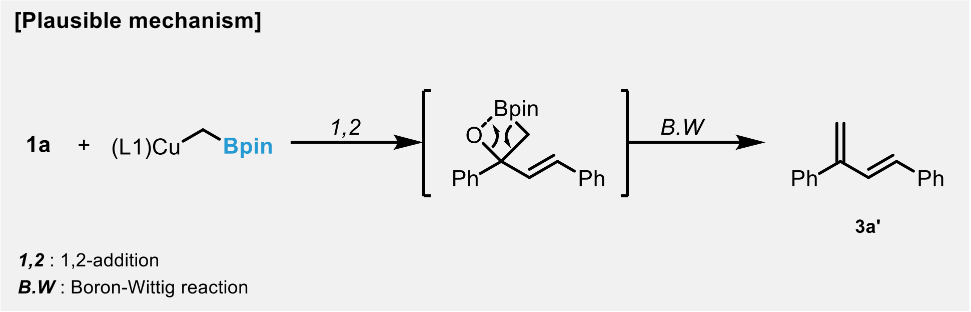

4. General procedure for enantioselective conjugate addition with 1,1-diborylmethane to enones (Table 2)

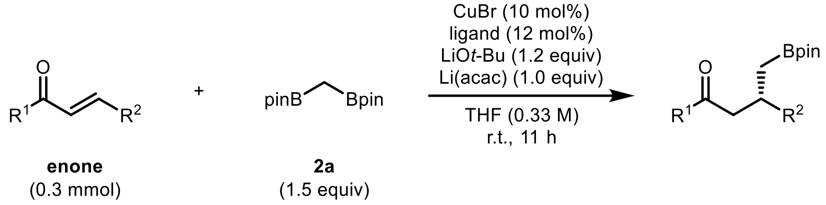

In a nitrogen-filled glove box, an oven-dried 4 mL dram-vial was charged with a magnetic stir bar, CuBr (0.030 mmol, 10 mol%, 4.2 mg), (*S*)-MonoPhos (0.036 mmol, 12 mol%, 9.6 mg), LiO*t*-Bu (0.36 mmol, 1.2 equiv, 28.8 mg), 1,1-bis[(pinacolato)boryl]methane (**2**, 0.45 mmol, 1.5 equiv, 120.6 mg), and Li(acac) (0.30 mmol, 1.0 equiv, 31.8 mg). Subsequently, THF (0.6 mL) was added to the mixture via a syringe and the system was sealed with an open-top teflon faced screw cap. The vial was removed from the glovebox and the reaction mixture was vigorously stirred for one hour. To the system was added a solution of enone (0.3 mmol) in 0.3 mL of THF via a syringe. Then, the reaction mixture was vigorously stirred at room temperature for 11 hours. Upon completion of the reaction, brine (20 mL) was added in one portion to quench the reaction. The aqueous phase was separated and extracted with ethyl acetate (3 × 20 mL). The combined organic phase was dried over anhydrous sodium sulfate (Na_2SO_4), filtered, and concentrated under reduced pressure. The crude product was purified by flash column chromatography on silica gel (EtOAc/hexanes). For the preparation of racemic samples, triphenylphosphine (12 mol%) or a mixture of (*R*)-MonoPhos (6 mol%) and (*S*)-MonoPhos (6 mol%) was used in place of (*S*)-MonoPhos (12 mol%).

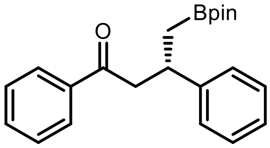

(*S*)-1,3-diphenyl-4-(4,4,5,5-tetramethyl-1,3,2-dioxaborolan-2-yl)butan-1-one (3a)
Following general procedure, the crude product was purified by silica gel chromatography (hexanes : DCM = 100 : 0 → 5 : 95) to provide **3a** as a yellowish liquid (74 mg, 70%, 92% ee). An identical reaction was performed on a 3 mmol scale to provide similar results (714 mg, 68% yield, 88% ee).

^{1}H NMR (400 MHz, $CDCl_3$) δ 7.92 (m, 2H), 7.55–7.48 (m, 1H), 7.46–7.37 (m, 2H), 7.31–7.20 (m, 4H), 7.18–7.10 (m, 1H), 3.62 (m, *J* = 1H), 3.32 (dd, *J* = 16.1, 7.0 Hz, 1H), 3.26 (dd, *J* = 16.1, 7.2 Hz, 1H), 1.33 (dd, *J* = 15.5, 6.8 Hz, 1H), 1.24 (dd, *J* = 15.5, 8.8 Hz, 1H), 1.10 (s, 6H), 1.08 (s, 6H) ppm.

^{13}C NMR (101 MHz, $CDCl_3$) δ 199.03, 146.31, 137.20, 132.70, 128.37, 128.16, 128.08, 127.22, 126.06, 82.96, 47.69, 37.29, 24.65, 24.56 ppm. The carbon bound to the boron was not detected due to quadrupolar relaxation.

^{11}B NMR (128 MHz, $CDCl_3$) δ 33.5 ppm.

HRMS (ESI) calculated for $[C_{22}H_{27}BO_3+H]^+$: 351.2126, found: 351.2138.

Optical rotation, $[\alpha]_D^{22}$ = –7.9 (c = 1.04, DCM).

Enantiomeric excess, 92% ee was measured by HPLC (CHIRALPAK IA, *n*-hexane : *i*-PrOH = 98.5 : 1.5 → 98 : 2, 1.0 mL/min, wavelength = 210 nm, 30 °C); t_R = 7.34 min (major), t_R = 7.75 min (minor).

[Racemic **3a**]

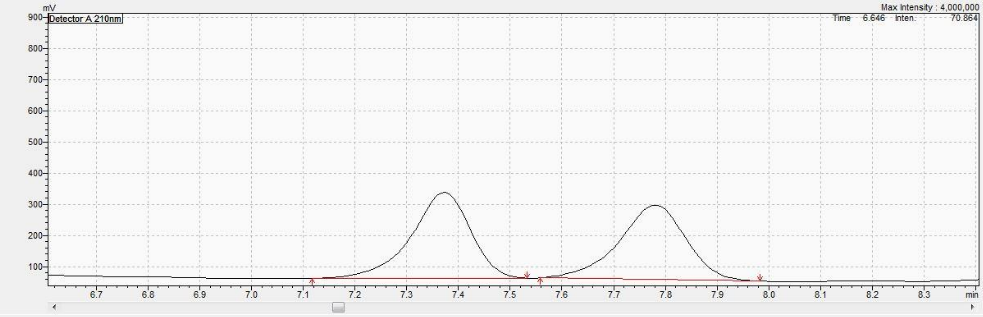

Results View - Peak Table

Peak Table | Compound | Group | Calibration Curve

Peak#	Ret. Time	Area	Height	Mark	Conc.	Unit	ID#	Name	Area%
1	7.373	2196648	276043	M	50.635				50.635
2	7.780	2141511	238902	M	49.365				49.365
Total		4338159	514945		100.000				100.000

[Enantioenriched **3a**]

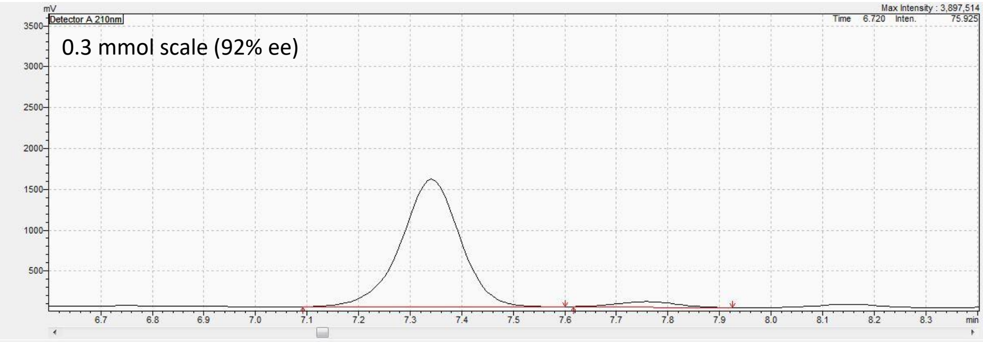

Results View - Peak Table

Peak Table | Compound | Group | Calibration Curve

Peak#	Ret. Time	Area	Height	Mark	Conc.	Unit	ID#	Name	Area%
1	7.341	12348353	1560661	M	95.942				95.942
2	7.758	522253	66884	M	4.058				4.058
Total		12870606	1627545		100.000				100.000

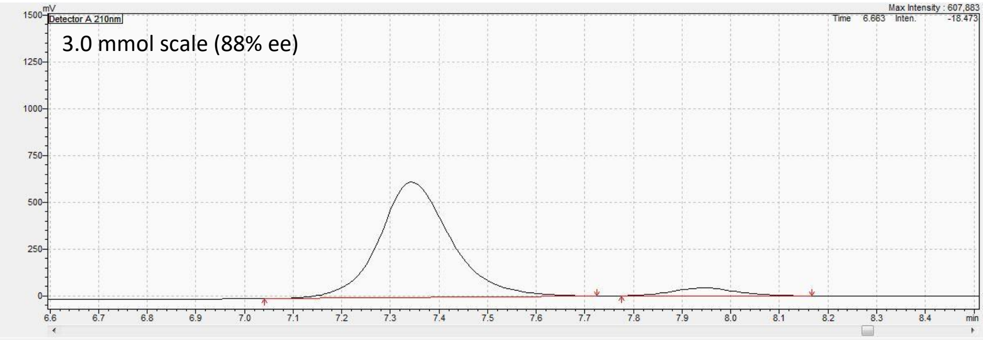

Results View - Peak Table

Peak Table | Compound | Group | Calibration Curve

Peak#	Ret. Time	Area	Height	Mark	Conc.	Unit	ID#	Name	Area%
1	7.344	6323878	616157	M	94.243				94.243
2	7.943	386294	42076	M	5.757				5.757
Total		6710172	658233		100.000				100.000

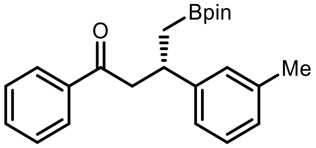

(*S*)-1-phenyl-4-(4,4,5,5-tetramethyl-1,3,2-dioxaborolan-2-yl)-3-(*m*-tolyl)butan-1-one (3b) Following general procedure, the crude product was purified by silica gel chromatography (hexanes : DCM = 100 : 0 → 0 : 100, then DCM : MeOH = 100 : 0 → 99 : 1) to provide **3b** as a yellowish liquid (62 mg, 57%, 93% ee).

^{1}H NMR (400 MHz, $CDCl_3$) δ 7.97–7.89 (m, 2H), 7.56–7.49 (m, 1H), 7.47–7.37 (m, 2H), 7.11 (m, 3H), 6.96 (d, *J* = 7.2 Hz, 1H), 3.58 (m, 1H), 3.32 (dd, *J* = 16.1, 7.1 Hz, 1H), 3.26 (dd, *J* = 16.0, 7.1 Hz, 1H), 2.30 (s, 3H), 1.31 (dd, *J* = 15.5, 6.8 Hz, 1H), 1.21 (dd, *J* = 15.5, 8.8 Hz, 1H), 1.11 (s, 6H), 1.09 (s, 6H) ppm.

^{13}C NMR (101 MHz, $CDCl_3$) δ 199.17, 146.32, 137.56, 137.29, 132.70, 128.39, 128.14, 128.11, 128.10, 126.80, 124.16, 82.97, 47.63, 37.22, 24.69, 24.60, 21.41 ppm.

^{11}B NMR (128 MHz, $CDCl_3$) δ 33.0 ppm.

HRMS (ESI) calculated for $[C_{23}H_{29}BO_3+H]^+$: 365.2282, found: 365.2296.

Optical rotation, $[\alpha]_D^{22}$ = +4.7 (c = 1.23, DCM).

Enantiomeric excess, 93% ee was measured by HPLC (CHIRALPAK IA, *n*-hexane : *i*-PrOH = 99.8 : 0.2, 1.0 mL/min, wavelength = 254 nm, 28 ℃); t_R = 28.06 min (major), t_R = 25.42 min (minor).

[Racemic **3b**]

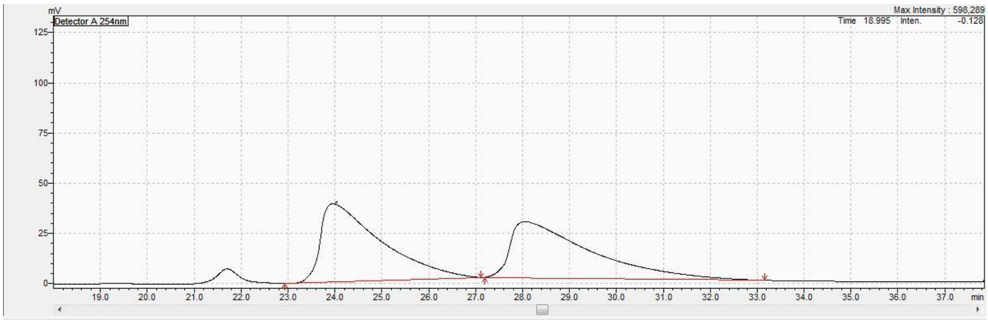

Results View - Peak Table

Peak Table | Compound | Group | Calibration Curve

Peak#	Ret. Time	Area	Height	Mark	Conc.	Unit	ID#	Name	Area%
1	23.955	3481448	39006	M	51.187				51.187
2	28.045	3320035	27960	M	48.813				48.813
Total		6801483	66966		100.000				100.000

[Enantioenriched **3b**]

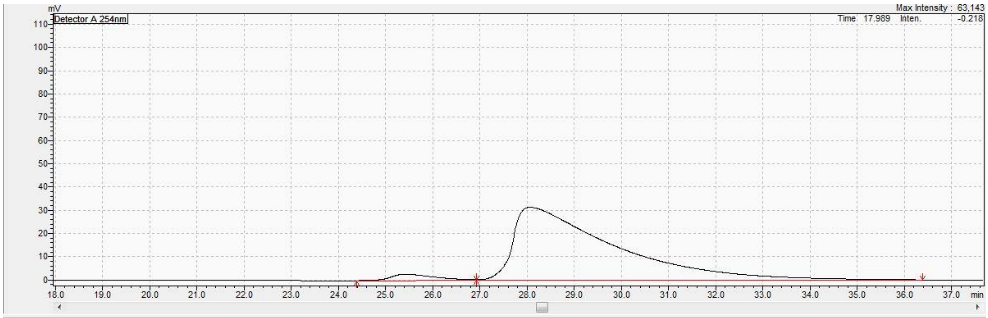

Results View - Peak Table

Peak Table | Compound | Group | Calibration Curve

Peak#	Ret. Time	Area	Height	Mark	Conc.	Unit	ID#	Name	Area%
1	25.420	178359	2730		3.702				3.702
2	28.067	4639812	31408	V	96.298				96.298
Total		4818171	34138		100.000				100.000

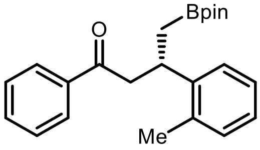

(*S*)-1-phenyl-4-(4,4,5,5-tetramethyl-1,3,2-dioxaborolan-2-yl)-3-(o-tolyl)butan-1-one (3c) Following general procedure, the crude product was purified by silica gel chromatography (hexanes : DCM = 100 : 0 → 0 : 100, then DCM : MeOH = 100 : 0 → 99 : 1) to provide **3c** as a yellowish liquid (38 mg, 35%, 81% ee).

^{1}H NMR (400 MHz, CDCl$_3$) δ 7.92 (m, 2H), 7.52 (m, 1H), 7.41 (m, 2H), 7.27–7.23 (m, 1H), 7.13 (m, 1H), 7.09–6.99 (m, 2H), 3.87 (m, 1H), 3.32–3.19 (m, 2H), 2.39 (s, 3H), 1.29 (dd, *J* = 15.4, 6.6 Hz, 1H), 1.21 (dd, *J* = 15.4, 9.4 Hz, 1H), 1.05 (s, 6H), 1.02 (s, 6H) ppm.

^{13}C NMR (101 MHz, CDCl$_3$) δ 199.29, 144.66, 137.30, 135.70, 132.76, 130.15, 128.44, 128.12, 126.05, 125.77, 125.76, 82.96, 47.45, 32.01, 24.57, 19.76 ppm.

^{11}B NMR (128 MHz, CDCl$_3$) δ 33.0 ppm.

HRMS (ESI) calculated for $[C_{23}H_{29}BO_3+H]^+$: 365.2282, found: 365.2296.

Optical rotation, $[\alpha]_D^{22}$ = –2.0 (c = 0.76, DCM).

Enantiomeric excess, 81% ee was measured by HPLC (CHIRALCEL OD-H, *n*-hexane : *i*-PrOH = 99.3 : 0.7, 1.2 mL/min, wavelength = 254 nm, 28 °C); t_R = 10.23 min (major), t_R = 11.89 min (minor).

[Racemic **3c**]

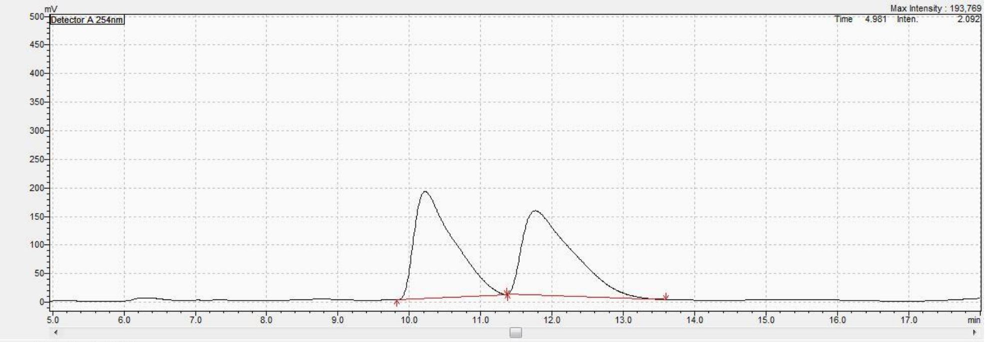

Peak#	Ret. Time	Area	Height	Mark	Conc.	Unit	ID#	Name	Area%
1	10.224	7192715	187123	M	50.113				50.113
2	11.766	7160410	147796	M	49.887				49.887
Total		14353125	334919		100.000				100.000

[Enantioenriched **3c**]

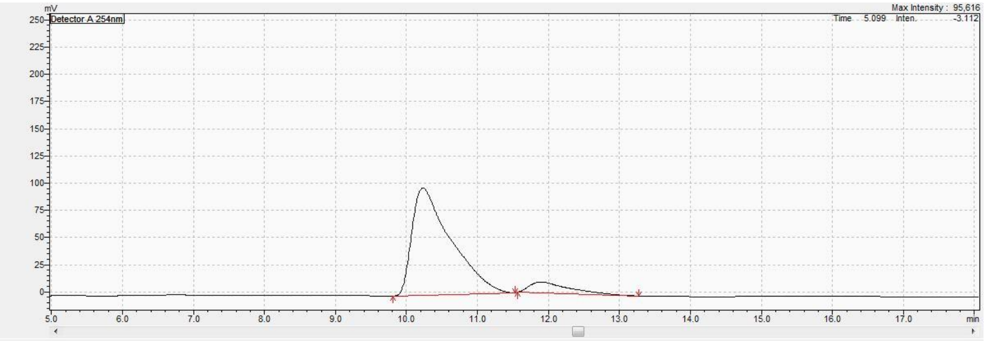

Peak#	Ret. Time	Area	Height	Mark	Conc.	Unit	ID#	Name	Area%
1	10.232	3760225	98709	M	90.454				90.454
2	11.892	396831	10083	M	9.546				9.546
Total		4157055	108792		100.000				100.000

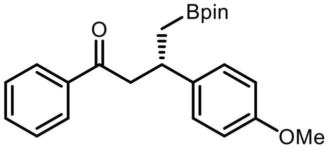

(*S*)-3-(4-methoxyphenyl)-1-phenyl-4-(4,4,5,5-tetramethyl-1,3,2-dioxaborolan-2 yl)butan-1-one (3d) Following general procedure, the crude product was purified by silica gel chromatography (hexanes : DCM = 100 : 0 → 0 : 100, then DCM : MeOH = 100 : 0 → 98 : 2) to provide **3d** as a yellowish liquid (75 mg, 68%, 85% ee).

^{1}H NMR (400 MHz, CDCl$_3$) δ 7.95–7.88 (m, 2H), 7.55–7.49 (m, 1H), 7.46–7.37 (m, 2H), 7.21–7.14 (m, 2H), 6.82–6.75 (m, 2H), 3.75 (s, 3H), 3.55 (m, 1H), 3.29 (dd, *J* = 15.9, 6.8 Hz, 1H), 3.21 (dd, *J* = 15.9, 7.4 Hz, 1H), 1.29 (dd, *J* = 15.5, 6.9 Hz, 1H), 1.20 (dd, *J* = 15.5, 8.8 Hz, 1H), 1.11 (s, 6H), 1.09 (s, 6H) ppm.

^{13}C NMR (101 MHz, CDCl$_3$) δ 199.29, 157.88, 138.52, 137.29, 132.72, 128.43, 128.16, 128.17, 113.60, 83.03, 55.21, 48.01, 36.65, 24.75, 24.63 ppm. The carbon bound to the boron was not detected due to quadrupolar relaxation.

^{11}B NMR (128 MHz, CDCl$_3$) δ 33.3 ppm.

HRMS (ESI) calculated for $[C_{23}H_{29}BO_4+H]^+$: 381.2231, found: 381.2240.

Optical rotation, $[\alpha]_D^{22}$ = +3.98 (c = 0.58, DCM).

Enantiomeric excess, 85% ee was measured by HPLC (CHIRALPAK IA, *n*-hexane : *i*-PrOH = 98.5 : 1.5 → 98 : 2, 1.0 mL/min, wavelength = 210 nm, 30 °C); t_R = 9.85 min (major), t_R = 10.79 min (minor).

[Racemic **3d**]

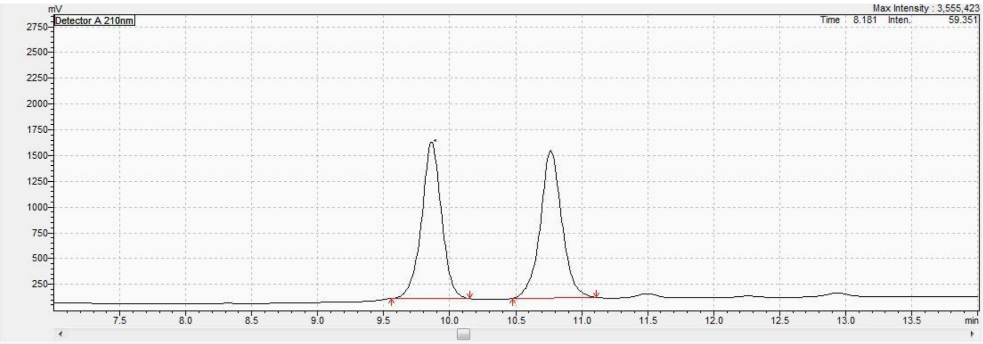

Results View - Peak Table

Peak Table | Compound | Group | Calibration Curve

Peak#	Ret. Time	Area	Height	Mark	Conc.	Unit	ID#	Name	Area%
1	9,862	15998326	1521309	M	49,633				49,633
2	10,764	16234816	1431284	M	50,367				50,367
Total		32233142	2952592		100,000				100,000

[Enantioenriched **3d**]

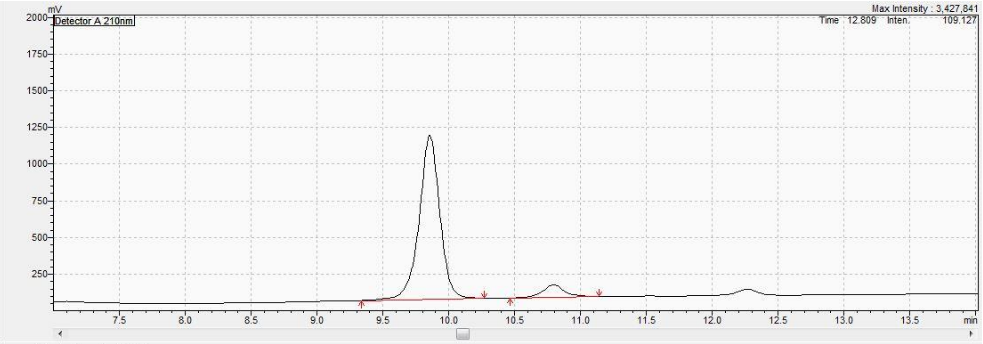

Results View - Peak Table

Peak Table | Compound | Group | Calibration Curve

Peak#	Ret. Time	Area	Height	Mark	Conc.	Unit	ID#	Name	Area%
1	9,855	12155740	1117182	M	92,287				92,287
2	10,795	1015910	85216	M	7,713				7,713
Total		13171651	1202398		100,000				100,000

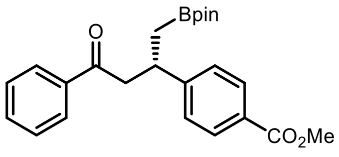

Methyl-(*S*)-4-(4-oxo-4-phenyl-1-(4,4,5,5-tetramethyl-1,3,2-dioxaborolan-2-yl)butan-2-yl)benzoate (3e) Following general procedure, the crude product was purified by silica gel chromatography (hexanes : DCM = 50 : 50 → 0 : 100, DCM : MeOH = 100 : 0 → 98 : 2) to provide **3c** as a colorless liquid (81 mg, 66%, 91% ee).

^{1}H NMR (400 MHz, $CDCl_3$) δ 7.91–7.86 (m, 4H), 7.57–7.49 (m, 1H), 7.46–7.38 (m, 2H), 7.37–7.31 (m, 2H), 3.88 (s, 3H), 3.68 (m, 1H), 3.34 (dd, *J* = 16.4, 6.6 Hz, 1H), 3.27 (dd, *J* = 16.4, 7.5 Hz, 1H), 1.32 (dd, *J* = 15.6, 6.7 Hz, 1H), 1.22 (dd, *J* = 14.5, 9.4 Hz, 1H), 1.10 (s, 6H), 1.07 (s, 6H) ppm.

^{13}C NMR (101 MHz, $CDCl_3$) δ 198.81, 167.26, 152.06, 137.21, 133.10, 129.81, 128.67, 128.25, 128.18, 127.55, 83.35, 52.08, 47.38, 37.39, 24.89, 24.76 ppm. The carbon bound to the boron was not detected due to quadrupolar relaxation.

^{11}B NMR (128 MHz, $CDCl_3$) δ 33.39 ppm.

HRMS (ESI) calculated for $[C_{24}H_{29}BO_5+H]^+$: 409.2180, found: 409.2194.

Optical rotation, $[\alpha]_D^{22}$ = –2.49 (c = 0.88, DCM).

Enantiomeric excess, 91% ee was measured by HPLC (CHIRALCEL OD, *n*-hexane : *i*-PrOH = 99 : 1, 1.0 mL/min, wavelength = 240 nm, 28 °C); t_R = 22.69 min (major), t_R = 25.23 min (minor).

[Racemic **3e**]

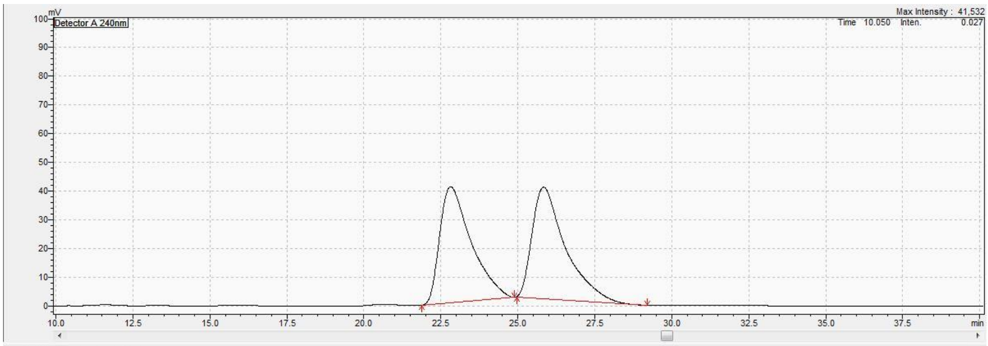

Results View - Peak Table

Peak Table | Compound | Group | Calibration Curve

Peak#	Ret. Time	Area	Height	Mark	Conc.	Unit	ID#	Name	Area%
1	22.817	2878463	40451	M	49.652				49.652
2	25.835	2918797	38833	M	50.348				50.348
Total		5797260	79284		100.000				100.000

[Enantioenriched **3e**]

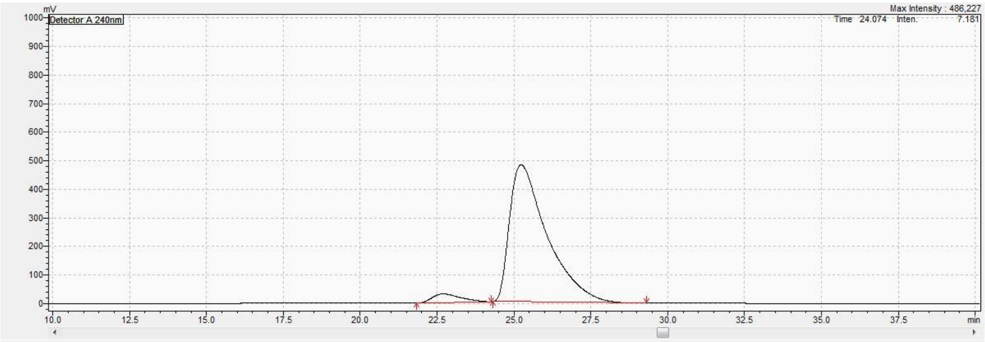

Results View - Peak Table

Peak Table | Compound | Group | Calibration Curve

Peak#	Ret. Time	Area	Height	Mark	Conc.	Unit	ID#	Name	Area%
1	22.695	1979924	30581	M	4.625				4.625
2	25.235	40824915	480437	M	95.375				95.375
Total		42804839	511017		100.000				100.000

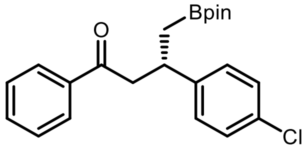

(*S*)-3-(4-chlorophenyl)-1-phenyl-4-(4,4,5,5-tetramethyl-1,3,2-dioxaborolan-2-yl)butan-1-one (3f) Following general procedure, the crude product was purified by silica gel chromatography (hexanes : DCM = 100 : 0 → 0 : 100, then DCM : MeOH = 100 : 0 → 99 : 1) to provide **3f** as a yellowish liquid (86 mg, 76%, 94% ee).

^{1}H NMR (400 MHz, $CDCl_3$) δ 7.96–7.85 (m, 2H), 7.55–7.50 (m, 1H), 7.42 (m, 2H), 7.22–7.19 (m, 4H), 3.65–3.55 (m, 1H), 3.31 (dd, *J* = 16.3, 6.5 Hz, 1H), 3.23 (dd, *J* = 16.3, 7.7 Hz, 1H), 1.30 (dd, *J* = 15.6, 6.8 Hz, 1H), 1.19 (dd, *J* = 15.6, 8.7 Hz, 1H), 1.12 (s, 6H), 1.10 (s, 6H) ppm.

^{13}C NMR (101 MHz, $CDCl_3$) δ 198.73, 144.86, 137.08, 132.87, 131.65, 128.69, 128.47, 128.27, 128.07, 83.14, 47.46, 36.67, 24.72, 24.60 ppm. The carbon bound to the boron was not detected due to quadrupolar relaxation.

^{11}B NMR(128 MHz, $CDCl_3$) δ 33.2 ppm.

HRMS (ESI) calculated for $[C_{22}H_{26}BClO_3+H]^+$: 385.1736, found: 385.1737.

Optical rotation, $[\alpha]_D^{22}$ = –3.42 (c = 0.99, DCM).

Enantiomeric excess, 94% ee was measured by HPLC (CHIRALPAK IA, *n*-hexane : *i*-PrOH = 98.5 : 1.5 → 98 : 2, 1.0 mL/min, wavelength = 210 nm, 30 ℃); t_R = 8.50 min (major), t_R = 9.43 min (minor).

[Racemic **3f**]

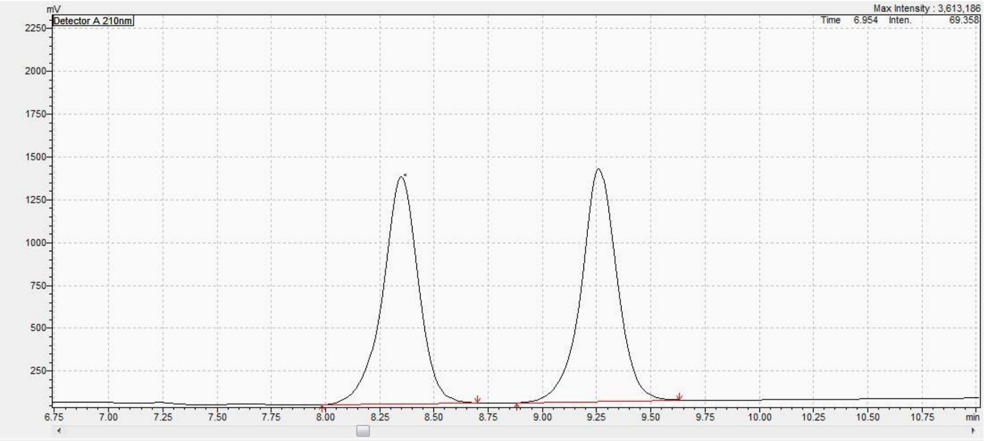

Results View - Peak Table

Peak Table | Compound | Group | Calibration Curve

Peak#	Ret. Time	Area	Height	Mark	Conc.	Unit	ID#	Name	Area%
1	8.349	15321225	1329103	M	49.808				49.808
2	9.261	15439537	1356526	M	50.192				50.192
Total		30760762	2685629		100.000				100.000

[Enantioenriched **3f**]

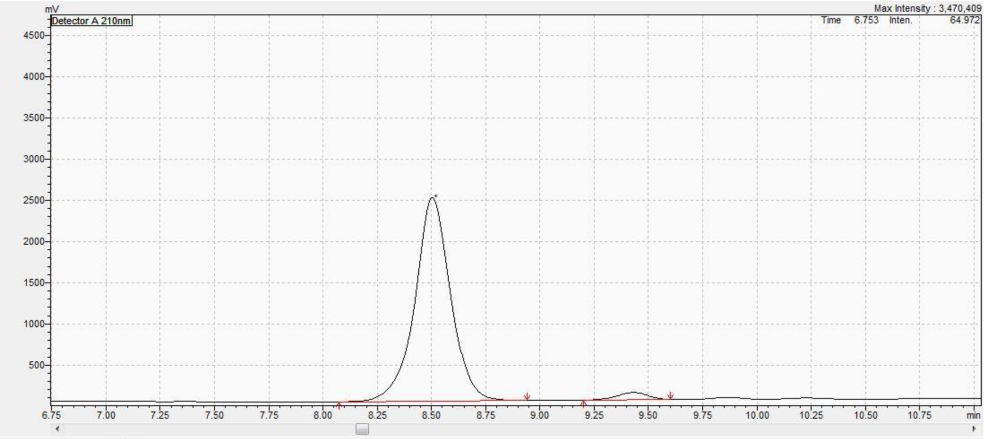

Results View - Peak Table

Peak Table | Compound | Group | Calibration Curve

Peak#	Ret. Time	Area	Height	Mark	Conc.	Unit	ID#	Name	Area%
1	8.505	28524221	2478403	M	96.990				96.990
2	9.433	885169	89653	M	3.010				3.010
Total		29409390	2568056		100.000				100.000

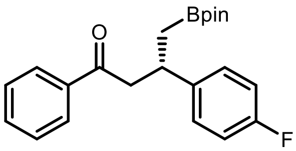

(*S*)-3-(4-fluorophenyl)-1-phenyl-4-(4,4,5,5-tetramethyl-1,3,2-dioxaborolan-2-yl)butan-1-one (3g) Following general procedure, the crude product was purified by silica gel chromatography (hexanes : DCM = 100 : 0 → 0 : 100, then DCM : MeOH = 100 : 0 → 99 : 1) to provide **3g** as an orange liquid (89 mg, 77%, 91% ee).

^{1}H NMR (400 MHz, $CDCl_3$) δ 7.94–7.88 (m, 2H), 7.55–7.49 (m, 1H), 7.45–7.38 (m, 2H), 7.25–7.19 (m, 2H), 6.97–6.87 (m, 2H), 3.65–3.56 (m, 1H), 3.30 (dd, *J* = 16.1, 6.6 Hz, 1H), 3.22 (dd, *J* = 16.1, 7.6 Hz, 1H), 1.30 (dd, *J* = 15.5, 6.7 Hz, 1H), 1.20 (dd, *J* = 15.5, 9.0 Hz, 1H), 1.11 (s, 6H), 1.09 (s, 6H) ppm.

^{13}C NMR (101 MHz, $CDCl_3$) δ 198.91, δ 161.27 (d, *J* = 243.6 Hz), 141.96 (d, *J* = 3.2 Hz), 137.15, 132.83, 128.70 (d, *J* = 7.8 Hz), 128.46, 128.08, 114.87 (d, *J* = 21.1 Hz), 83.09, 47.79, 36.64, 24.71, 24.59 ppm. The carbon bound to the boron was not detected due to quadrupolar relaxation.

^{11}B NMR (128 MHz, $CDCl_3$) δ 33.1 ppm.

^{19}F NMR (376 MHz, $CDCl_3$) δ –117.38 ppm.

HRMS (ESI) calculated for $[C_{22}H_{26}BFO_3+H]^+$: 369.2032, found: 369.2044.

Optical rotation, $[\alpha]_D^{22}$ = –0.85 (c = 1.50, DCM).

Enantiomeric excess, 91% ee was measured by HPLC (CHIRALPAK IA, *n*-hexane : *i*-PrOH = 99 : 1, 1.0 mL/min, wavelength = 254 nm, 28 °C); t_R = 8.47 min (major), t_R = 9.54 min (minor).

[Racemic **3g**]

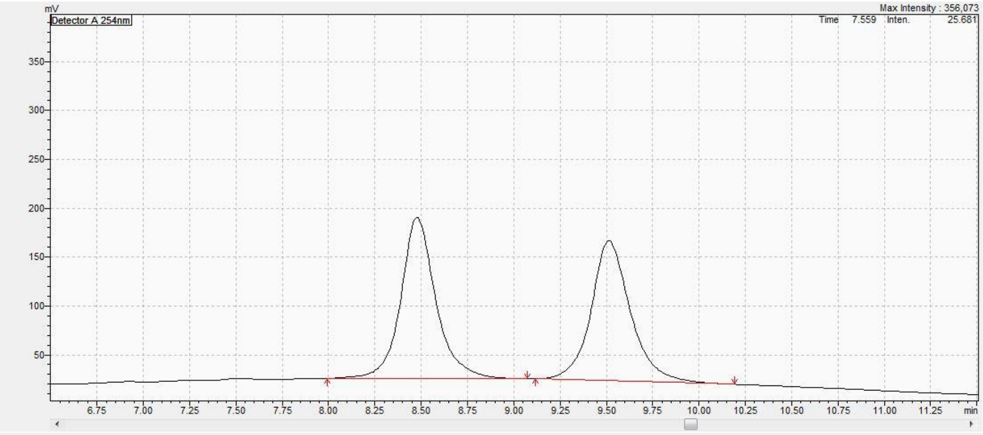

Results View - Peak Table

Peak Table | Compound | Group | Calibration Curve

Peak#	Ret. Time	Area	Height	Mark	Conc.	Unit	ID#	Name	Area%
1	8.478	2179907	164913	M	50.495				50.495
2	9.513	2137162	143356	M	49.505				49.505
Total		4317069	308269		100.000				100.000

[Enantioenriched **3g**]

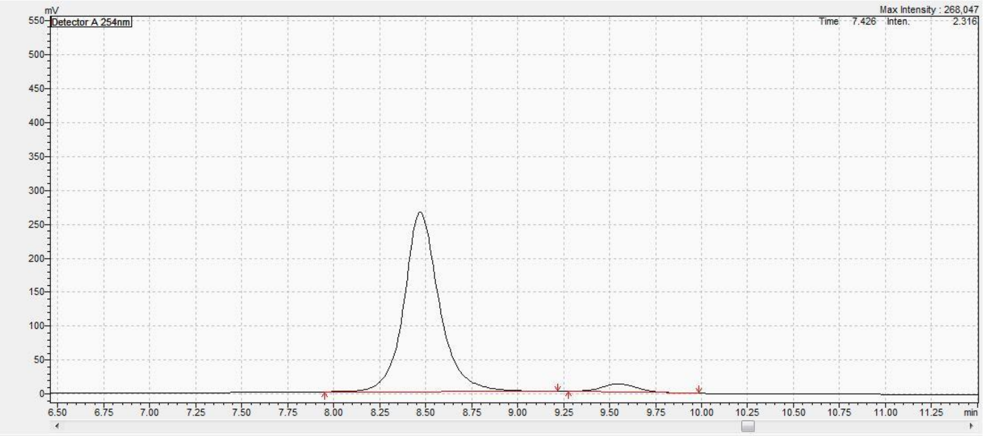

Results View - Peak Table

Peak Table | Compound | Group | Calibration Curve

Peak#	Ret. Time	Area	Height	Mark	Conc.	Unit	ID#	Name	Area%
1	8.470	3526870	264237	M	95.549				95.549
2	9.548	164284	12274	M	4.451				4.451
Total		3691154	276512		100.000				100.000

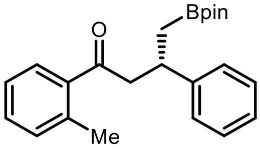

(*S*)-3-phenyl-4-(4,4,5,5-tetramethyl-1,3,2-dioxaborolan-2-yl)-1-(o-tolyl)butan-1-one (3h)
Following general procedure, the crude product was purified by silica gel chromatography (hexanes : DCM = 100 : 0 → 0 : 100) to provide **3h** as a yellowish liquid (81 mg, 74%, 93% ee).

^{1}H NMR (400 MHz, CDCl$_3$) δ 7.51–7.55 (m, 1H), 7.28–7.34 (m, 1H), 7.27–7.09 (m, 6H), 3.60–3.48 (m, 1H), 3.29 (dd, *J* = 16.0, 6.7 Hz, 1H), 3.16 (dd, *J* = 16.0, 8.0 Hz, 1H), 2.26 (s, 3H), 1.29 (dd, *J* = 15.5, 7.0 Hz, 1H), 1.21 (dd, *J* = 15.5, 8.7 Hz, 1H), 1.11 (s, 6H), 1.09 (s, 6H) ppm.

^{13}C NMR (101 MHz, CDCl$_3$) δ 203.72, 146.01, 138.59, 137.78, 131.66, 130.79, 128.19, 128.15, 127.34, 126.12, 125.41, 83.04, 50.79, 37.58, 24.71, 24.64, 20.71 ppm. The carbon bound to the boron was not detected due to quadrupolar relaxation.

^{11}B NMR (128 MHz, CDCl$_3$) δ 33.0 ppm.

HRMS (ESI) calculated for $[C_{23}H_{29}BO_3+H]^+$: 365.2282, found: 365.2296.

Optical rotation, $[\alpha]_D^{22}$ = –2.6 (c = 1.05, DCM).

Enantiomeric excess, 93% ee was measured by HPLC (CHIRALPAK IA, *n*-hexane : *i*-PrOH = 99 : 1, 0.6 mL/min, wavelength = 210 nm, 28 °C); t_R = 10.66 min (major), t_R = 9.77 min (minor).

[Racemic **3h**]

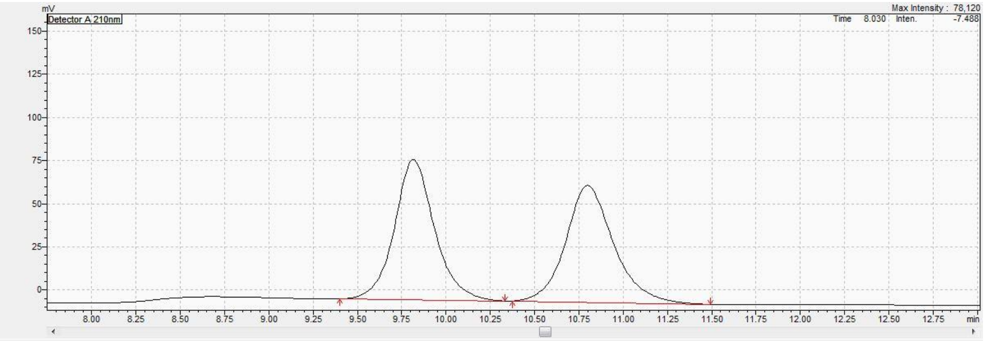

Results View - Peak Table

Peak Table | Compound | Group | Calibration Curve

Peak#	Ret. Time	Area	Height	Mark	Conc.	Unit	ID#	Name	Area%
1	9.816	1279686	81291	M	50.077				50.077
2	10.799	1275735	67706	M	49.923				49.923
Total		2555421	148997		100.000				100.000

[Enantioenriched **3h**]

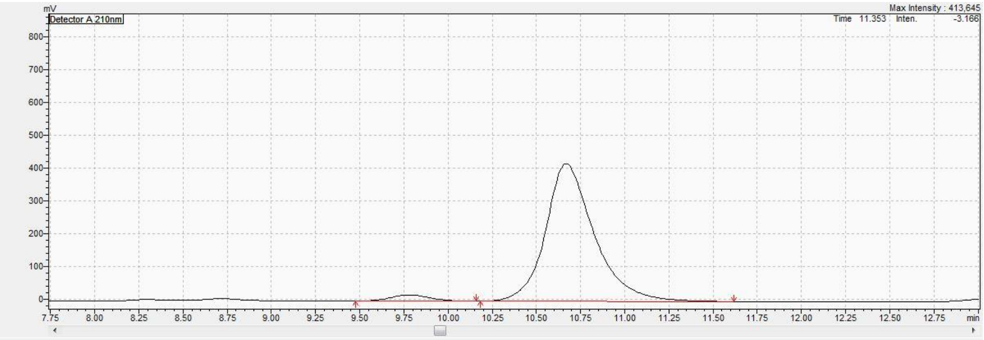

Results View - Peak Table

Peak Table | Compound | Group | Calibration Curve

Peak#	Ret. Time	Area	Height	Mark	Conc.	Unit	ID#	Name	Area%
1	9.778	294910	19016	M	3.535				3.535
2	10.667	8047359	419527	M	96.465				96.465
Total		8342269	438542		100.000				100.000

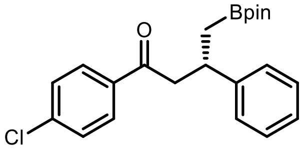

(*S*)-1-(4-chlorophenyl)-3-phenyl-4-(4,4,5,5-tetramethyl-1,3,2-dioxaborolan-2-yl)butan-1-one (3i) Following general procedure, the crude product was purified by silica gel chromatography (hexanes : EtOAc = 10 : 1) to provide **3i** as a colorless oil (85 mg, 74%, 89% ee).

^{1}H NMR (400 MHz, $CDCl_3$) δ 7.81–7.75 (m, 2H), 7.35–7.29 (m, 2H), 7.21–7.15 (m, 4H), 7.11–7.04 (m, 1H), 3.50 (m, 1H), 3.22 (dd, *J* = 16.0, 6.8 Hz, 1H), 3.14 (dd, *J* = 15.9, 7.4 Hz, 1H), 1.24 (dd, *J* = 15.5, 7.0 Hz, 1H), 1.15 (dd, *J* = 15.9, 8.6 Hz, 1H), 1.03 (s, 6H), 1.01 (s, 6H) ppm.

^{13}C NMR (101 MHz, $CDCl_3$) δ 197.95, 146.11, 139.16, 135.60, 129.57, 128.72, 128.25, 127.21, 126.20, 83.08, 47.68, 37.46, 24.70, 24.61 ppm. The carbon bound to the boron was not detected due to quadrupolar relaxation.

^{11}B NMR (128 MHz, $CDCl_3$) δ 32.8 ppm.

HRMS (ESI) calculated for $[C_{22}H_{26}BClO_3+H]^+$: 385.1736, found: 385.1734.

Optical rotation, $[\alpha]_D^{22}$ = +0.31 (c = 1.5, DCM).

Enantiomeric excess, 89% ee was measured by HPLC (CHIRALPAK IA, *n*-hexane : *i*-PrOH = 99 : 1, 1.0 mL/min, wavelength = 254 nm, 28 °C); t_R = 9.89 min (major), t_R = 12.11 min (minor).

[Racemic **3i**]

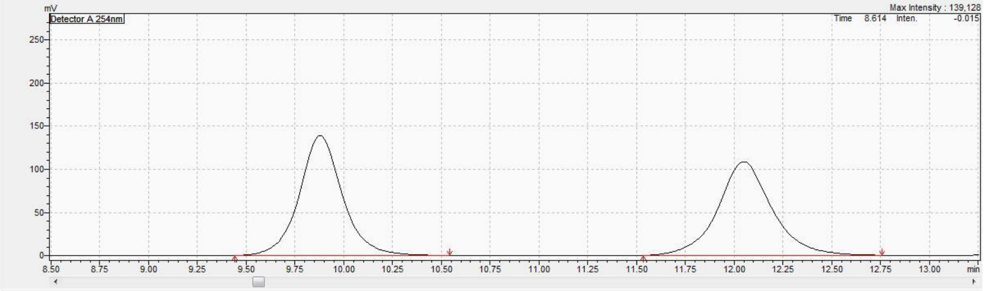

Results View - Peak Table

Peak Table | Compound | Group | Calibration Curve

Peak#	Ret. Time	Area	Height	Mark	Conc.	Unit	ID#	Name	Area%
1	9,879	2119173	138790	M	50,240				50,240
2	12,048	2098908	108175	M	49,760				49,760
Total		4218081	246964		100,000				100,000

[Enantioenriched **3i**]

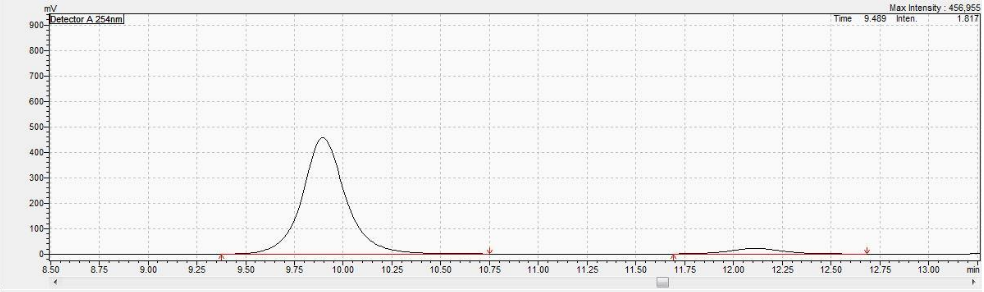

Results View - Peak Table

Peak Table | Compound | Group | Calibration Curve

Peak#	Ret. Time	Area	Height	Mark	Conc.	Unit	ID#	Name	Area%
1	9,894	7075917	456580	M	94,539				94,539
2	12,112	408699	21539	M	5,461				5,461
Total		7484616	478119		100,000				100,000

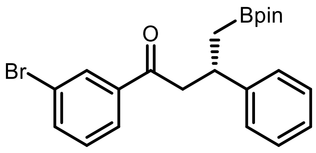

(*S*)-1-(3-bromophenyl)-3-phenyl-4-(4,4,5,5-tetramethyl-1,3,2-dioxaborolan-2-yl)butan-1-one (3j) Following general procedure, the crude product was purified by silica gel chromatography (hexanes : DCM = 50 : 50 → 0 : 100, DCM : MeOH = 100 : 0 → 98 : 2) to provide **3j** as a pale yellow oil (90 mg, 70%, 93% ee).

^{1}H NMR (500 MHz, CDCl$_3$) δ 8.06–8.02 (m, 1H), 7.85–7.80 (m, 1H), 7.67–7.61 (m, 1H), 7.32–7.27 (m, 1H), 7.26–7.21 (m, 4H), 7.18–7.11 (m, 1H), 3.63–3.52 (m, 1H), 3.30 (dd, J = 16.0, 6.8 Hz, 1H), 3.20 (dd, J = 16.0, 7.4 Hz, 1H), 1.31 (dd, J = 15.6, 7.0 Hz, 1H), 1.23 (dd, J = 15.8, 8.6 Hz, 1H), 1.11 (s, 6H), 1.09 (s, 6H).

^{13}C NMR (126 MHz, CDCl$_3$) δ 197.79, 135.58, 131.24, 130.02, 128.48, 128.27, 127.21, 126.65, 126.55, 126.24, 122.82, 83.12, 47.75, 37.39, 24.70, 24.63 ppm. The carbon bound to the boron was not detected due to quadrupolar relaxation.

^{11}B NMR (128 MHz, CDCl$_3$) δ 33.5 ppm.

HRMS (ESI) calculated for $[C_{22}H_{26}BBrO_3+H]^+$: 429.1231, found: 429.1234.

Optical rotation, $[\alpha]_D^{22}$ = –4.14 (c = 1.2, DCM).

Enantiomeric excess, 93% ee was measured by HPLC (CHIRALPAK IA, *n*-hexane : *i*-PrOH = 99.5 : 0.5, 1.0 mL/min, wavelength = 254 nm, 28 °C); t_R = 12.25min (major), t_R = 10.52min (minor).

[Racemic **3j**]

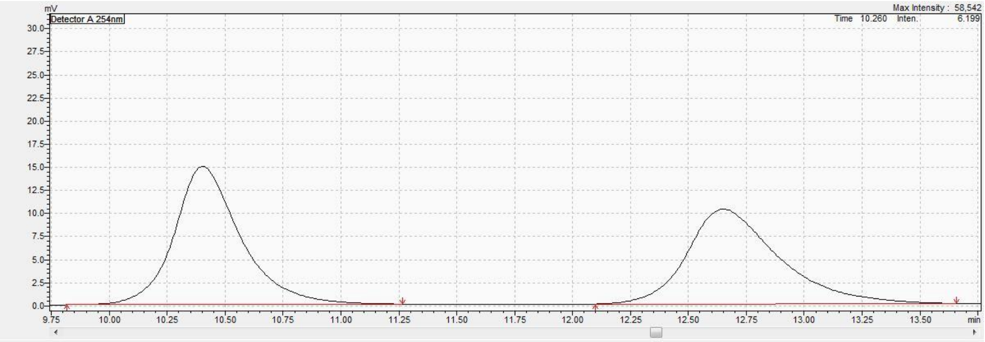

Peak#	Ret. Time	Area	Height	Mark	Conc.	Unit	ID#	Name	Area%
1	10,403	298824	14949	M	51,478				51,478
2	12,650	281668	10234	M	48,522				48,522
Total		580492	25183		100,000				100,000

[Enantioenriched **3j**]

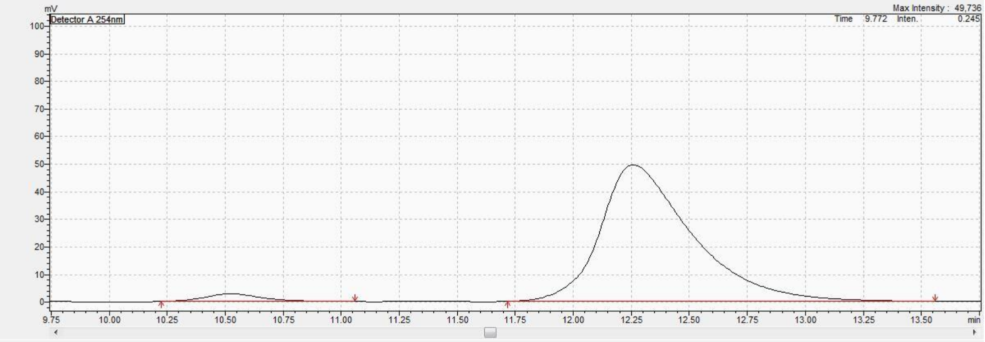

Peak#	Ret. Time	Area	Height	Mark	Conc.	Unit	ID#	Name	Area%
1	10,525	47877	2782	M	3,390				3,390
2	12,258	1364521	49464	M	96,610				96,610
Total		1412398	52246		100,000				100,000

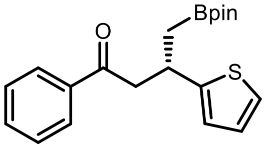

(*S*)-1-phenyl-4-(4,4,5,5-tetramethyl-1,3,2-dioxaborolan-2-yl)-3-(thiophen-2-yl)butan-1-one (3k) Following general procedure, the crude product was purified by silica gel chromatography (hexanes : DCM = 50 : 50 → 0 : 100, DCM : MeOH = 100 : 0 → 97 : 3) to provide **3k** as a colorless liquid (80 mg, 75%, 92% ee).

^{1}H NMR (500 MHz, $CDCl_3$) δ 7.98–7.90 (m, 2H), 7.56–7.51 (m, 1H), 7.47–7.40 (m, 2H), 7.10–7.03 (m, 1H), 6.89–6.82 (m, 2H), 4.01–3.91 (m, 1H), 3.36 (dd, *J* = 14.9, 5.4 Hz, 1H), 3.32 (dd, *J* = 14.9, 5.6 Hz, 1H), 1.37 (dd, *J* = 15.7, 6.7 Hz, 1H), 1.31 (dd, *J* = 15.6, 8.3 Hz, 1H), 1.15 (s, 6H), 1.13 (s, 6H) ppm.

^{13}C NMR (126 MHz, $CDCl_3$) δ 198.69, 150.56, 137.15, 132.87, 128.46, 128.13, 126.30, 123.33, 122.64, 83.17, 48.35, 32.62, 24.71, 24.65 ppm. The carbon bound to the boron was not detected due to quadrupolar relaxation.

^{11}B NMR (128 MHz, $CDCl_3$) δ 31.87 ppm.

HRMS (ESI) calculated for $[C_{20}H_{25}BO_3S+H]^+$: 357.1690, found: 357.1698.

Optical rotation, $[\alpha]_D^{22}$ = +4.31 (c = 1.01, DCM).

Enantiomeric excess, 92% ee was measured by HPLC (CHIRALCEL OD, *n*-hexane : *i*-PrOH = 99.9 : 0.1, 0.2 mL/min, wavelength = 240 nm, 25 °C); t_R = 25.24 min (major), t_R = 23.20 min (minor). Determined on equipment combined an Agilent Technologies 1200 series Infinity LC and Hewlett Packard 1100 series LC.

[Racemic **3k**]

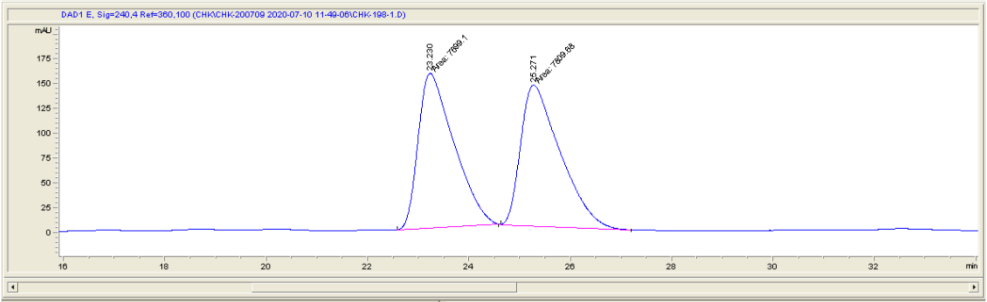

File Information

LC-File	CHK-198-1.D
File Path	C:\CHEM32\1\DATA\CHK\CHK-200709 2020-07-10 11-49-06\
Date	10-Jul-20, 11:50:22

#	Time	Area	Height	Width	Area%	Symmetry
1	23.23	7699.1	156.5	0.8201	49.643	0.497
2	25.271	7809.7	143	0.9104	50.357	0.475

[Enantioenriched **3k**]

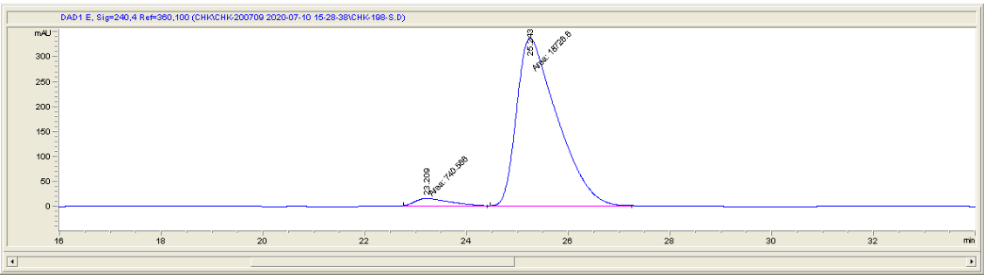

File Information

LC-File	CHK-198-S.D
File Path	C:\CHEM32\1\DATA\CHK\CHK-200709 2020-07-10 15-28-38\
Date	10-Jul-20, 15:29:54

#	Time	Area	Height	Width	Area%	Symmetry
1	23.209	740.6	15.3	0.8067	3.804	0.473
2	25.243	18726.6	337.4	0.925	96.196	0.478

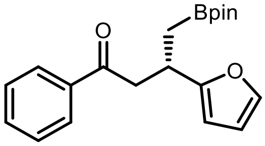

(*S*)-3-(furan-2-yl)-1-phenyl-4-(4,4,5,5-tetramethyl-1,3,2-dioxaborolan-2-yl)butan-1-one (3l) Following general procedure, the crude product was purified by silica gel chromatography (hexanes : DCM = 100 : 0 → 0 : 100, then DCM : MeOH = 100 : 0 → 99 : 1) to provide **3l** as a yellowish liquid (77 mg, 76%, 94% ee).

^{1}H NMR (400 MHz, $CDCl_3$) δ 7.99–7.92 (m, 2H), 7.57–7.50 (m, 1H), 7.47–7.40 (m, 2H), 7.29–7.21 (m, 1H), 6.25–6.18 (m, 1H), 6.01 (d, *J* = 3.2 Hz, 1H), 3.79–3.68 (m, 1H), 3.37 (dd, *J* = 16.3, 6.7 Hz, 1H), 3.25 (dd, *J* = 16.3, 7.1 Hz, 1H), 1.25 (d, *J* = 7.3 Hz, 2H), 1.19 (s, 6H), 1.17 (s, 6H) ppm.

^{13}C NMR (101 MHz, $CDCl_3$) δ 198.86, 159.11, 140.68, 137.19, 132.82, 128.44, 128.15, 109.87, 104.26, 83.13, 44.68, 30.79, 24.78, 24.70 ppm. The carbon bound to the boron was not detected due to quadrupolar relaxation.

^{11}B NMR (128 MHz, $CDCl_3$) δ 33.1 ppm.

HRMS (ESI) calculated for $[C_{20}H_{25}BO_4+H]^+$: 341.1918, found: 341.1930.

Optical rotation, $[\alpha]_D^{22}$ = –9.5 (c = 0.79, DCM).

Enantiomeric excess, 94% ee was measured by HPLC (CHIRALCEL OD-H, *n*-hexane : *i*-PrOH = 99.8 : 0.2, 1.0 mL/min, wavelength = 210 nm, 28 °C); t_R = 19.89 min (major), t_R = 25.00 min (minor).

[Racemic **3l**]

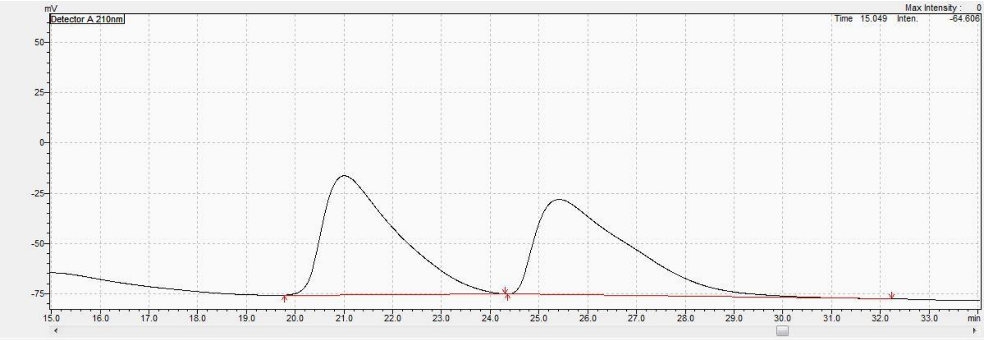

Results View - Peak Table

Peak Table | Compound | Group | Calibration Curve

Peak#	Ret. Time	Area	Height	Mark	Conc.	Unit	ID#	Name	Area%
1	21,003	6254961	59340	M	49,927				49,927
2	25,408	6273157	47444	M	50,073				50,073
Total		12528118	106783		100,000				100,000

[Enantioenriched **3l**]

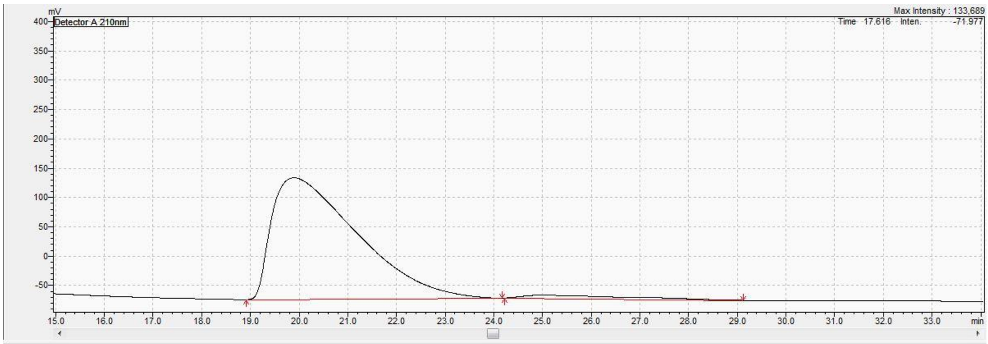

Results View - Peak Table

Peak Table | Compound | Group | Calibration Curve

Peak#	Ret. Time	Area	Height	Mark	Conc.	Unit	ID#	Name	Area%
1	19,894	25252973	207217	M	96,760				96,760
2	25,005	845546	5506	M	3,240				3,240
Total		26098519	212723		100,000				100,000

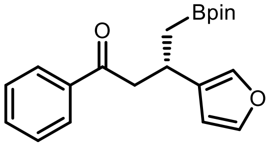

(*S*)-3-(furan-3-yl)-1-phenyl-4-(4,4,5,5-tetramethyl-1,3,2-dioxaborolan-2-yl)butan-1-one (3m) Following general procedure, the crude product was purified by silica gel chromatography (hexanes : DCM = 100 : 0 → 0 : 100, then DCM : MeOH = 100 : 0 → 99 : 1) to provide **3m** as a yellowish liquid (76 mg, 74%, 93% ee).

^{1}H NMR (400 MHz, $CDCl_3$) δ 7.98–7.90 (m, 2H), 7.56–7.50 (m, 1H), 7.47–7.40 (m, 2H), 7.30–7.27 (m, 1H), 7.25–7.21 (m, 1H), 6.33 (m, 1H), 3.62–3.51 (m, 1H), 3.26 (dd, *J* = 16.1, 6.5 Hz, 1H), 3.16 (dd, *J* = 16.1, 7.5 Hz, 1H), 1.22 (m, 2H), 1.17 (s, 6H), 1.16 (s, 6H).

^{13}C NMR (101 MHz, $CDCl_3$) δ 199.21, 142.56, 138.50, 137.28, 132.82, 129.90, 128.47, 128.15, 109.67, 83.13, 46.95, 27.86, 24.76, 24.68 ppm. The carbon bound to the boron was not detected due to quadrupolar relaxation.

^{11}B NMR (128 MHz, $CDCl_3$) δ 33.10 ppm.

HRMS (ESI) calculated for $[C_{20}H_{25}BO_4+H]^+$: 341.1918, found: 341.1921.

Optical rotation, $[\alpha]_D^{22}$ = +0.59 (c = 0.71, DCM).

Enantiomeric excess, 93% ee was measured by HPLC (CHIRALPAK IA, *n*-hexane : *i*-PrOH = 99.8 : 0.2, 1.0 mL/min, wavelength = 254 nm, 30 °C); t_R = 30.71 min (major), t_R = 27.41 min (minor).

[Racemic **3m**]

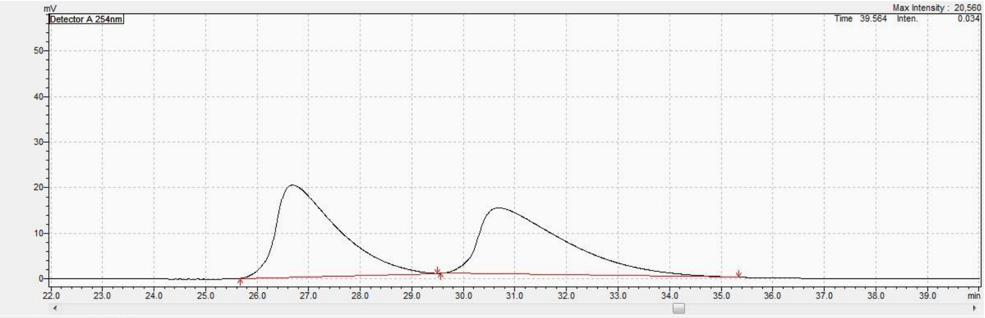

Results View - Peak Table

Peak Table | Compound | Group | Calibration Curve

Peak#	Ret. Time	Area	Height	Mark	Conc.	Unit	ID#	Name	Area%
1	26.688	1681766	20165	M	49.893				49.893
2	30.684	1689004	14518	M	50.107				50.107
Total		3370770	34683		100.000				100.000

[Enantioenriched **3m**]

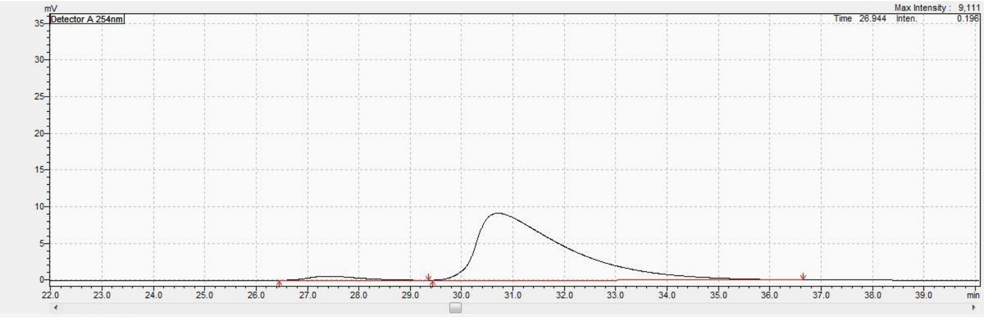

Results View - Peak Table

Peak Table | Compound | Group | Calibration Curve

Peak#	Ret. Time	Area	Height	Mark	Conc.	Unit	ID#	Name	Area%
1	27.410	40100	575	M	3.496				3.496
2	30.705	1106878	9127	M	96.504				96.504
Total		1146978	9702		100.000				100.000

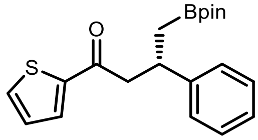

(*S*)-3-phenyl-4-(4,4,5,5-tetramethyl-1,3,2-dioxaborolan-2-yl)-1-(thiophen-2-yl)butan-1-one (3n). Following general procedure, the crude product was purified by silica gel chromatography (hexanes : EtOAc = 100 : 0 → 85 : 15) to provide **3n** as a colorless liquid (75 mg, 70%, 88% ee).

^{1}H NMR (500 MHz, $CDCl_3$) δ 7.67 (d, *J* = 3.5 Hz, 1H), 7.57 (d, *J* = 4.8 Hz, 1H), 7.29–7.20 (m, 4H), 7.17–7.10 (m, 1H), 7.09–7.04 (m, 1H), 3.65–3.55 (m, 1H), 3.26–3.14 (m, 2H), 1.32 (dd, *J* = 15.0, 8.8 Hz, 1H), 1.23 (dd, 1H), 1.09 (s, 6H), 1.07 (s, 6H) ppm.

^{13}C NMR (126 MHz, $CDCl_3$) δ 191.98, 146.08, 144.87, 133.30, 131.80, 128.22, 127.88, 127.24, 126.17, 83.03, 48.57, 37.80, 24.69, 24.58 ppm. The carbon bound to the boron was not detected due to quadrupolar relaxation.

^{11}B NMR (128 MHz, $CDCl_3$) δ 33.27 ppm.

HRMS (ESI) calculated for $[C_{20}H_{25}BO_3S+H]^+$: 357.1690, found: 357.1692.

Optical rotation, $[\alpha]_D^{22}$ = +13.7 (c = 1.42, DCM).

Enantiomeric excess, 88% ee was measured by HPLC (CHIRALCEL OJ-H, *n*-hexane : *i*-PrOH = 95 : 5, 1.0 mL/min, wavelength = 210 nm, 28 °C); t_R = 19.10 min (major), t_R = 14.35 min (minor).

[Racemic **3n**]

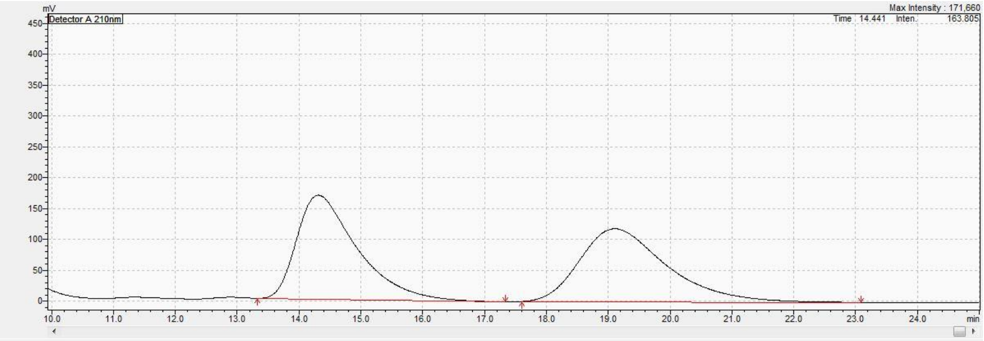

Results View - Peak Table

Peak Table | Compound | Group | Calibration Curve

Peak#	Ret. Time	Area	Height	Mark	Conc.	Unit	ID#	Name	Area%
1	14,312	11437163	168628	M	49,804				49,804
2	19,107	11527059	118299	M	50,196				50,196
Total		22964222	286928		100,000				100,000

[Enantioenriched **3n**]

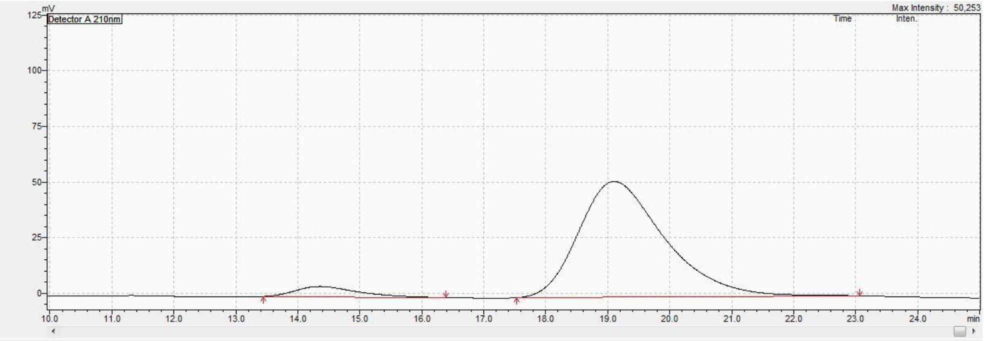

Results View - Peak Table

Peak Table | Compound | Group | Calibration Curve

Peak#	Ret. Time	Area	Height	Mark	Conc.	Unit	ID#	Name	Area%
1	14,356	323928	4644	M	6,066				6,066
2	19,105	5015881	52038	M	93,934				93,934
Total		5339809	56682		100,000				100,000

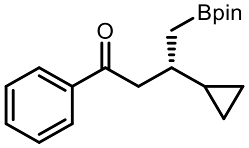

(*S*)-3-cyclopropyl-1-phenyl-4-(4,4,5,5-tetramethyl-1,3,2-dioxaborolan-2-yl)butan-1-one (3o) Following general procedure stirring for 36 hours instead of 11 hours, the crude product was purified by silica gel chromatography (hexanes : DCM = 50 : 50 → 0 : 100, DCM : MeOH = 100 : 0 → 99.5 : 0.5) to provide **3o** as a colorless liquid (66 mg, 70%, 87% ee).

^{1}H NMR (400 MHz, CDCl$_3$) δ 8.03–7.95 (m, 2H), 7.56–7.49 (m, 1H), 7.47–7.40 (m, 2H), 3.16 (dd, J = 14.9, 6.9 Hz, 1H), 2.98 (dd, J = 14.9, 6.8 Hz, 1H), 1.61–1.48 (m, 1H), 1.24 (s, 12H), 1.06–0.93 (m, 2H), 0.78–0.66 (m, 1H), 0.43–0.28 (m, 2H), 0.20–0.11 (m, 1H), 0.05–0.01 (m, 1H) ppm.

^{13}C NMR (101 MHz, CDCl$_3$) δ 200.62, 137.61, 132.61, 128.39, 128.29, 82.99, 46.29, 37.60, 24.94, 24.85, 18.60, 4.86, 4.27 ppm.

^{11}B NMR (128 MHz, CDCl$_3$) δ 33.25 ppm.

HRMS (ESI) calculated for $[C_{19}H_{27}BO_3+H]^+$: 315.2126, found: 315.2135.

Optical rotation, $[\alpha]_D^{22}$ = –11.6 (c = 1.11, DCM).

Enantiomeric excess, 87% ee was measured by HPLC (CHIRALCEL OD-H, *n*-hexane : *i*-PrOH = 99.7 : 0.3, 1.0 mL/min, wavelength = 254 nm, 28 °C); t_R = 11.49 min (major), t_R = 9.68 min (minor).

[Racemic **3o**]

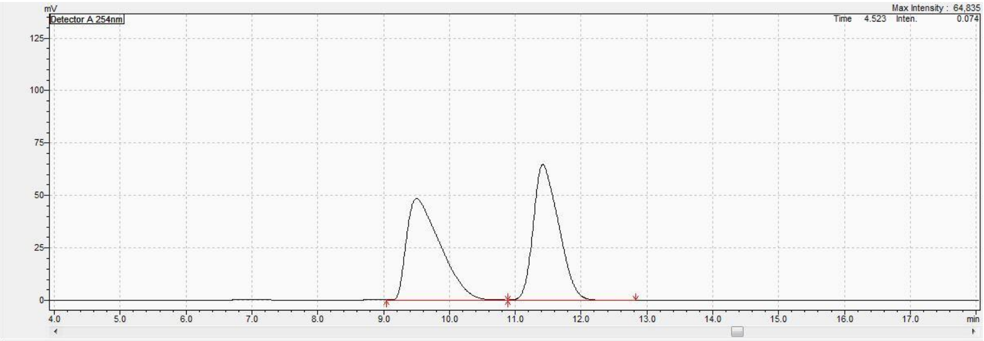

Results View - Peak Table

Peak Table | Compound | Group | Calibration Curve

Peak#	Ret. Time	Area	Height	Mark	Conc.	Unit	ID#	Name	Area%
1	9.502	1728707	48443		50.019				50.019
2	11.417	1727404	64767	V	49.981				49.981
Total		3456110	113211		100.000				100.000

[Enantioenriched **3o**]

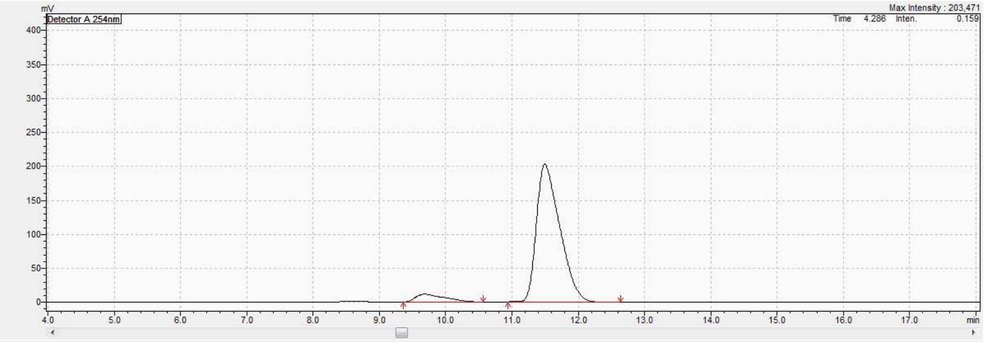

Results View - Peak Table

Peak Table | Compound | Group | Calibration Curve

Peak#	Ret. Time	Area	Height	Mark	Conc.	Unit	ID#	Name	Area%
1	9.683	354657	11515	M	6.679				6.679
2	11.495	4955563	202845	M	93.321				93.321
Total		5310219	214360		100.000				100.000

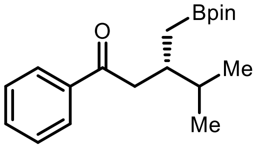

(*S*)-4-methyl-1-phenyl-3-((4,4,5,5-tetramethyl-1,3,2-dioxaborolan-2-yl)methyl) pentan-1-one (3p) Following general procedure stirring for 36 hours instead of 11 hours, the crude product was purified by silica gel chromatography (hexanes : DCM = 50 : 50 → 0 : 100, DCM : MeOH = 100 : 0 → 99.5 : 0.5) to provide **3p** as a yellowish liquid (64 mg, 68%, 85% ee).

^{1}H NMR (400 MHz, CDCl$_3$) δ 8.02–7.98 (m, 2H), 7.56–7.50 (m, 1H), 7.48–7.40 (m, 2H), 2.97 (dd, *J* = 15.6, 7.1 Hz, 1H), 2.86 (dd, *J* = 15.6, 6.5 Hz, 1H), 2.33–2.23 (m, 1H), 1.78–1.64 (m, 1H), 1.22 (s, 6H), 1.21 (s, 6H), 0.88 (m, 1H), 0.87 (dd, *J* = 6.8, 1.7 Hz, 6H), 0.75 (dd, *J* = 15.7, 8.5 Hz, 1H) ppm.

^{13}C NMR (101 MHz, CDCl$_3$) δ 200.82, 137.50, 132.60, 128.37, 128.26, 82.94, 42.67, 36.60, 31.84, 24.85, 24.81, 19.69, 18.36 ppm.

^{11}B NMR (128 MHz, CDCl$_3$) δ 34.20 ppm.

HRMS (ESI) calculated for $[C_{19}H_{29}BO_3+H]^+$: 317.2282, found: 317.2292.

Optical rotation, $[\alpha]_D^{22}$ = –10.6 (c = 1.06, DCM).

Enantiomeric excess, 85% ee was measured by HPLC (CHIRALCEL OD-H, *n*-hexane : *i*-PrOH = 99.3 : 0.7, 1.0 mL/min, wavelength = 254 nm, 28 °C); t_R = 12.40 min (major), t_R = 10.38 min (minor).

[Racemic **3p**]

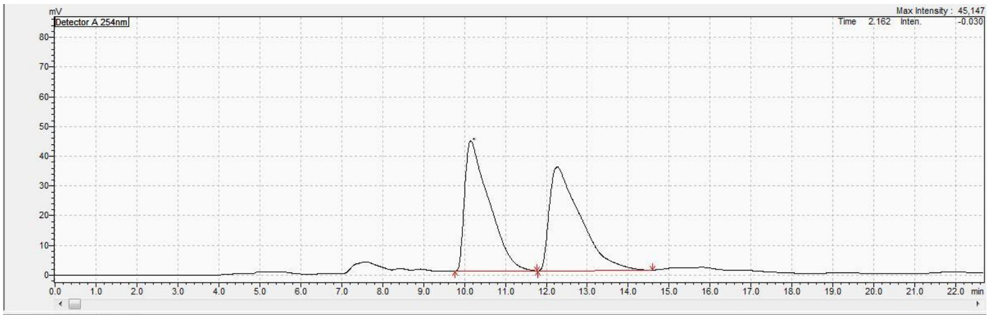

Results View - Peak Table

Peak Table | Compound | Group | Calibration Curve

Peak#	Ret. Time	Area	Height	Mark	Conc.	Unit	ID#	Name	Area%
1	10,155	1811670	43720	M	49,770				49,770
2	12,263	1828428	34926	M	50,230				50,230
Total		3640097	78646		100,000				100,000

[Enantioenriched **3p**]

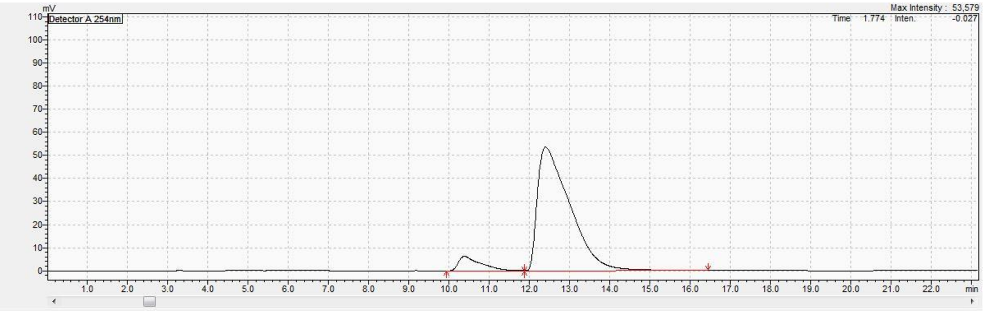

Results View - Peak Table

Peak Table | Compound | Group | Calibration Curve

Peak#	Ret. Time	Area	Height	Mark	Conc.	Unit	ID#	Name	Area%
1	10,382	242171	6266		7,447				7,447
2	12,403	3009742	53559	V	92,553				92,553
Total		3251913	59825		100,000				100,000

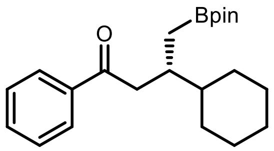

(*S*)-3-cyclohexyl-1-phenyl-4-(4,4,5,5-tetramethyl-1,3,2-dioxaborolan-2-yl)butan-1-one (3q) Following general procedure stirring for 36 hours instead of 11 hours, the crude product was purified by silica gel chromatography (hexanes : DCM = 50 : 50 → 0 : 100, DCM : MeOH = 100 : 0 → 99.5 : 0.5) to provide **3q** as a colorless liquid (77 mg, 72%, 80% ee).

^{1}H NMR (400 MHz, $CDCl_3$) δ 8.03–7.96 (m, 2H), 7.56–7.49 (m, 1H), 7.47–7.40 (m, 2H), 2.94 (d, *J* = 6.8 Hz, 2H), 2.31–2.21 (m, 1H), 1.78–1.57 (m, 5H), 1.36–1.27 (m, 1H), 1.21 (s, 6H), 1.21 (s, 6H), 1.18 – 0.95 (m, 5H), 0.89 (dd, *J* = 15.7, 5.9 Hz, 1H), 0.79 (dd, *J* = 15.7, 8.1 Hz, 1H) ppm.

^{13}C NMR (101 MHz, $CDCl_3$) δ 200.85, 137.52, 132.58, 128.36, 128.24, 82.90, 42.77, 42.69, 36.10, 30.11, 29.31, 26.75, 26.69, 26.68, 24.82, 24.79 ppm. The carbon bound to the boron was not detected due to quadrupolar relaxation.

^{11}B NMR (128 MHz, $CDCl_3$) δ 33.29 ppm.

HRMS (ESI) calculated for $[C_{22}H_{33}BO_3+H]^+$: 357.2595, found: 357.2610.

Optical rotation, $[\alpha]_D^{22}$ = –9.4 (c = 0.8, DCM).

Enantiomeric excess, 80% ee was measured by HPLC (CHIRALCEL OJ-H, *n*-hexane : *i*-PrOH = 99.8 : 0.2), 0.2 mL/min, wavelength = 210 nm, 30 °C); t_R = 34.10 min (major), t_R = 27.73 min (minor).

[Racemic **3q**]

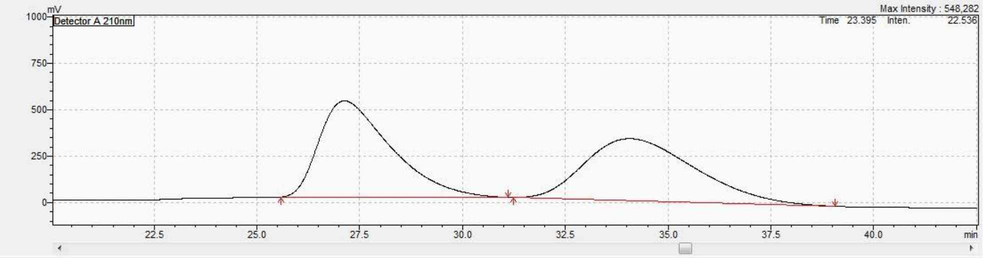

Results View - Peak Table

Peak Table | Compound | Group | Calibration Curve

Peak#	Ret. Time	Area	Height	Mark	Conc.	Unit	ID#	Name	Area%
1	27,129	63140802	519389	M	50,214				50,214
2	34,061	62602764	334409	M	49,786				49,786
Total		125743566	853799		100,000				100,000

[Enantioenriched **3q**]

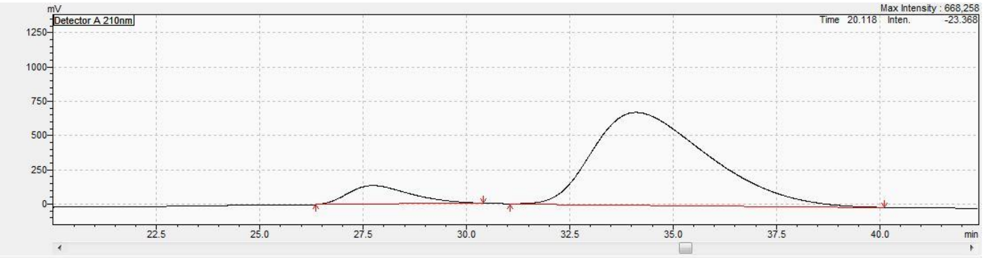

Results View - Peak Table

Peak Table | Compound | Group | Calibration Curve

Peak#	Ret. Time	Area	Height	Mark	Conc.	Unit	ID#	Name	Area%
1	27,735	14258511	135205	M	9,789				9,789
2	34,105	131395458	675339	M	90,211				90,211
Total		145653968	810543		100,000				100,000

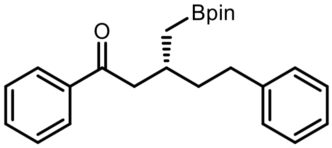

(*R*)-1,5-diphenyl-3-((4,4,5,5-tetramethyl-1,3,2-dioxaborolan-2-yl)methyl)pentan-1-one (3r) Following general procedure stirring for 36 hours instead of 11 hours, the crude product was purified by silica gel chromatography (hexanes : DCM = 50 : 50 → 0 : 100, DCM : MeOH = 100 : 0 → 99.5 : 0.5) to provide **3r** as a yellowish liquid (51 mg, 45%, 81% ee).

^{11}H NMR (400 MHz, $CDCl_3$) δ 8.05–7.93 (m, 2H), 7.58–7.50 (m, 1H), 7.49–7.41 (m, 2H), 7.29–7.21 (m, 2H), 7.20–7.12 (m, 3H), 3.09 (dd, *J* = 15.6, 6.6 Hz, 1H), 2.93 (dd, *J* = 15.6, 7.0 Hz, 1H), 2.75–2.55 (m, 2H), 2.48–2.37 (m, 1H), 1.81–1.59 (m, 2H), 1.26 (s, 12H), 1.02 (dd, *J* = 15.8, 6.1 Hz, 1H), 0.95 (dd, *J* = 15.9, 7.2 Hz, 1H) ppm.

^{13}C NMR (101 MHz, $CDCl_3$) δ 200.40, 142.61, 137.43, 132.72, 128.43, 128.30, 128.23 (2C), 125.56, 83.04, 45.42, 38.60, 33.28, 31.16, 24.88, 24.87 ppm. The carbon bound to the boron was not detected due to quadrupolar relaxation.

^{11}B NMR (128 MHz, $CDCl_3$) δ 33.49 ppm.

HRMS (ESI) calculated for $[C_{24}H_{31}BO_3+H]^+$: 379.2439, found: 379.2448.

Optical rotation, $[\alpha]_D^{22}$ = +5.3 (c = 0.28, DCM).

Enantiomeric excess, 81% ee was measured by HPLC (CHIRALPAK AD-H, *n*-hexane : *i*-PrOH = 98 : 2, 0.2 mL/min, wavelength = 210 nm, 30 ℃); t_R = 61.57 min (major), t_R = 62.52 min (minor).

[Racemic **3r**]

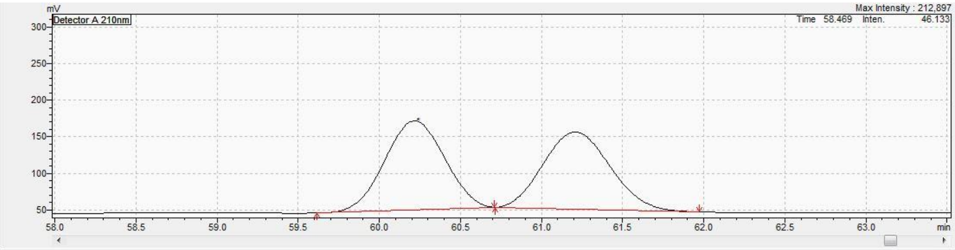

Results View - Peak Table

Peak Table | Compound | Group | Calibration Curve

Peak#	Ret. Time	Area	Height	Mark	Conc.	Unit	ID#	Name	Area%
1	60.218	3109517	121384	M	50.763				50.763
2	61.207	3016069	105098	M	49.237				49.237
Total		6125586	226482		100.000				100.000

[Enantioenriched **3r**]

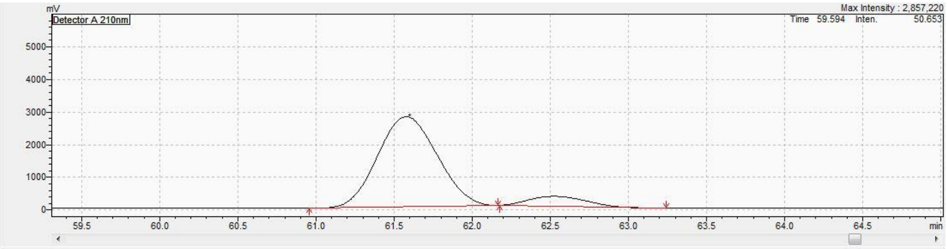

Results View - Peak Table

Peak Table | Compound | Group | Calibration Curve

Peak#	Ret. Time	Area	Height	Mark	Conc.	Unit	ID#	Name	Area%
1	61.578	73326762	2764072	M	90.446				90.446
2	62.528	7745721	307582	M	9.554				9.554
Total		81072483	3071654		100.000				100.000

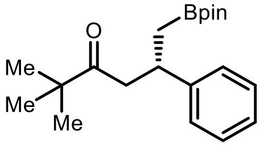

(*S*)-2,2-dimethyl-5-phenyl-6-(4,4,5,5-tetramethyl-1,3,2-dioxaborolan-2-yl)hexan-3-one (3s) Following general procedure stirring for 36 hours instead of 11 hours, the crude product was purified by silica gel chromatography (hexanes : DCM = 100 : 0 → 0 : 100) to provide **3s** as a yellowish liquid (61 mg, 62%, 52% ee).

^{1}H NMR (500 MHz, $CDCl_3$) δ 7.24–7.18 (m, 4H), 7.16–7.10 (m, 1H), 3.52–3.43 (m, 1H), 2.81 (dd, *J* = 15.6, 5.6 Hz, 1H), 2.77 (dd, *J* = 15.7, 5.7 Hz, 1H), 1.19 (dd, *J* = 15.3, 6.5 Hz, 1H), 1.14–1.10 (m, 1H), 1.08 (s, 6H), 1.06 (s, 6H), 1.01 (s, 9H) ppm.

^{13}C NMR (126 MHz, $CDCl_3$) δ 214.07, 146.69, 128.04, 127.33, 125.89, 82.91, 45.82, 43.96, 36.43, 26.03, 24.66, 24.57 ppm. The carbon bound to the boron was not detected due to quadrupolar relaxation.

^{11}B NMR (128 MHz, $CDCl_3$) δ 32.40 ppm.

HRMS (ESI) calculated for $[C_{20}H_{31}BO_3+H]^+$: 331.2439, found: 331.2441.

Optical rotation, $[\alpha]_D^{22}$ = +6.61 (c = 1.50, DCM).

Enantiomeric excess, 52% ee was measured by HPLC (CHIRALPAK IA, *n*-hexane : *i*-PrOH = 99 : 1, 1.0 mL/min, wavelength = 254 nm, 28 ℃); t_R = 4.97 min (major), t_R = 4.55 min (minor).

[Racemic product **3s**]

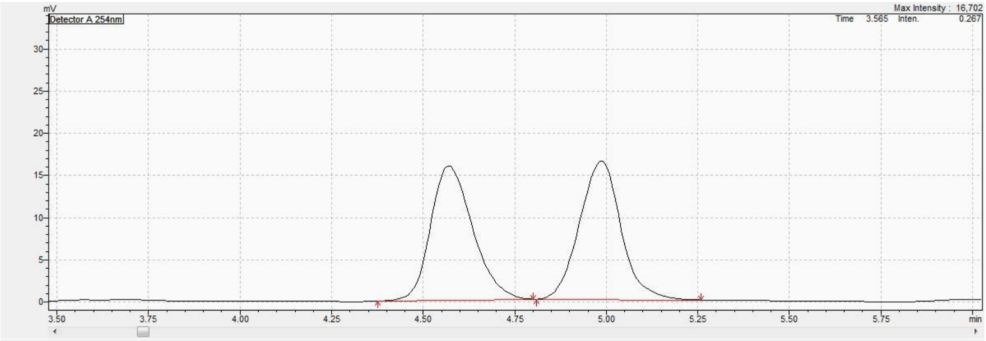

Results View - Peak Table

Peak Table | Compound | Group | Calibration Curve

Peak#	Ret. Time	Area	Height	Mark	Conc.	Unit	ID#	Name	Area%
1	4.570	127297	15923	M	49.694				49.694
2	4.987	128865	16390	M	50.306				50.306
Total		256162	32312		100.000				100.000

[Enantioenriched **3s**]

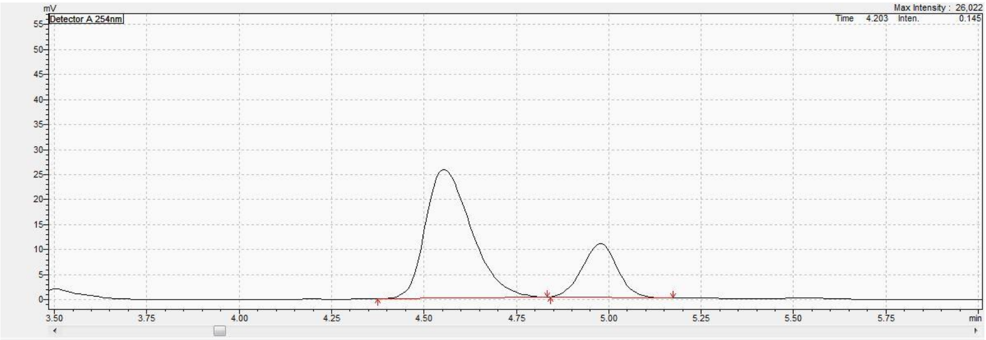

Results View - Peak Table

Peak Table | Compound | Group | Calibration Curve

Peak#	Ret. Time	Area	Height	Mark	Conc.	Unit	ID#	Name	Area%
1	4.554	222517	25690	M	76.099				76.099
2	4.978	69888	10693	M	23.901				23.901
Total		292405	36383		100.000				100.000

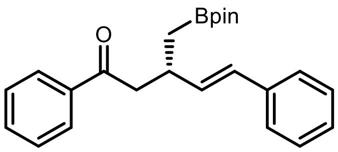

(*R*)-1,5-diphenyl-3-((4,4,5,5-tetramethyl-1,3,2-dioxaborolan-2-yl)methyl)pentan-1-one (3t) Following general procedure stirring for 36 hours instead of 11 hours, the crude product was purified by silica gel chromatography (hexanes : DCM = 50 : 50 → 0 : 100, DCM : MeOH = 100 : 0 → 99.5 : 0.5) to provide **3t** as a yellowish solid (54 mg, 48%, 75% ee).

^{1}H NMR (400 MHz, $CDCl_3$) δ 8.01–7.92 (m, 2H), 7.57–7.49 (m, 1H), 7.47–7.39 (t, *J* = 7.6 Hz, 2H), 7.30– 7.20 (m, 4H), 7.18–7.11 (m, 1H), 6.37 (d, *J* = 15.9 Hz, 1H), 6.20 (dd, *J* = 15.9, 7.5 Hz, 1H), 3.23–3.12 (m, 2H), 3.10–3.00 (m, 1H), 1.20 (s, 12H), 1.12 (dd, *J* = 15.6, 6.0 Hz, 1H), 1.05 (dd, *J* = 15.7, 7.5 Hz, 1H) ppm.

^{13}C NMR (101 MHz, $CDCl_3$) δ 199.35, 137.55, 137.36, 134.81, 132.80, 128.85, 128.48, 128.35, 128.22, 126.87, 126.10, 83.15, 46.00, 35.11, 24.94, 24.79 ppm. The carbon bound to the boron was not detected due to quadrupolar relaxation.

^{11}B NMR (128 MHz, $CDCl_3$) δ 33.07 ppm.

HRMS (ESI) calculated for $[C_{24}H_{29}BO_3+H]^+$: 377.2282, found: 377.2279.

Optical rotation, $[\alpha]_D^{22}$ = +12.9 (c = 0.88, DCM).

Enantiomeric excess, 75% ee was measured by HPLC (CHIRALPAK IA, *n*-hexane : *i*-PrOH = 99.8 : 0.2, 0.6 mL/min, wavelength = 210 nm, 30 ℃); t_R = 18.04 min (major), t_R = 24.08 min (minor).

[Racemic product **3t**]

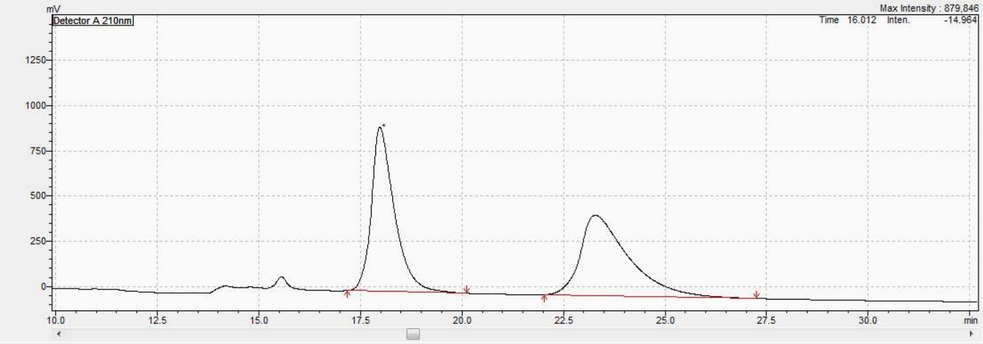

Results View - Peak Table

Peak Table | Compound | Group | Calibration Curve

Peak#	Ret. Time	Area	Height	Mark	Conc.	Unit	ID#	Name	Area%
1	17.979	35858624	906512	M	49.426				49.426
2	23.280	36692134	444103	M	50.574				50.574
Total		72550759	1350615		100.000				100.000

[Enantioenriched **3t**]

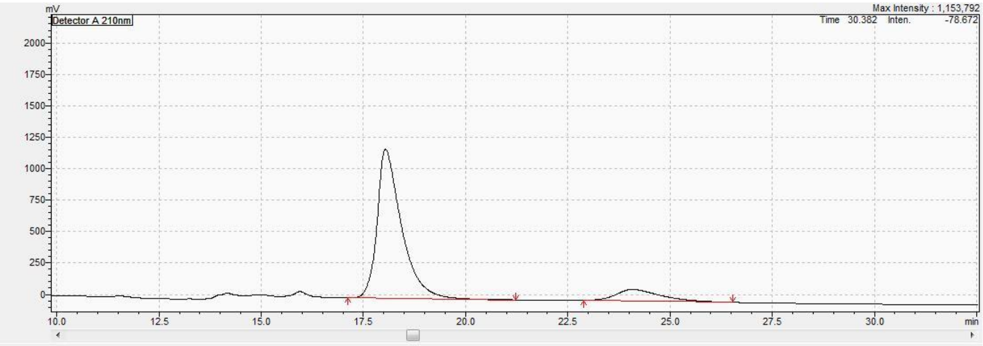

Results View - Peak Table

Peak Table | Compound | Group | Calibration Curve

Peak#	Ret. Time	Area	Height	Mark	Conc.	Unit	ID#	Name	Area%
1	18.041	49146330	1183510	M	87.441				87.441
2	24.085	7058483	91853	M	12.559				12.559
Total		56204813	1275363		100.000				100.000

5. General procedure for further derivatizations of (*S*)-3a (Scheme 3)

5.1. Palladium-catalysed cross coupling

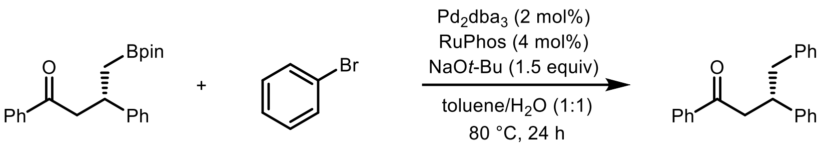

(*S*)-**3a**
92% ee

1.5 equiv

3ab
72% yield, 91% ee

(*S*)-(–)-1,3,4-triphenylbutan-1-one (3ab).[4]

In a nitrogen-filled glove box, an oven-dried 4 mL dram-vial was charged with a magnetic stir bar, (*S*)-**3a** (92% ee, 0.47 mmol, 1.0 equiv, 164 mg), Pd_2dba_3 (0.009 mmol, 2 mol%, 8.7 mg), RuPhos (0.019 mmol, 4 mol%, 8.8 mg), NaO*t*-Bu (1.9 mmol, 4.0 equiv, 179 mg), and bromobenzene (0.71 mmol, 1.5 equiv, 73 µL). Subsequently, anhydrous toluene (1.6 mL) was added to the mixture via a syringe and the system was sealed with an open-top teflon faced screw cap. The vial was removed from the glovebox and then, H_2O (0.16 mL) was added. The reaction mixture was vigorously stirred for 24 hours at 80 °C. Upon completion of the reaction, the reaction mixture was cooled to room temperature and brine (20 mL) was added in one portion to quench the reaction. The aqueous phase was separated and extracted with ethyl acetate (3 × 20 mL). The combined organic phase was dried over anhydrous sodium sulfate (Na_2SO_4), filtered, and concentrated under reduced pressure. The crude product was purified by flash column chromatography on silica gel (hexanes : EtOAc = 100 : 0 → hexanes : EtOAc = 97 : 3) to provide **3ab** (95 mg, 72%, 91% ee). **^{1}H NMR** (400 MHz, $CDCl_3$) δ 7.90–7.82 (m, 2H), 7.56–7.49 (m, 1H), 7.46–7.37 (m, 2H), 7.31–7.13 (m, 8H), 7.12–7.05 (m, 2H), 3.74–3.63 (m, 1H), 3.36 (dd, *J* = 16.8, 7.3 Hz, 1H), 3.29 (dd, *J* = 16.8, 6.6 Hz, 1H), δ 3.02 (dd, *J* = 13.9, 7.6 Hz, 1H), 2.97 (dd, *J* = 13.7, 8.0 Hz, 1H).**^{13}C NMR** (101 MHz, $CDCl_3$) δ 197.53, 173.79, 138.36, 136.35, 133.29, 128.90, 128.58, 128.05, 127.81, 127.55, 52.26, 46.34, 42.78 ppm. **Enantiomeric excess**, 91% ee was measured by HPLC (CHIRALPAK IA, *n*-hexane : *i*-PrOH = 98.5 : 1.5 → 98:2, 1.0 mL/min, wavelength = 210 nm, 30 °C); t_R = 8.51 min (major), t_R = 11.04 min (minor); $[\alpha]_D^{22}$ = –5.5 (c = 0.72, DCM).

[Racemic **3ab**]

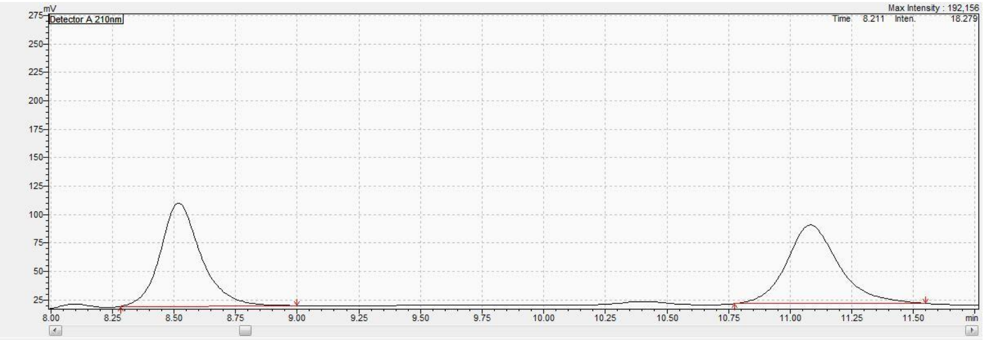

Results View - Peak Table

Peak Table | Compound | Group | Calibration Curve

Peak#	Ret. Time	Area	Height	Mark	Conc.	Unit	ID#	Name	Area%
1	8.519	1029393	90730	M	50.280				50.280
2	11.083	1017913	69272	M	49.720				49.720
Total		2047306	160001		100.000				100.000

[Enantioenriched **3ab**]

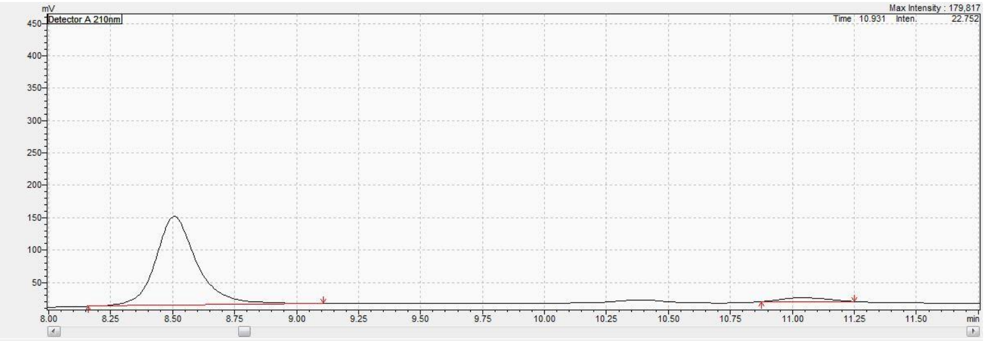

Results View - Peak Table

Peak Table | Compound | Group | Calibration Curve

Peak#	Ret. Time	Area	Height	Mark	Conc.	Unit	ID#	Name	Area%
1	8.506	1605953	137374	M	95.385				95.385
2	11.040	77697	5979	M	4.615				4.615
Total		1683651	143353		100.000				100.000

5.2. Oxidation and esterification.

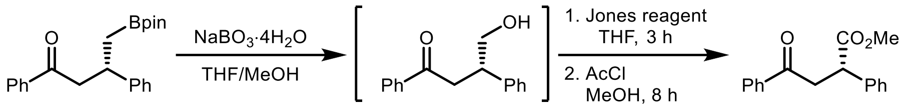

(*S*)-**3a**
92% ee

3ac

3ad
51% yield, 93% ee

Methyl (*S*)-4-oxo-2,4-diphenylbutanoate (3ad).[5]

A 4 mL dram-vial containing a magnetic stir bar was charged with (*S*)-**3a** (92% ee, 0.37 mmol, 1.0 equiv, 128 mg) and $NaBO_3 \cdot 4H_2O$ (0.74 mmol, 2.0 equiv, 114 mg). Subsequently, 1.6 mL of THF/MeOH mixture (v/v = 1:1) were added to the mixture via a syringe. The reaction mixture was vigorously stirred for 4 hours. Upon completion of the reaction, brine (10 mL) was added in one portion to quench the reaction. The aqueous phase was separated and extracted with ethyl acetate (3 × 10 mL). The combined organic phase was dried over anhydrous sodium sulfate (Na_2SO_4), filtered, and concentrated under reduced pressure. Subsequently, the crude product was dissolved in THF (0.8 mL) and then, 2.5M Jones reagent (CrO_3/H_2SO_4, 0.56 mmol, 1.5 equiv, 0.23 mL) was added. After stirring 3 hours, brine (10 mL) was added in one portion to quench the reaction. The aqueous phase was separated and extracted with ethyl acetate (3 × 10 mL). The combined organic phase was dried over anhydrous sodium sulfate (Na_2SO_4), filtered, and concentrated *in vacuo*. The crude product was dissolved in MeOH (1 mL) and acetyl chloride (AcCl) (0.74 mmol, 2.0 equiv, 52 μL) was added to the reaction mixture. After stirring for 8 hours, the reaction mixture was diluted with brine (10 mL), extracted with ethyl acetate (3 × 20 mL). The organic layer was dried over anhydrous sodium sulfate (Na_2SO_4), and concentrated under reduced pressure. The resulting residue was purified by flash column chromatography (hexanes : EtOAc = 100 : 0 → hexanes : EtOAc = 75 : 25) to provide **3ad** (51 mg, 51%, 93% ee). **^{1}H NMR** (400 MHz, $CDCl_3$) δ 8.02–7.93 (m, 2H), 7.60–7.53 (m, 1H), 7.50–7.42 (m, 2H), 7.41–7.26 (m, 5H), 4.31 (dd, J = 10.3, 4.1 Hz, 1H), 3.96 (dd, J = 18.0, 10.3 Hz, 1H), 3.70 (s, 3H), 3.28 (dd, J = 18.0, 4.1 Hz, 1H) ppm. **^{13}C NMR** (101 MHz, $CDCl_3$) δ 197.58, 173.81, 138.31, 136.35, 133.28, 128.88, 128.56, 128.05, 127.79, 127.52, 52.31, 46.32, 42.77 ppm. **Enantiomeric excess**, 93% ee was measured by HPLC (CHIRALCEL OD-H, *n*-hexane : *i*-PrOH = 98.3 : 1.7, 1.0 mL/min, wavelength = 254 nm, 28 °C); t_R = 17.22 min (major), t_R = 15.75 min (minor); $[\alpha]_D^{22}$ = +84.4 (c = 0.73, DCM).

[Racemic **3ad**]

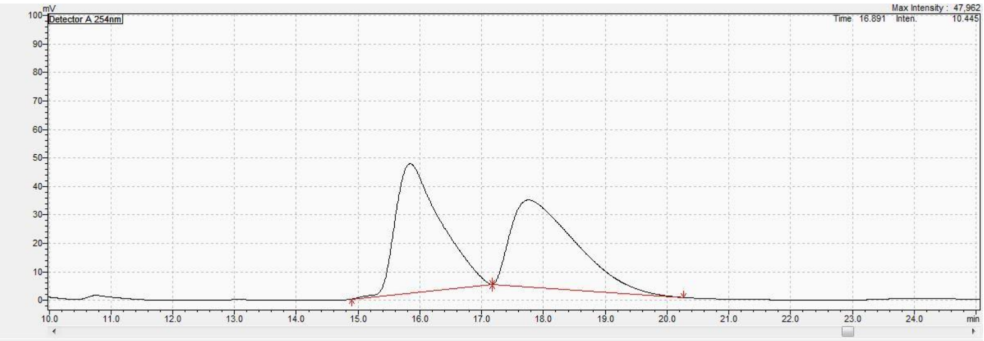

Results View - Peak Table

Peak Table | Compound | Group | Calibration Curve

Peak#	Ret. Time	Area	Height	Mark	Conc.	Unit	ID#	Name	Area%
1	15.842	2303350	45360	M	50.878				50.878
2	17.758	2223859	30513	M	49.122				49.122
Total		4527209	75873		100.000				100.000

[Enantioenriched **3ad**]

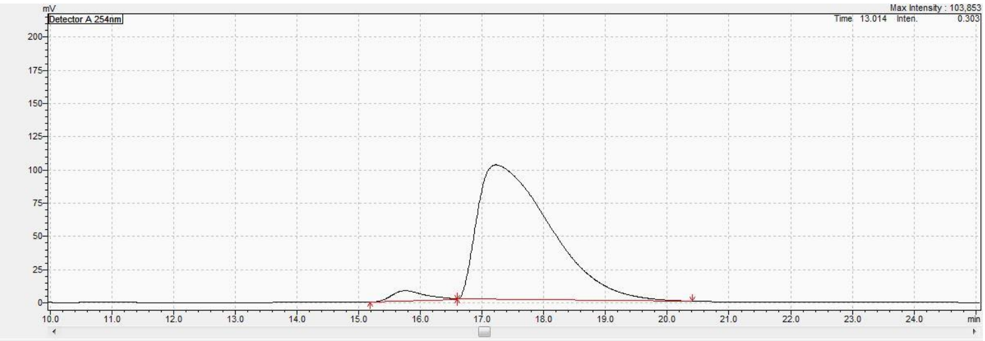

Results View - Peak Table

Peak Table | Compound | Group | Calibration Curve

Peak#	Ret. Time	Area	Height	Mark	Conc.	Unit	ID#	Name	Area%
1	15.757	296179	7770	M	3.459				3.459
2	17.227	8265183	101090	M	96.541				96.541
Total		8561362	108860		100.000				100.000

5.3. Oxidation, reduction and acid-catalysed ring formation.

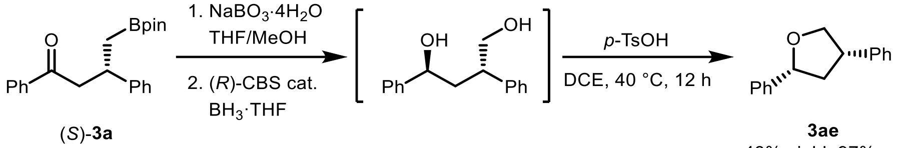

(*S*)-**3a**
92% ee

3ae
48% yield, 97% ee
(d.r = 5:1)

(2*R*,4*S*)-2,4-diphenyltetrahydrofuran (*ent*-Calyxolane B, 3ae).[6]

A 4 mL dram-vial containing a magnetic stir bar was charged with (*S*)-**3a** (92% ee, 0.37 mmol, 1.0 equiv, 128 mg), $NaBO_3·4H_2O$ (0.74 mmol, 2.0 equiv, 114 mg). Subsequently, 1.6 mL of THF/MeOH mixture (v/v = 1:1) ware added to the mixture via a syringe. The reaction mixture was vigorously stirred for 4 hours. Upon completion of the reaction, brine (10 mL) was added in one portion to quench the reaction. The aqueous phase was separated and extracted with ethyl acetate (3 × 10 mL). The combined organic phase was dried over anhydrous sodium sulfate (Na_2SO_4), filtered, and concentrated under reduced pressure. The resulting mixture was dissolved in THF (0.3 mL). To an another 4 ml vial containing a magnetic stir bar were added (*R*)-CBS catalyst (0.08 mmol, 20 mol%, 22 mg) and anhydrous THF (0.5 mL). Subsequently, $BH_3·THF$ solution (1.0 M in THF, 0.74 mmol, 2.0 equiv, 0.74 mL) solution was added to the reaction mixture at 0 °C. After stirring for 30 mins, the crude mixture was added to the vial containing pre-activated (*R*)-CBS catalyst solution. The system was vigorously stirred for 8 hours at room temperature. Upon completion of the reaction, brine (10 mL) was added in one portion to quench the reaction. The aqueous phase was separated and extracted with ethyl acetate (3 × 10 mL). The combined organic phase was dried over anhydrous sodium sulfate (Na_2SO_4), filtered, and concentrated under reduced pressure. The crude product (diol) and PTSA (0.04 mmol, 10 mol%, 6.9 mg) was dissolved in 1,2-DCE (1 mL) and stirred for 12 hours at 40 °C. Upon completion of the reaction, brine (10 mL) was added to quench the reaction. The aqueous phase was separated and extracted with ethyl acetate (3 × 10 mL). The organic layer, which was dried by sodium sulfate Na_2SO_4, was concentrated *in vacuo*. The concentrated crude product was purified by flash column chromatography (Petroleum ether : EtOAc = 100 : 0 → Petroleum ether : EtOAc = 98 : 2) to provide **3ae** (40 mg, 48%, 97% ee). The *rac*-**3ae** was synthesized using *rac*-**3a**; ketone reduction was performed using $NaBH_4$ (0.6 mmol, 200 mol%, 23mg) instead of (*R*)-CBS catalyst. **^{1}H NMR** (400 MHz, $CDCl_3$) δ 7.46–7.17 (m, 10H), 5.08 (dd, *J* = 10.2, 5.7 Hz, 1H), 4.37 (t, *J* = 8.3 Hz, 1H), 4.03 (t, *J* = 8.5 Hz, 1H), 3.75–3.58 (m, 1H), 2.84–2.74 (m, 1H), 2.11–1.98 (m, 1H) ppm. **^{13}C NMR** (101 MHz, $CDCl_3$) δ 142.65, 141.70, 128.61, 128.43, 127.40, 127.25, 126.65, 125.71, 81.84, 75.11, 46.03, 43.73 ppm. **Enantiomeric excess**, 97% ee was measured by HPLC (CHIRALCEL OD, *n*-hexane : *i*-PrOH = 90 : 10, 1.0 mL/min, wavelength = 210 nm, 30 °C); t_R = 5.80 min (major), t_R = 7.08 min (minor); $[\alpha]_D^{22}$ = +46.4 (c = 0.61, DCM).

[Racemic product of **3ae**]

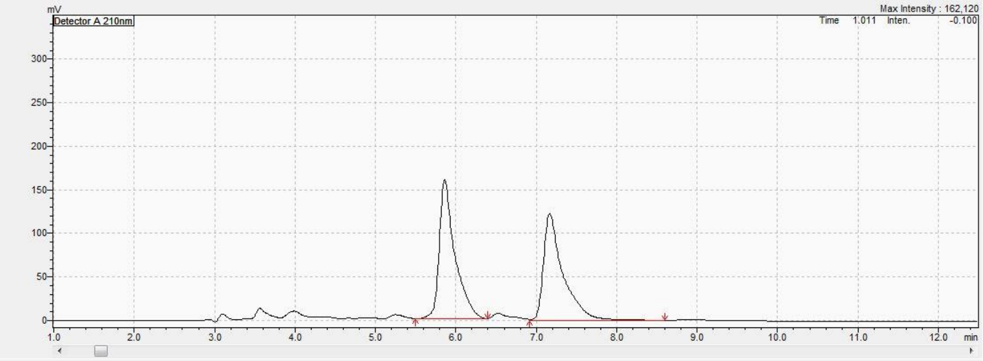

Results View - Peak Table

Peak Table | Compound | Group | Calibration Curve

Peak#	Ret. Time	Area	Height	Mark	Conc.	Unit	ID#	Name	Area%
1	5.861	2158746	159903	M	50.715				50.715
2	7.169	2097882	123270	M	49.285				49.285
Total		4256628	283172		100.000				100.000

[Enantioenriched product of **3ae**]

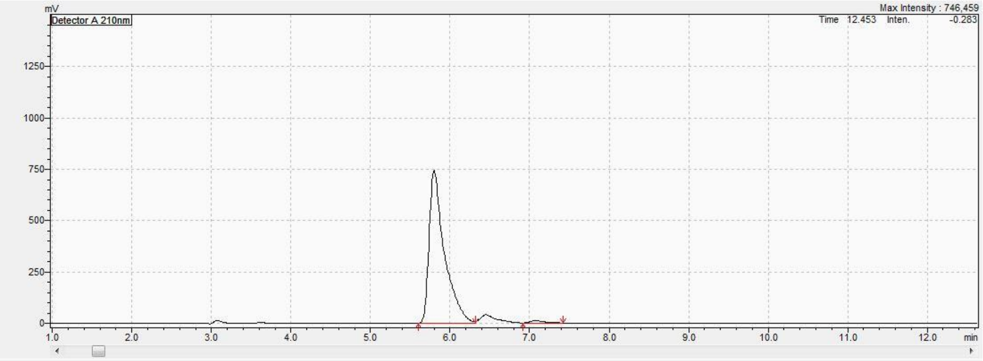

Results View - Peak Table

Peak Table | Compound | Group | Calibration Curve

Peak#	Ret. Time	Area	Height	Mark	Conc.	Unit	ID#	Name	Area%
1	5.800	9931272	745974	M	98.671				98.671
2	7.078	133792	10658	M	1.329				1.329
Total		10065064	756632		100.000				100.000

6. General procedure for ^{31}P, ^{11}B NMR experiments

6.1. In situ monitoring of reaction system by ^{31}P NMR spectroscopies

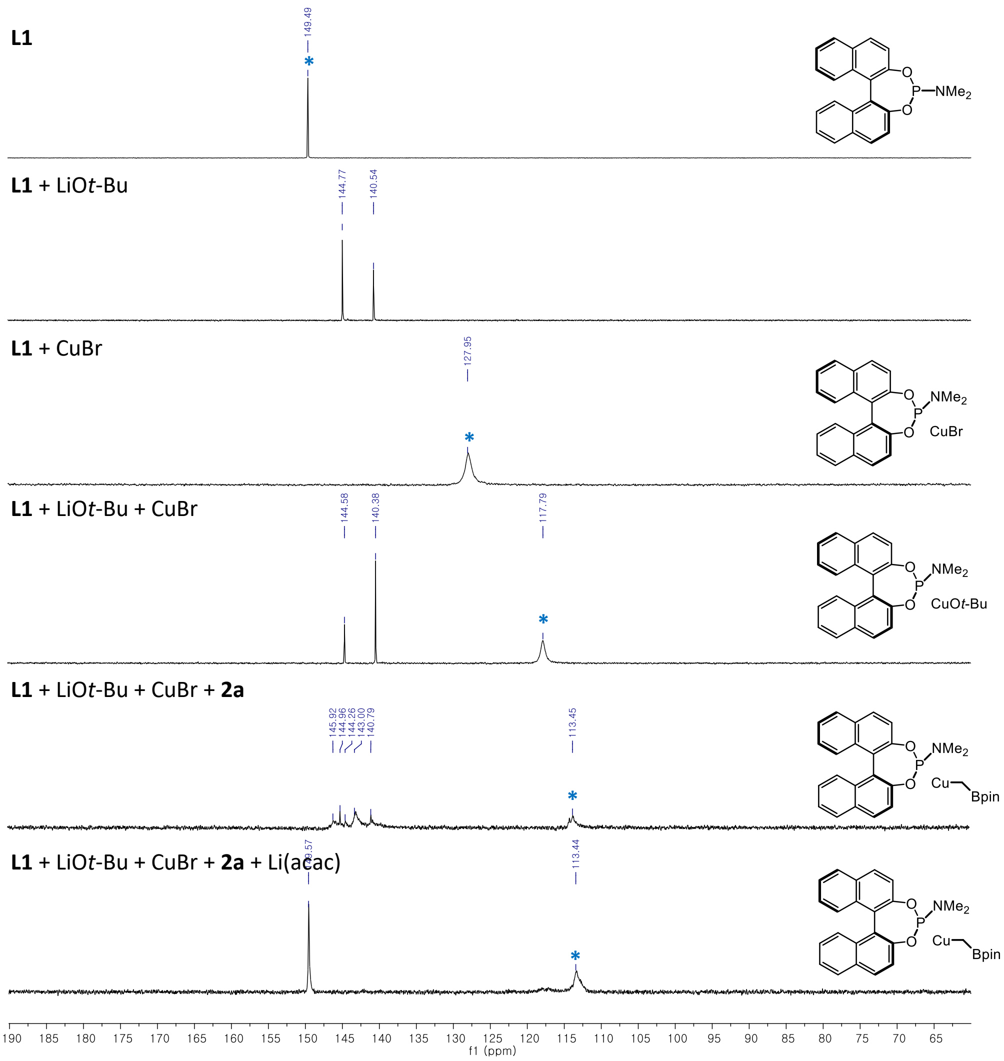

Figure S1. ^{31}P NMR spectra (162 MHz, THF-d_8, rt) of the reaction mixture

6.2. Monitoring of ligand decomposition over time by ^{31}P NMR spectroscopies

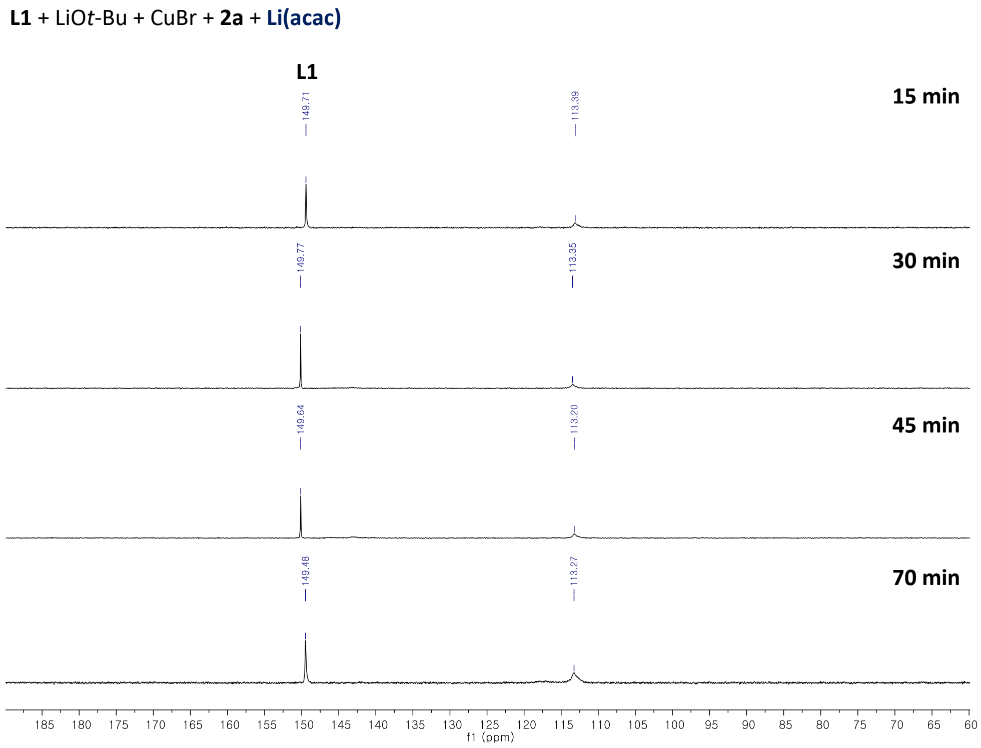

Figure S2. ^{31}P NMR spectra (162 MHz, THF-d_8, rt) of the reaction mixture in the presence of Li(acac).

L1 + LiO*t*-Bu + CuBr + **2a**

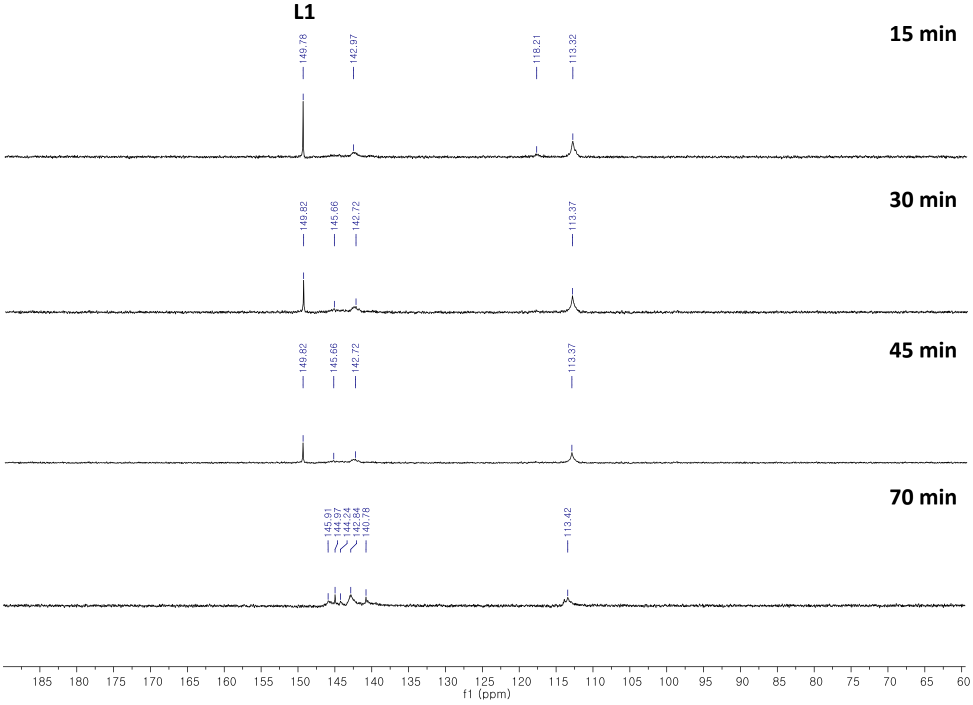

Figure S3. ^{31}P NMR spectra (162 MHz, THF-d_8, rt) of the reaction mixture in the absence of Li(acac)

6.3. A related phosphoramidite-based copper-alkyl species.[7]

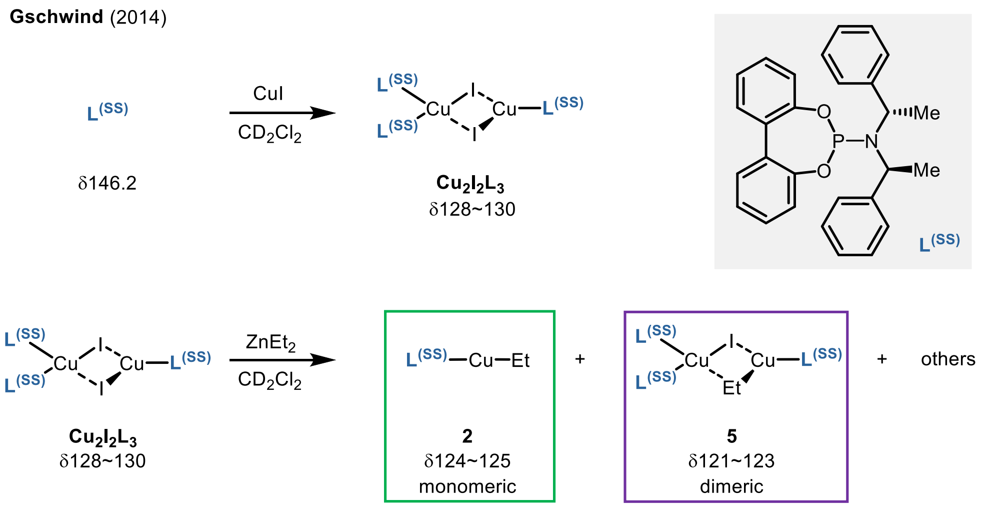

6.4. Studies to elucidate the role of Li(acac).

■ Viability of Li(acac) as a better activator than LiO*t*-Bu

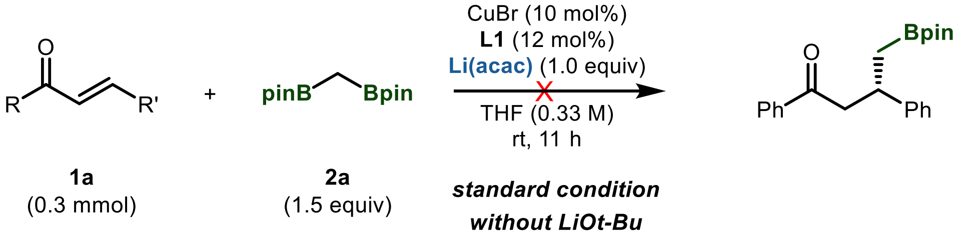

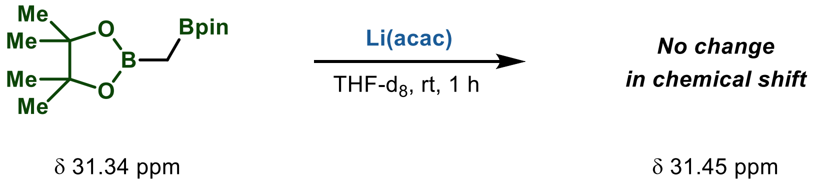

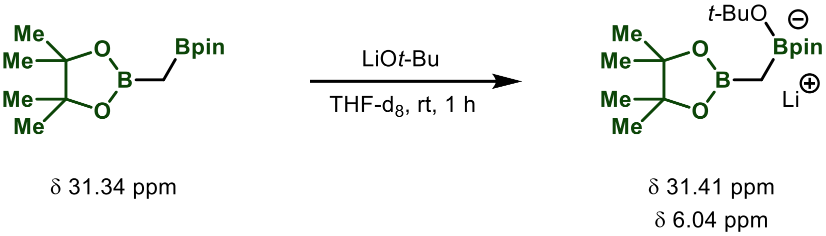

■ Possibility of Li(acac) sequestering borate by-product

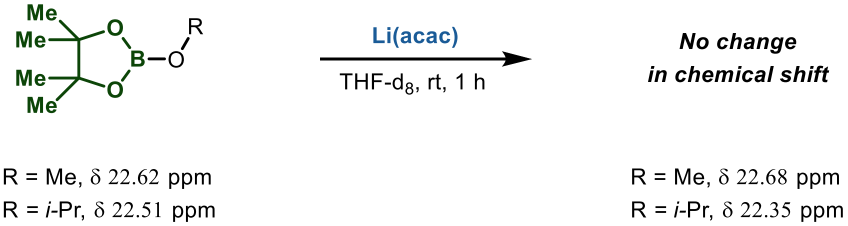

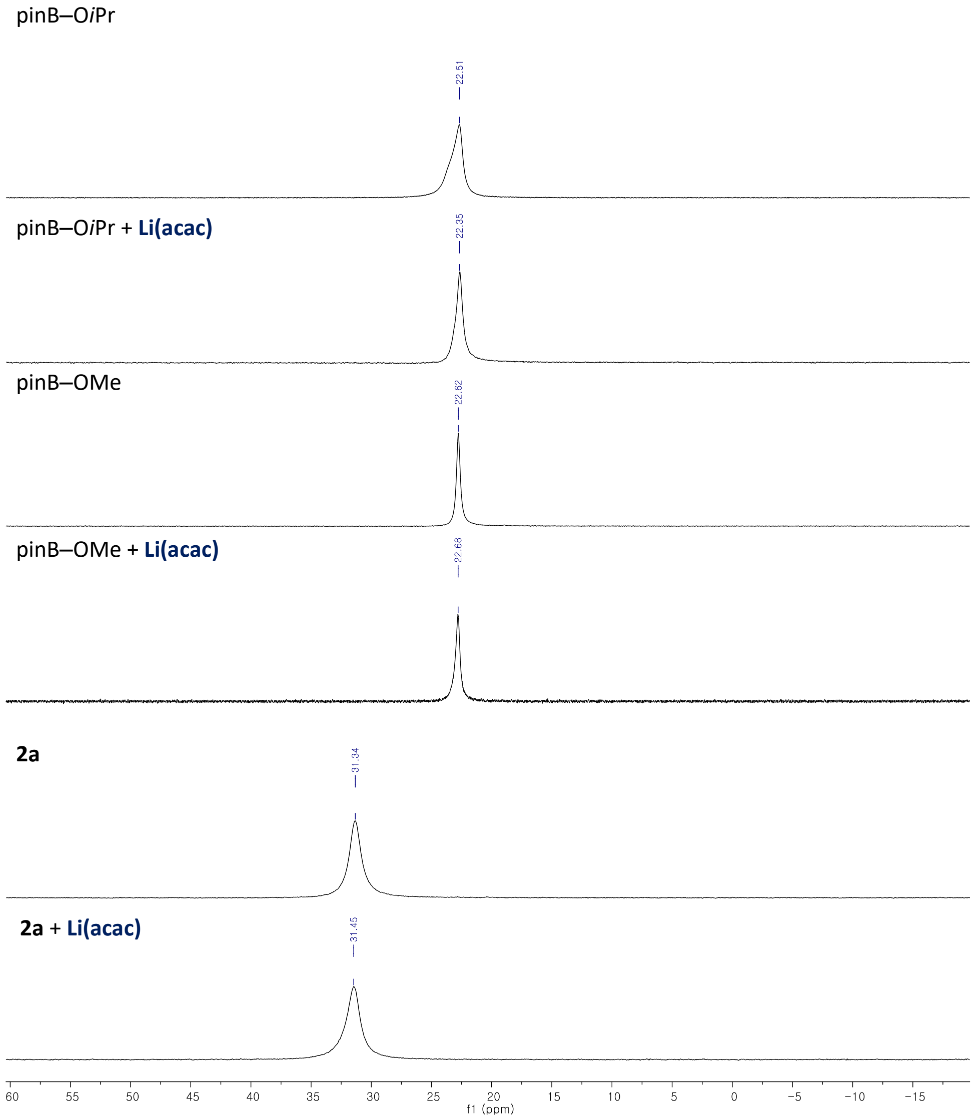

Figure S4. ^{11}B NMR spectra (128 MHz, THF-d_8, rt) of the reaction mixture

6.5. In situ monitoring of reaction system by ^{11}B NMR spectroscopies (**Fugure 1**)

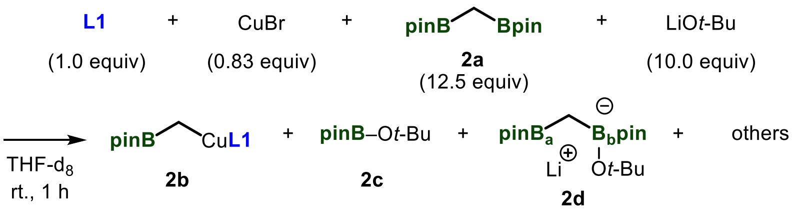

In a nitrogen-filled glove box, an oven-dried 4 mL dram-vial was charged with a magnetic stir bar, **L1** (0.012 mmol, 1.0 equiv), CuBr (0.83 equiv), 1,1-bis[(pinacolato)boryl]methane (**2a**, 12.5 equiv), LiO*t*-Bu (10 equiv) and Li(acac) (8.3 equiv). Subsequently, THF-d_8 (0.5 mL) was added to the mixture via a syringe and the system was sealed with a screw cap. After stirring for 1 hour, the reaction mixture was transferred to an NMR tube equipped with a J-Young valve. The valve was closed and then, the NMR tube was removed from the glovebox. ^{11}B NMR spectra were obtained immediately. For the control experiment, the same procedure was conducted in the absence of Li(acac). **A** = neutral Bpin species, including **2a**, **2b**, and **2d**-B_a, **B** = anionic complexes, including **2d**-B_b.

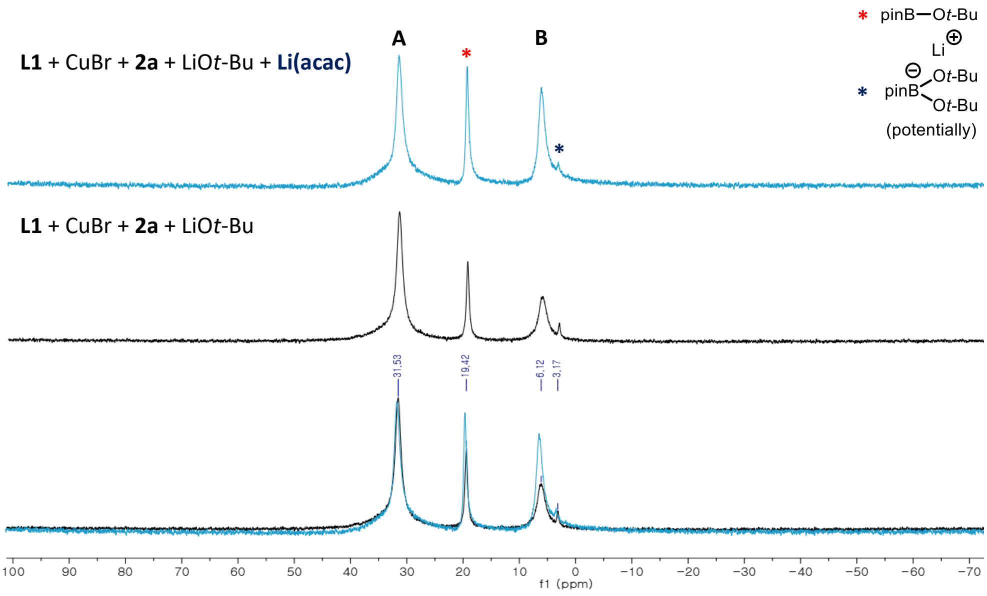

Figure S2. ^{11}B NMR spectra (128 MHz, THF-d_8, rt) of the reaction mixture

6.6. In situ monitoring of equilibrium of **2a** with LiO*t*-Bu by ^{11}B NMR spectroscopies (**Figure 2**)

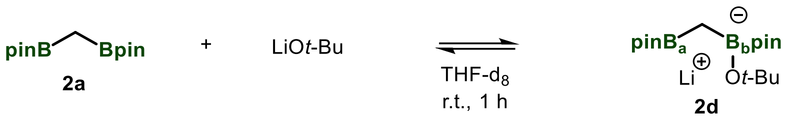

In a nitrogen-filled glove box, an oven-dried 4 mL dram-vial was charged with a magnetic stir bar, 1,1-bis[(pinacolato)boryl]methane (**2a**, 1.25 equiv, 0.15 mmol), LiO*t*-Bu (1.0 equiv), Li(acac) (0.83 equiv). Subsequently, THF-d_8 (0.5 mL) was added to the mixture via a syringe and the system was sealed with a screw cap. After stirring for 1 hour, the reaction mixture was transferred to an NMR tube equipped with a J-Young valve. The valve was closed and then, the NMR tube was removed from the glovebox. ^{11}B NMR spectra were obtained immediately. For the control experiment, the same procedure was conducted in the absence of Li(acac). **A** = neutral Bpin species, including **2a**, **2b**, and **2d**-B_a, **B** = anionic complexes, including **2d**-B_b.

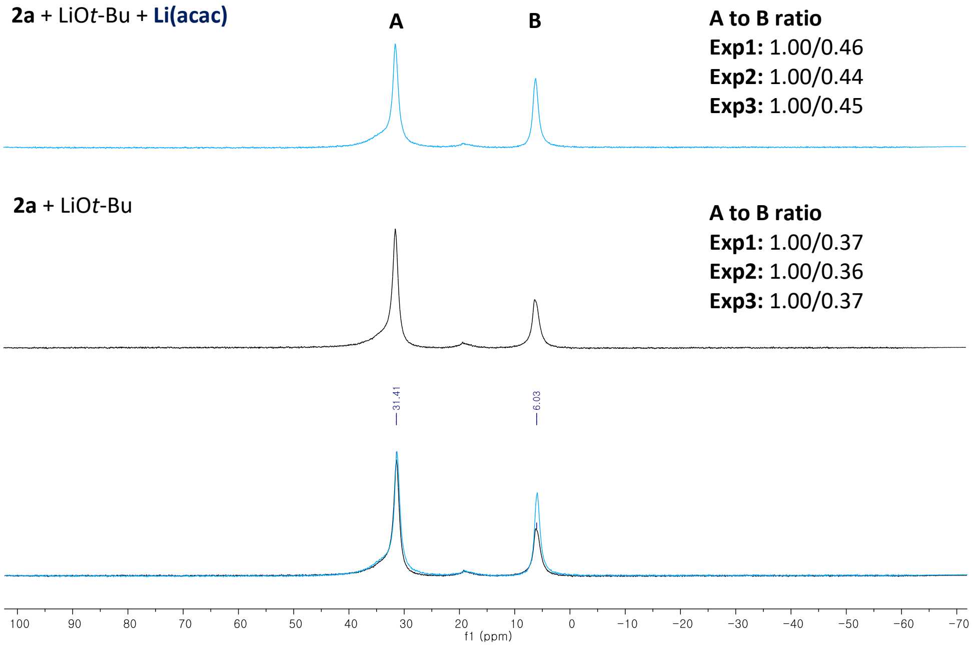

Figure S3. ^{11}B NMR spectra (128 MHz, THF-d_8, rt) of the reaction mixture

6.7. Identification of borate derivatives (**Scheme S4**)

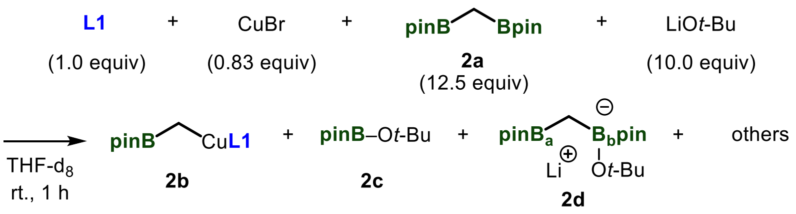

The following ^{11}B NMR data are reported (Meek 2016 and Chirik 2020).[8,9] The ^{11}B NMR spectrum of the reaction mixture consists of three major signals originating from C-ligated neutral Bpin species (δ 31.5 ppm), including **2a**, **2b**, and **2d**-B_a, heteroatom-bound neutral Bpin species (**2c**, δ 19.4 ppm), and anionic complexes, such as **2d**-B_b (δ 6.12 ppm) or Li[pinB(O*t*-Bu)$_2$] (δ 3.17 ppm).

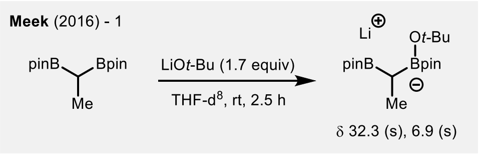

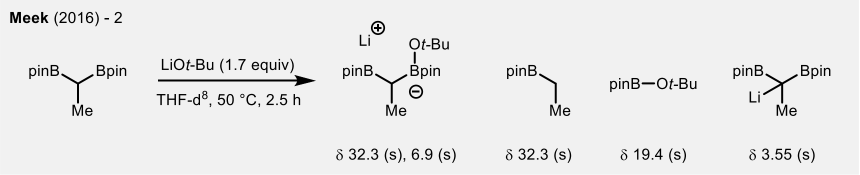

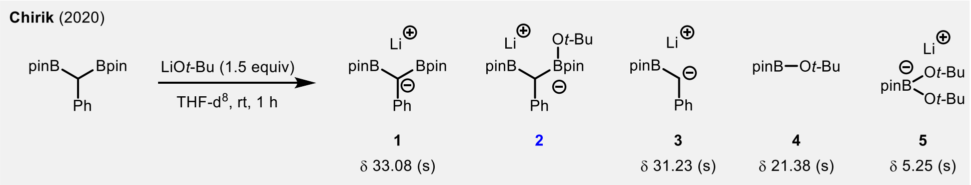

Meek (2016) - 1

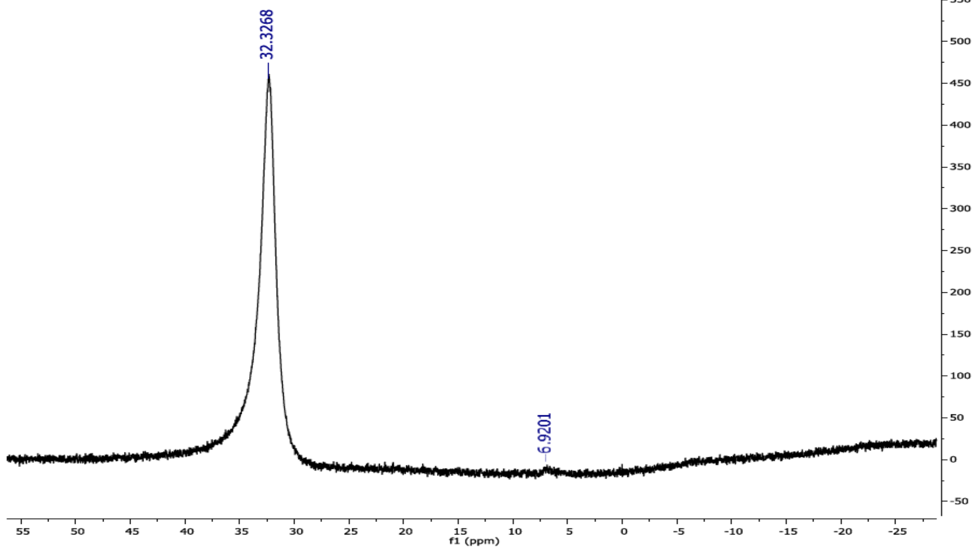

Meek (2016) - 2

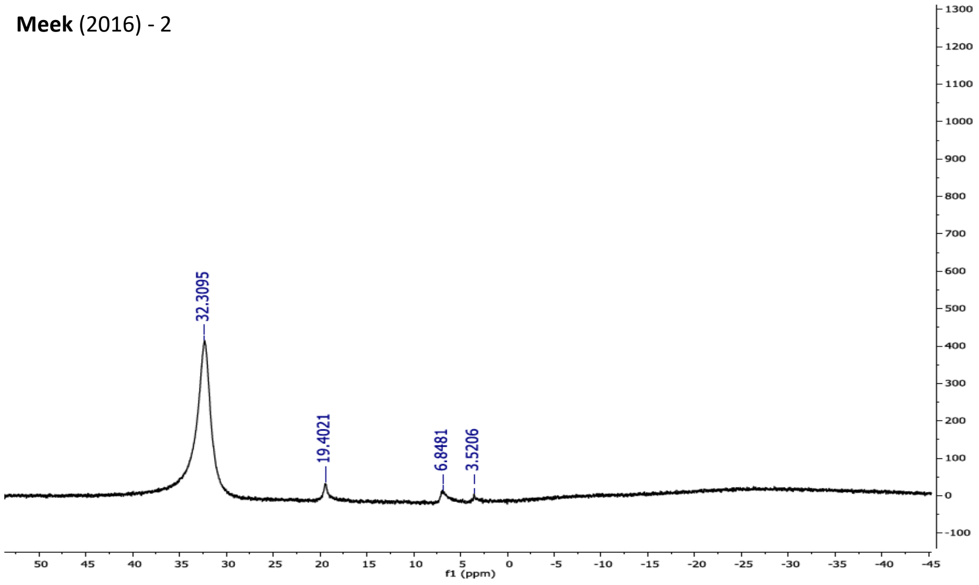

Chirik (2020)

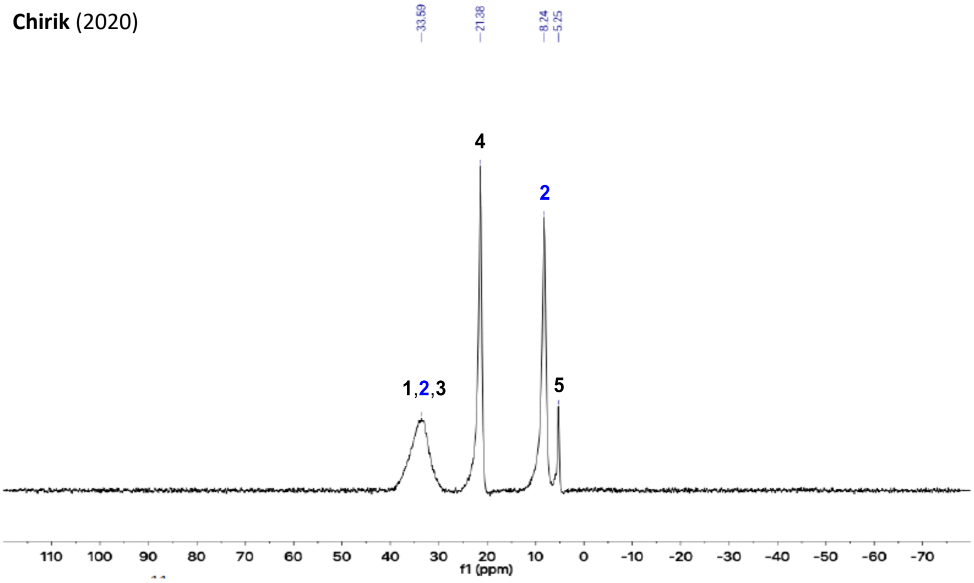
33.59
21.38
8.24
5.25
4
2
1,2,3
5
110 100 90 80 70 60 50 40 30 20 10 0 -10 -20 -30 -40 -50 -60 -70
f1 (ppm)

7. HRMS Data

7.1. General procedure for monitoring of decomposition pathway of (*S*)-MonoPhos

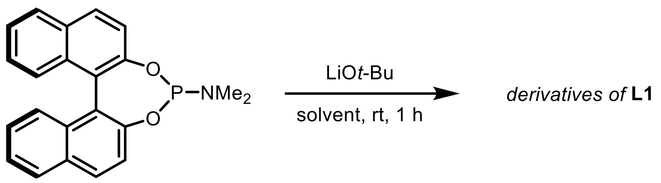

(*S*)-MonoPhos (**L1**)

Condition A. In a nitrogen-filled glove box, an oven-dried 4 mL dram-vial was charged with a magnetic stir bar, (*S*)-MonoPhos (0.012 mmol, 1.0 equiv). Subsequently, acetonitrile (0.5 mL) was added to the mixture via a syringe and the system was sealed with a screw cap. After stirring for 1 hour, the mixture was diluted in acetonitrile for HRMS analysis. HRMS were recorded within 1 hour.

Condition B. In a nitrogen-filled glove box, an oven-dried 4 mL dram-vial was charged with a magnetic stir bar, (*S*)-MonoPhos (0.012 mmol, 1.0 equiv), and LiO*t*-Bu (10 equiv). Subsequently, anhydrous THF (0.5 mL) was added to the mixture via a syringe and the system was sealed with a screw cap. After stirring for 1 hour, the reaction mixture was filtered and concentrated under reduced pressure. The filtrate was diluted in acetonitrile for HRMS analysis. HRMS were recorded within 1 hour.

Condition C. In a nitrogen-filled glove box, an oven-dried 4 mL dram-vial was charged with a magnetic stir bar, (*S*)-MonoPhos (0.012 mmol, 1.0 equiv), and LiO*t*-Bu (10 equiv). Subsequently, acetonitrile (0.5 mL) was added to the mixture via a syringe and the system was sealed with a screw cap. After stirring for 1 hour, the vial was removed from the glovebox. The reaction mixture was filtered quickly and HRMS were recorded within 1 hour.

Condition D. A 4 mL dram-vial was charged with a magnetic stir bar, (*S*)-MonoPhos (0.012 mmol, 1.0 equiv), and LiO*t*-Bu (10 equiv). Subsequently, acetonitrile (0.5 mL) was added to the mixture via a syringe. After stirring 1 hour under air, brine (5 mL) was added in one portion to quench the reaction. The aqueous phase was separated and extracted with ethyl acetate (3 × 10 mL). The combined organic phase was dried over anhydrous sodium sulfate (Na_2SO_4), filtered, and concentrated under reduced pressure. Subsequently, the crude product was diluted with acetonitrile for HMRS analysis. HRMS were recorded within 1 hour.

7.2. The observation of the related phosphoramidite-based species

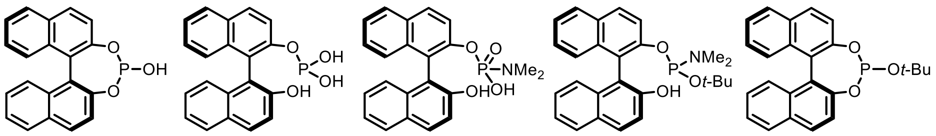

**L11**	**L12**	**L13**	**L14**	**L15**
calcd: 333.0675	calcd: 351.0781	calcd: 394.1203	calcd: 434.1879	calcd: 389.1301
found: 333.0677	found: 351.0784	found: 394.1205	found: 434.1881	found: 389.1298

7.3. Plausible mechanistic scenario

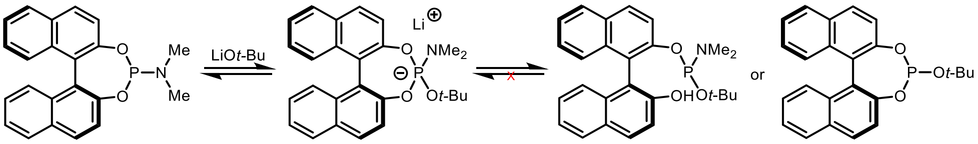

7.4. HRMS analysis (ESI-MS, $[M+H]^+$)

(a) Under condition A (**L1** in MeCN)

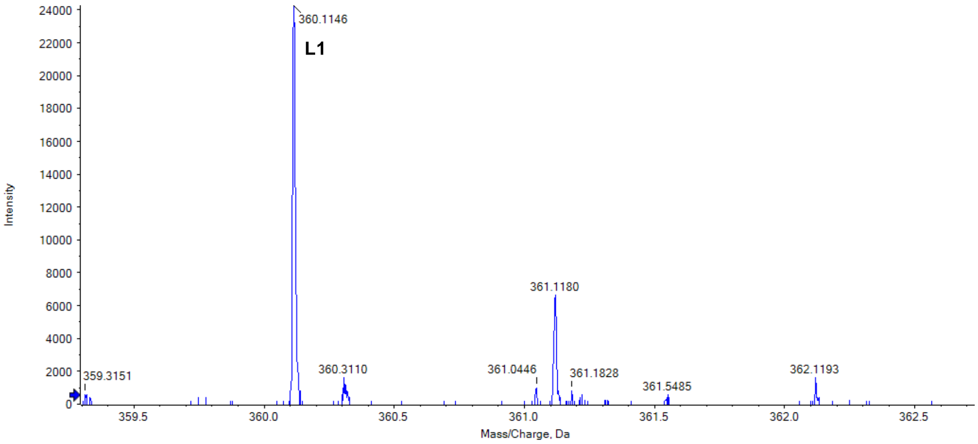

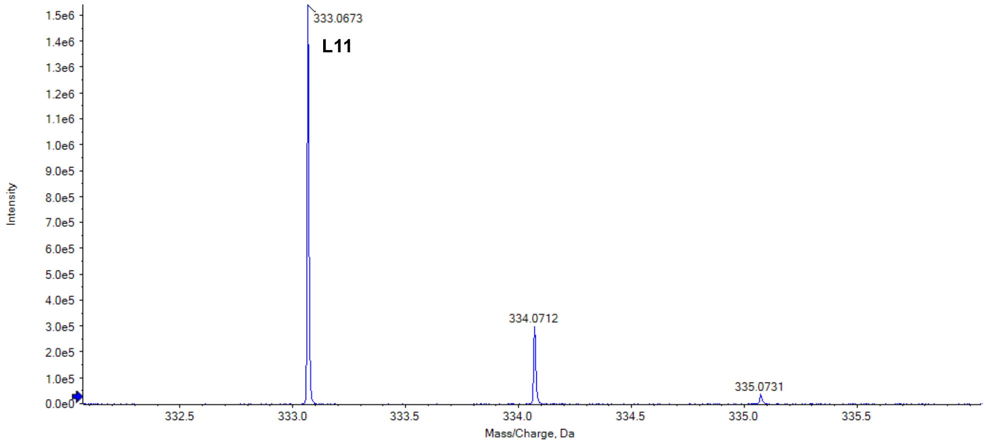
333.0673
L11
334.0712
335.0731
Intensity
Mass/Charge, Da

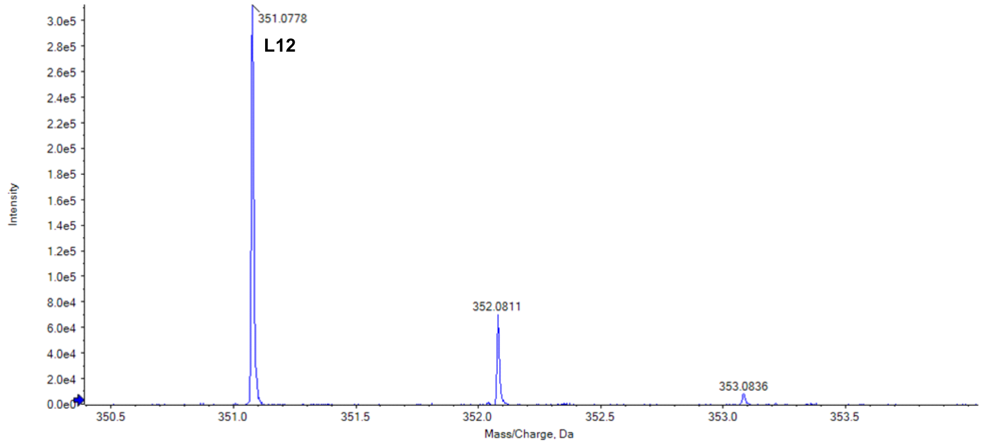
351.0778
L12
352.0811
353.0836
Intensity
Mass/Charge, Da

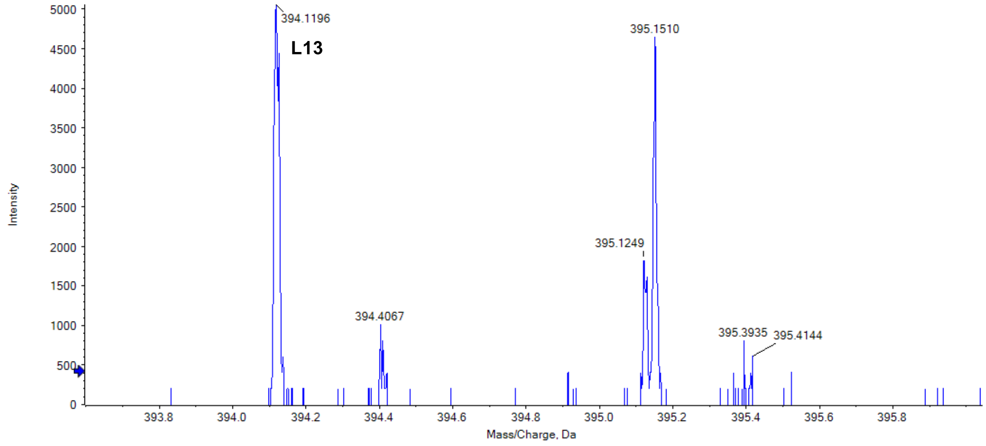
394.1196
L13
395.1510
395.1249
394.4067
395.3935
395.4144
Intensity
Mass/Charge, Da

(b) Under condition B (**L1** + LiO*t*-Bu in THF)

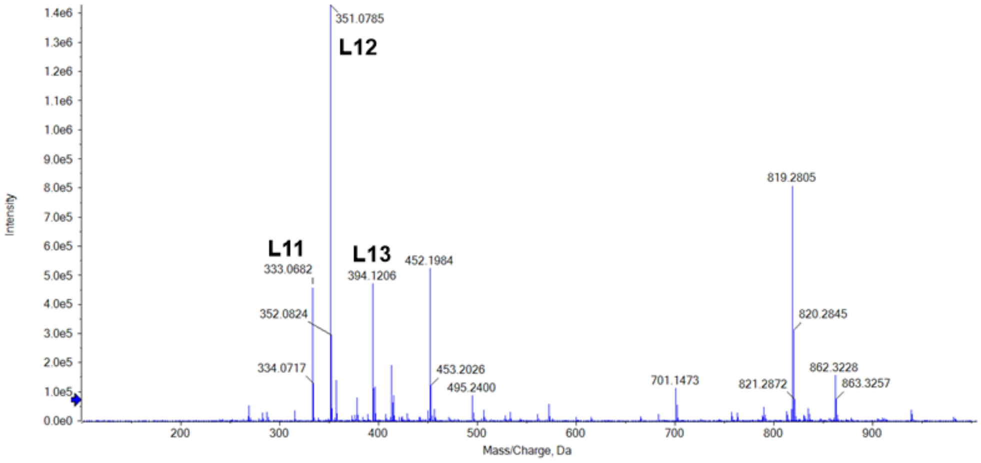

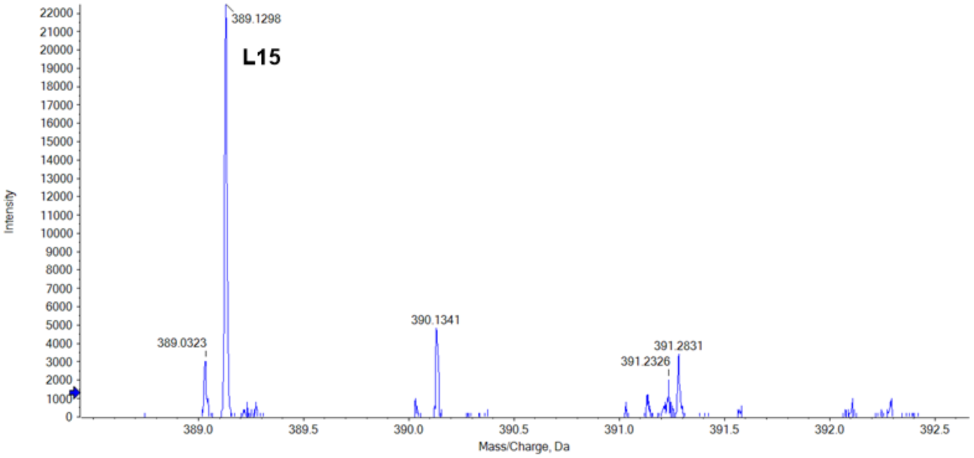

(c) Under condition C (**L1** + LiO*t*-Bu in MeCN)

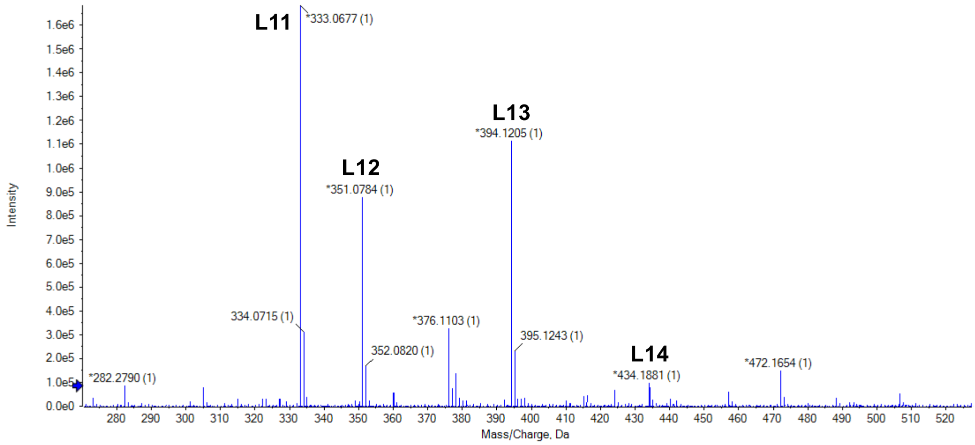

(d) Under condition D (**L1** + LiO*t*-Bu in MeCN and work-up)

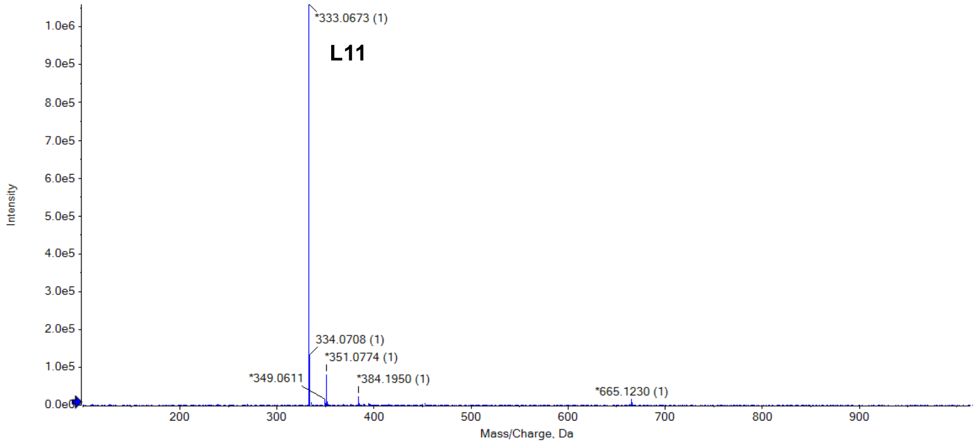

8. References.

1. For preparation of enone **2e**, see: I. Kazi, S. Guha, G. Sekar, *Org. Lett.*, 2017, **19**, 5, 1244.
2. For preparation of enone **3p**, see: J. Kan, M. Zhang, X. Zhang, X. Lou, Y. Shang, B. Xu, F. Yang, W. Su, *Chem. Eur. J*, 2019, **25**, 15233.
3. For preparation of enone **3r**, see: J.-J. Cao, F. Zhou, J. Zhou, *Angew. Chem.*, *Int. Ed.*, 2010, **49**, 4976.
4. S. Matsumura, Y. Maeda, T. Nishimura, S. Umeura, *J. Am. Chem. Soc.*, 2003, **125**, 8862.
5. S. Kin, A. E. Nibbs, Y. E. Türkmen, V. H. Rawal, *J. Am. Chem. Soc.*, 2013, **135**, 16050.
6. (a) X. Liang, K. Wei, Y.-R. Yang, *Chem. Commun*. 2015, **51**, 17471; (b) S. J. Gharpure, D. S. Vishwakarma, S. K. Nanda, *Org. Lett.*, 2017, **19**, 6534; (c) F. Zhao, N. Li, T. Zhang, Z.-Y. Han, S.-W. Luo, L.-Z. Gong, *Angew. Chem.*, *Int. Ed.*, 2017, **56**, 3247.
7. F. von. Rekowski, C. Koch, R. M. Gschwind, *J. Am. Chem. Soc*. 2014, **136**, 11389.
8. M. V. Joannou, B. S. Moyer, S. J. Meek, *J. Am. Chem. Soc.*, 2015, **137**, 6176.
9. B. Lee, P. J. Chirik, *J. Am. Chem. Soc.* 2020, **142**, 2429

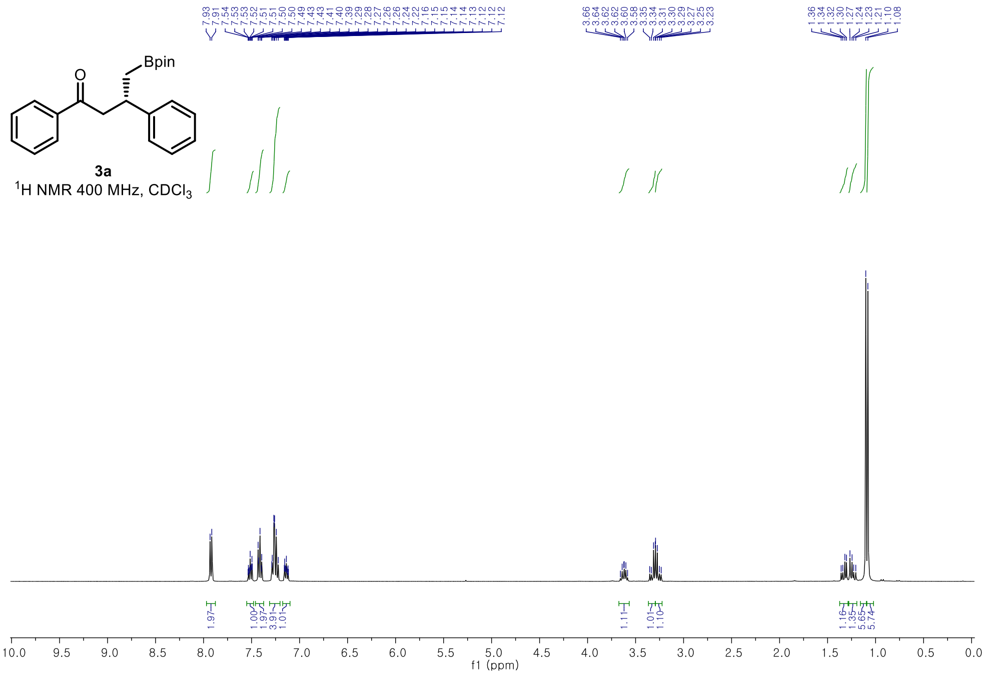

3a

^{1}H NMR 400 MHz, $CDCl_3$

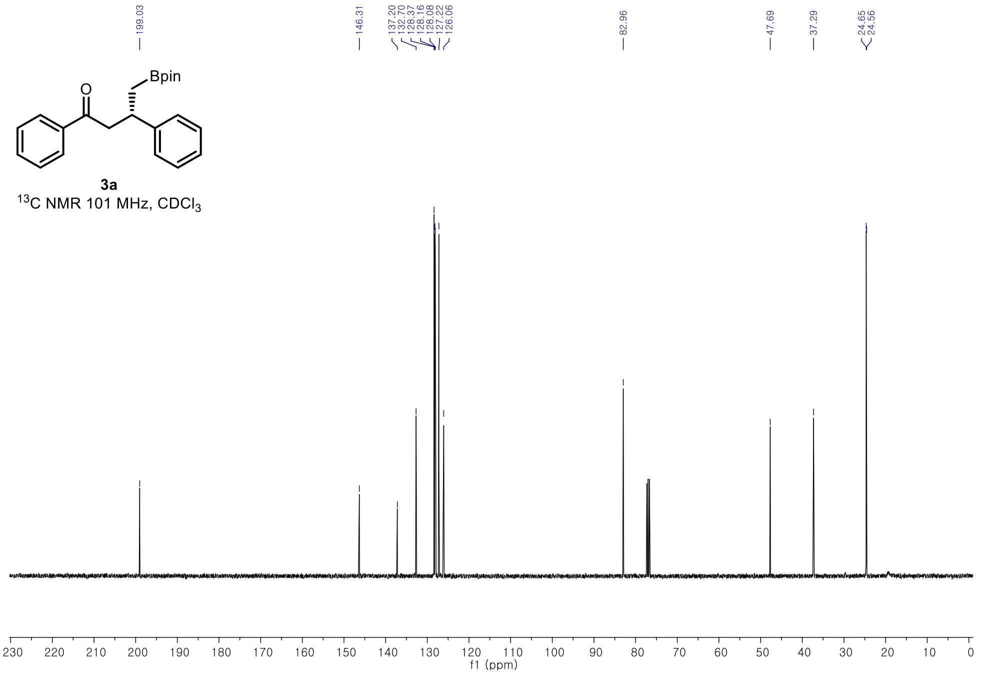

3a

^{13}C NMR 101 MHz, $CDCl_3$

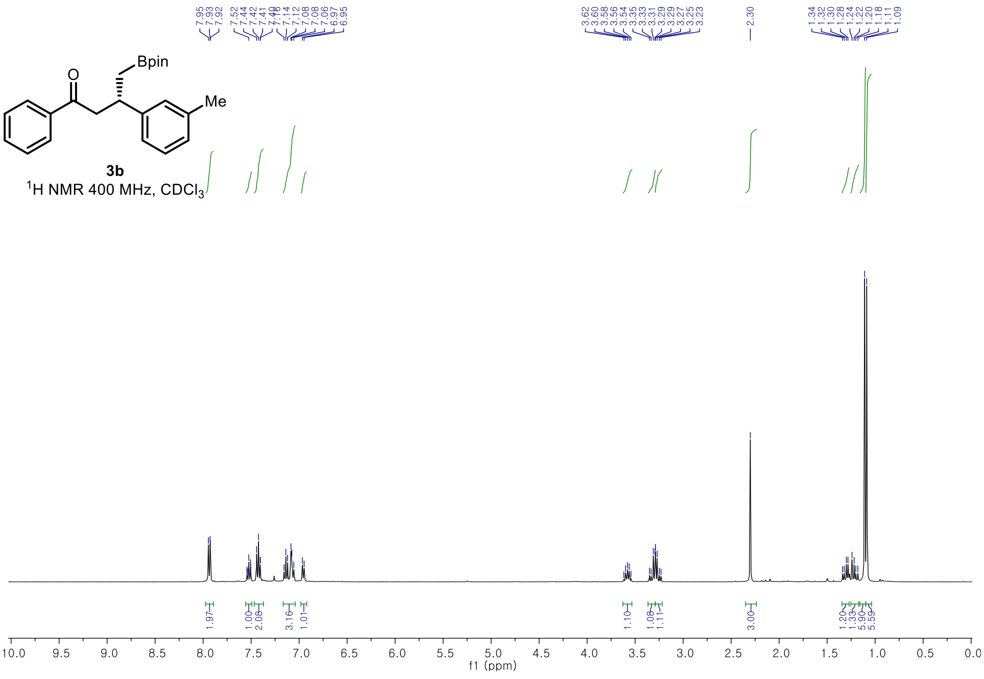
Bpin
O
Me
3b
^{1}H NMR 400 MHz, CDCl$_3$
f1 (ppm)

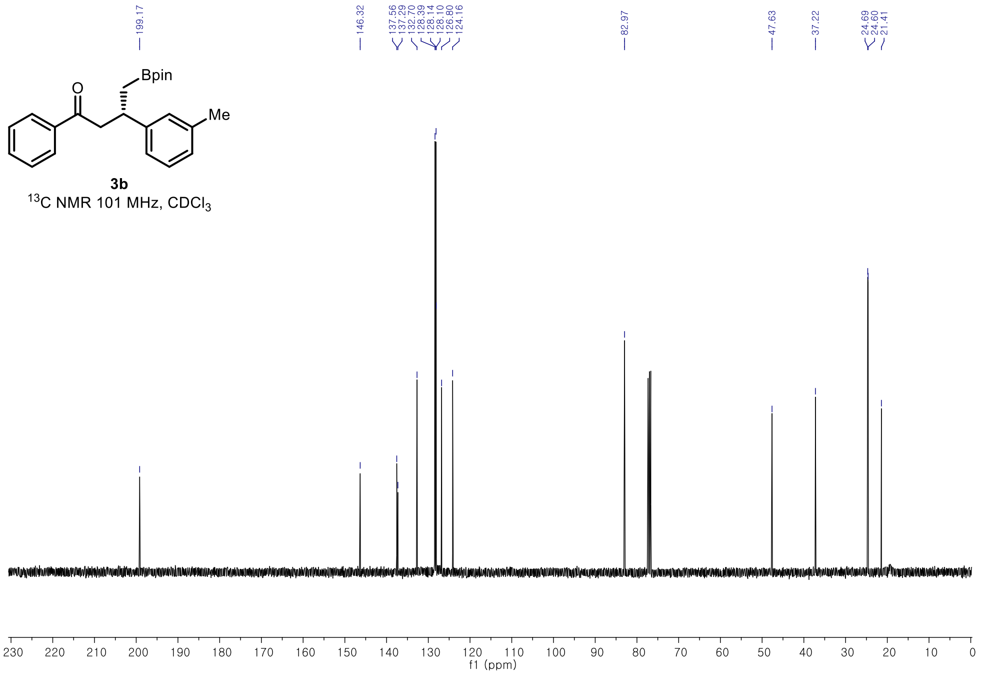
Bpin
O
Me
3b
^{13}C NMR 101 MHz, CDCl$_3$
f1 (ppm)

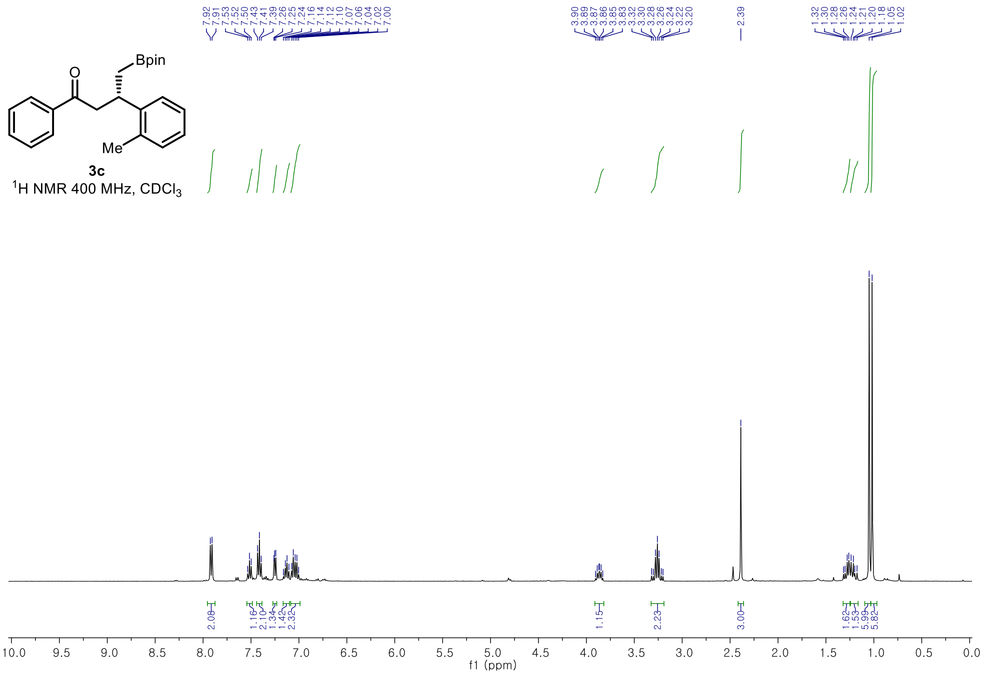
Bpin
O
Me
3c
^{1}H NMR 400 MHz, CDCl$_3$
f1 (ppm)

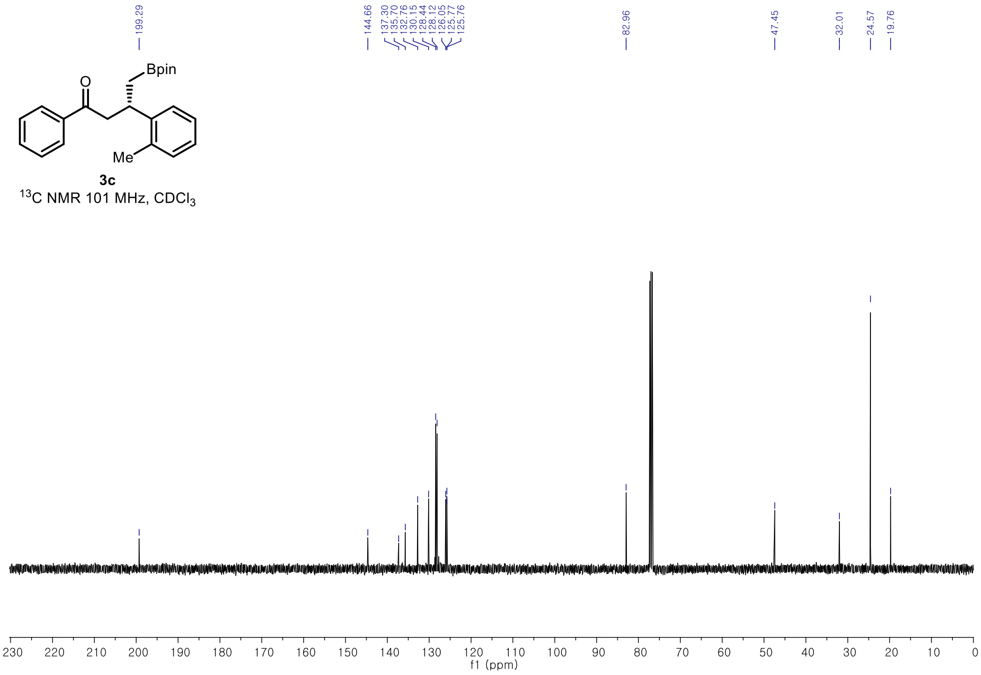
Bpin
O
Me
3c
^{13}C NMR 101 MHz, CDCl$_3$
199.29
144.66
82.96
47.45
32.01
24.57
19.76
f1 (ppm)

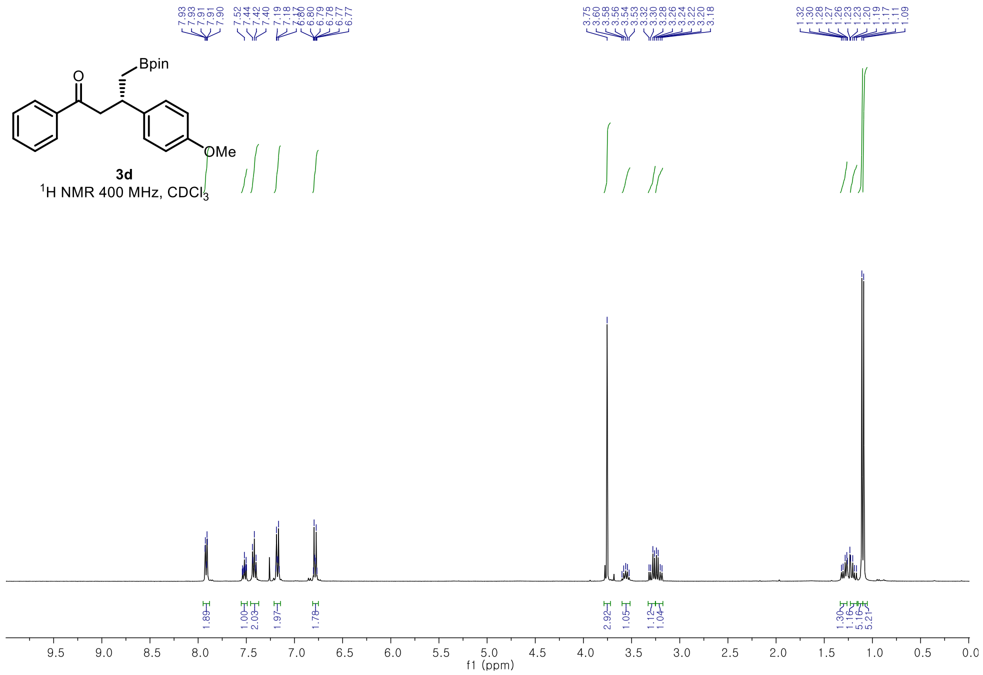
Bpin
O
OMe
3d
^{1}H NMR 400 MHz, $CDCl_3$
7.93 7.93 7.91 7.91 7.90 7.52 7.44 7.42 7.40 7.19 7.18 7.17 6.80 6.79 6.78 6.77 6.77
3.75 3.60 3.58 3.56 3.54 3.53 3.32 3.30 3.28 3.26 3.24 3.22 3.20 3.18
1.32 1.30 1.28 1.27 1.26 1.23 1.23 1.20 1.19 1.17 1.11 1.09
1.89 1.00 2.03 1.97 1.78 2.92 1.05 1.12 1.04 1.30 1.16 5.16 5.21
9.5 9.0 8.5 8.0 7.5 7.0 6.5 6.0 5.5 5.0 4.5 4.0 3.5 3.0 2.5 2.0 1.5 1.0 0.5 0.0
f1 (ppm)

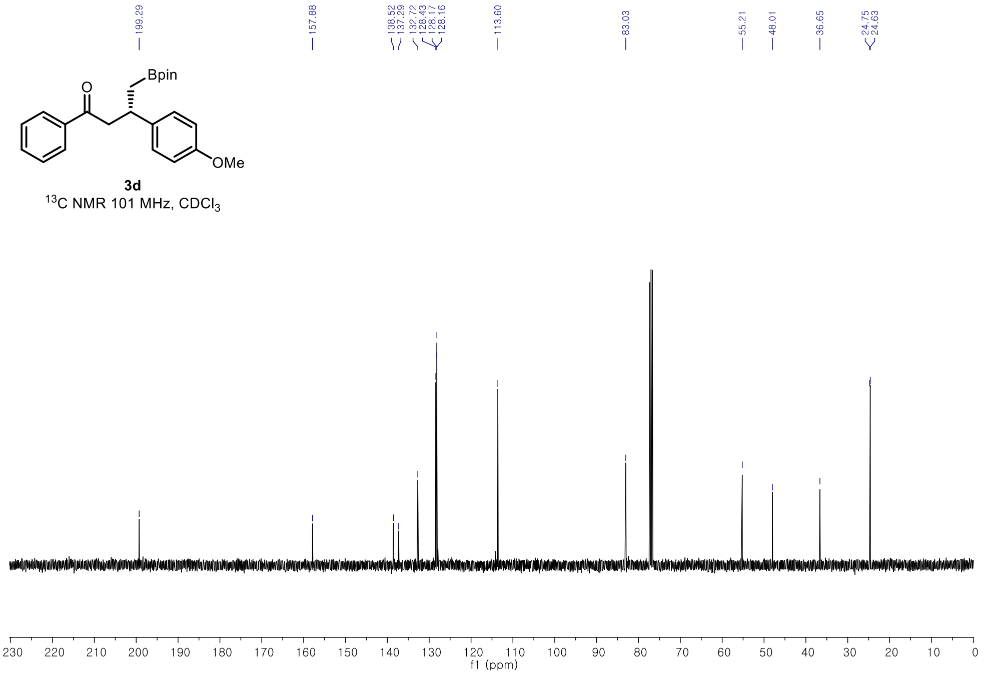
199.29 157.88 138.52 137.29 132.72 128.43 128.17 128.16 113.60 83.03 55.21 48.01 36.65 24.75 24.63
Bpin
O
OMe
3d
^{13}C NMR 101 MHz, $CDCl_3$
230 220 210 200 190 180 170 160 150 140 130 120 110 100 90 80 70 60 50 40 30 20 10 0
f1 (ppm)

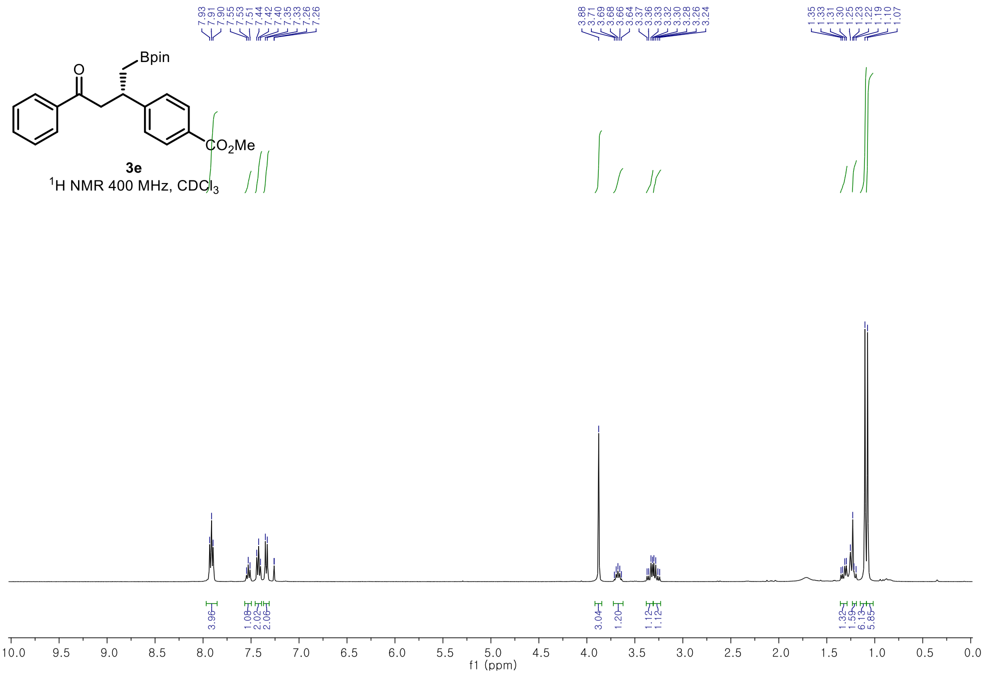
7.93 7.91 7.90 7.55 7.53 7.51 7.44 7.42 7.40 7.35 7.33 7.26 7.26
3.88 3.71 3.69 3.68 3.66 3.64 3.37 3.36 3.33 3.32 3.30 3.28 3.26 3.24
1.35 1.33 1.31 1.30 1.25 1.23 1.22 1.19 1.10 1.07
Bpin
O
CO_2Me
3e
^1H NMR 400 MHz, $CDCl_3$
3.96 1.08 2.02 2.06 3.04 1.20 1.12 1.12 1.32 1.59 6.13 5.85
f1 (ppm)

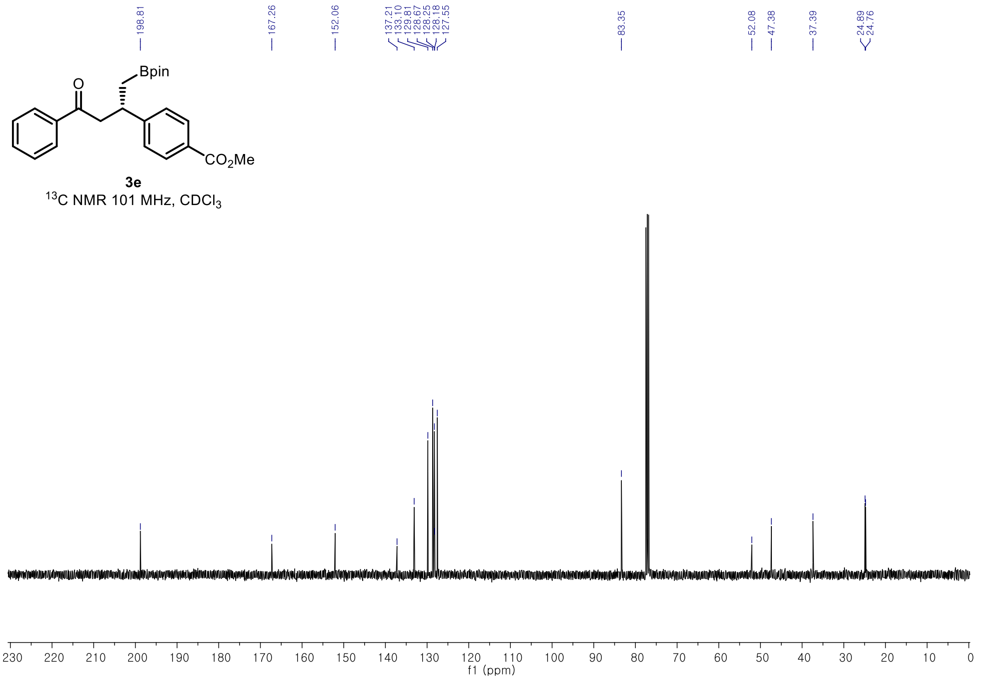
198.81
167.26
152.06
137.21 133.10 129.81 128.67 128.25 128.18 127.55
83.35
52.08
47.38
37.39
24.89 24.76
Bpin
O
CO_2Me
3e
^{13}C NMR 101 MHz, $CDCl_3$
f1 (ppm)

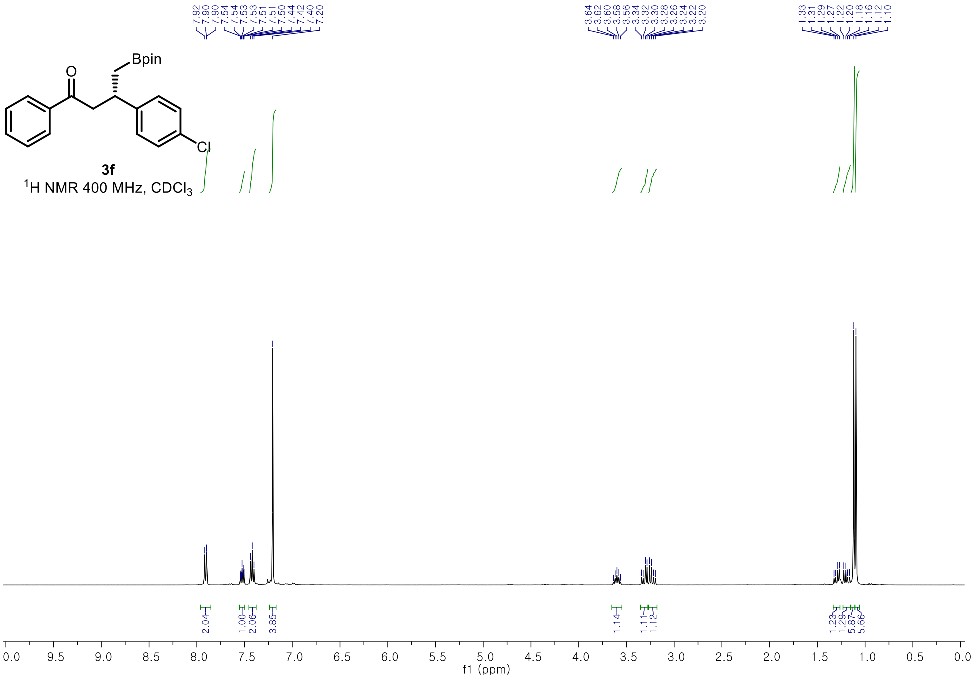

3f
^{1}H NMR 400 MHz, $CDCl_3$

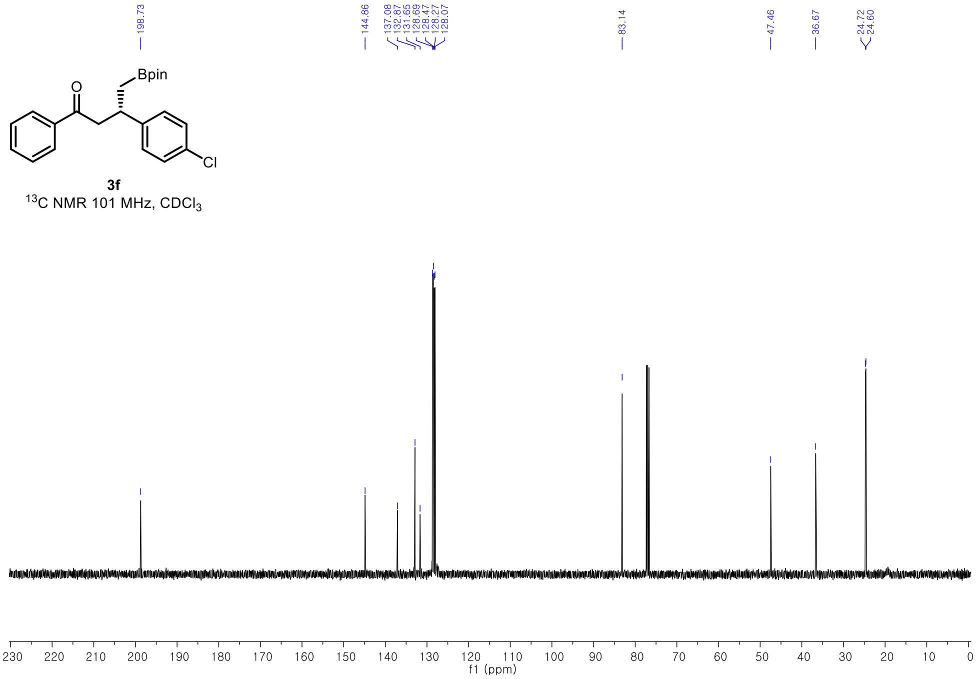

3f
^{13}C NMR 101 MHz, $CDCl_3$

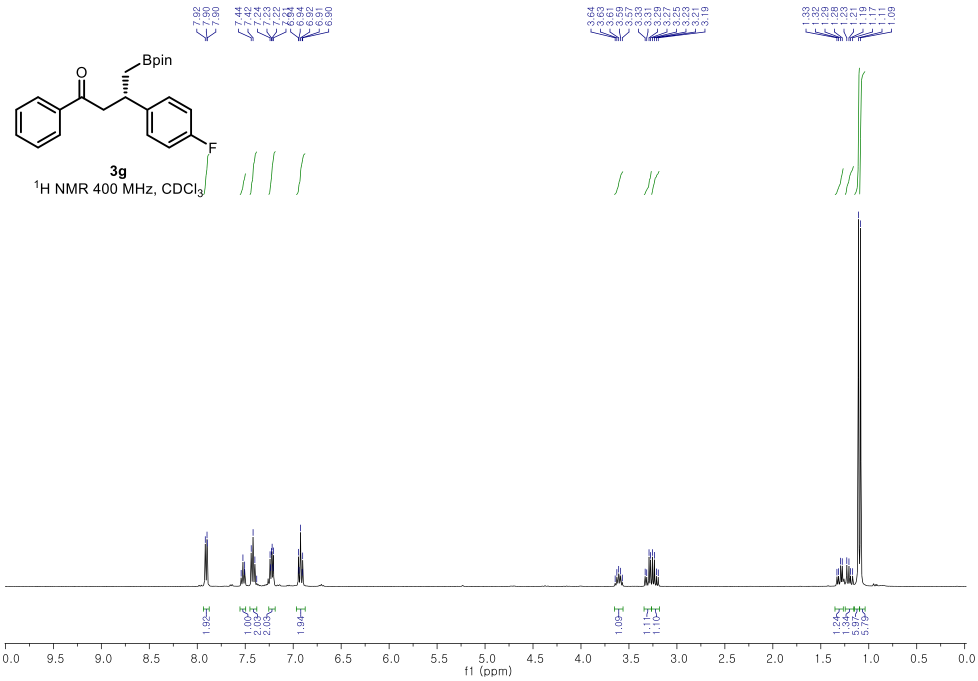

Bpin
O
F
3g
1H NMR 400 MHz, CDCl3
f1 (ppm)

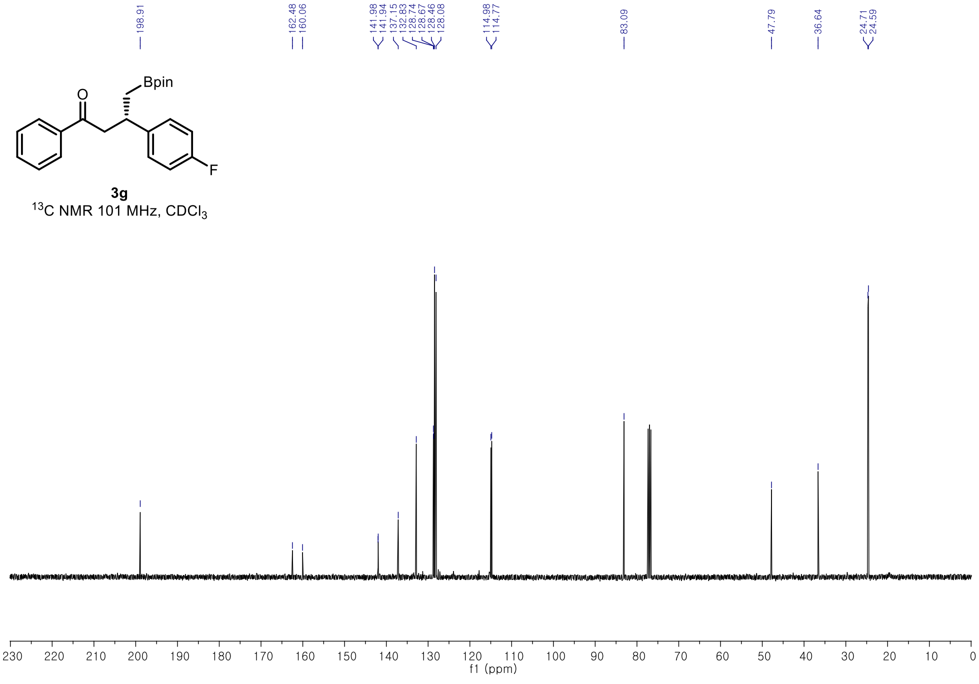

Bpin
O
F
3g
13C NMR 101 MHz, CDCl3
f1 (ppm)

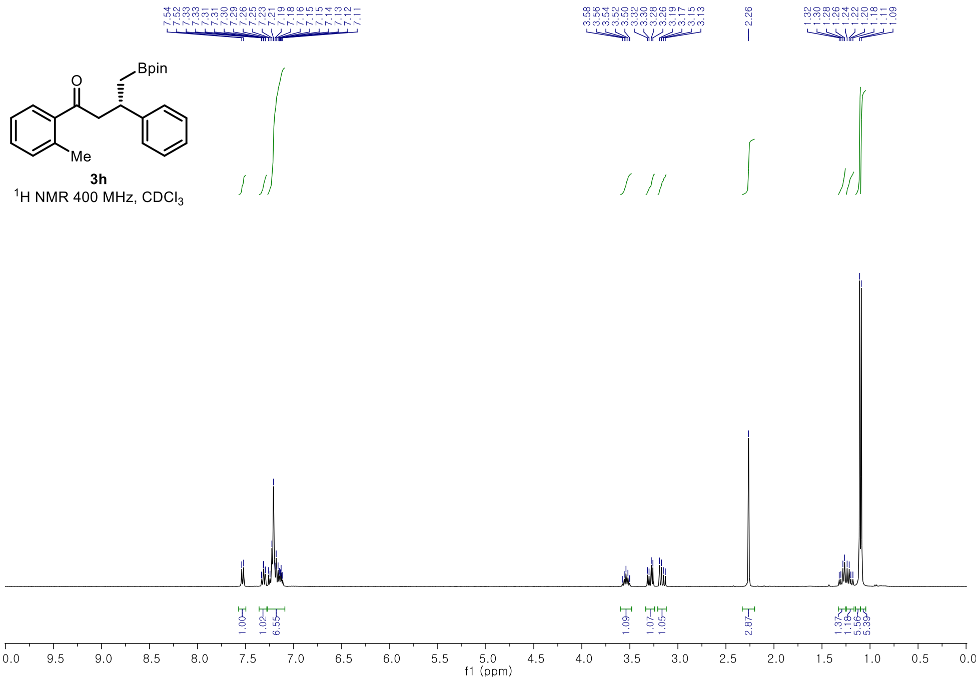

3h
^{1}H NMR 400 MHz, $CDCl_3$

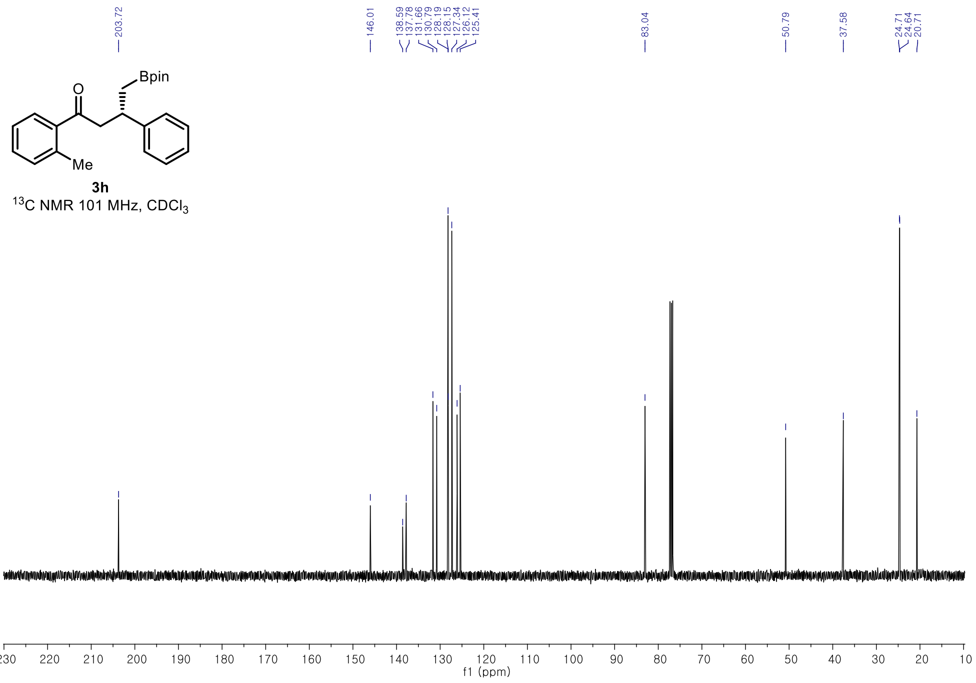

3h
^{13}C NMR 101 MHz, $CDCl_3$

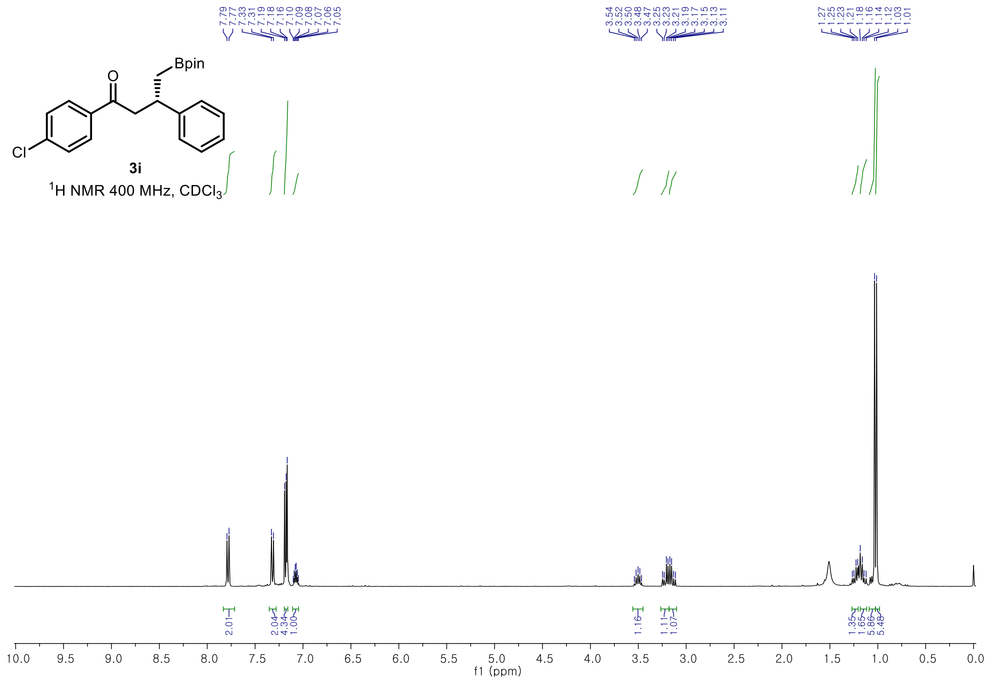

3i

^{1}H NMR 400 MHz, $CDCl_3$

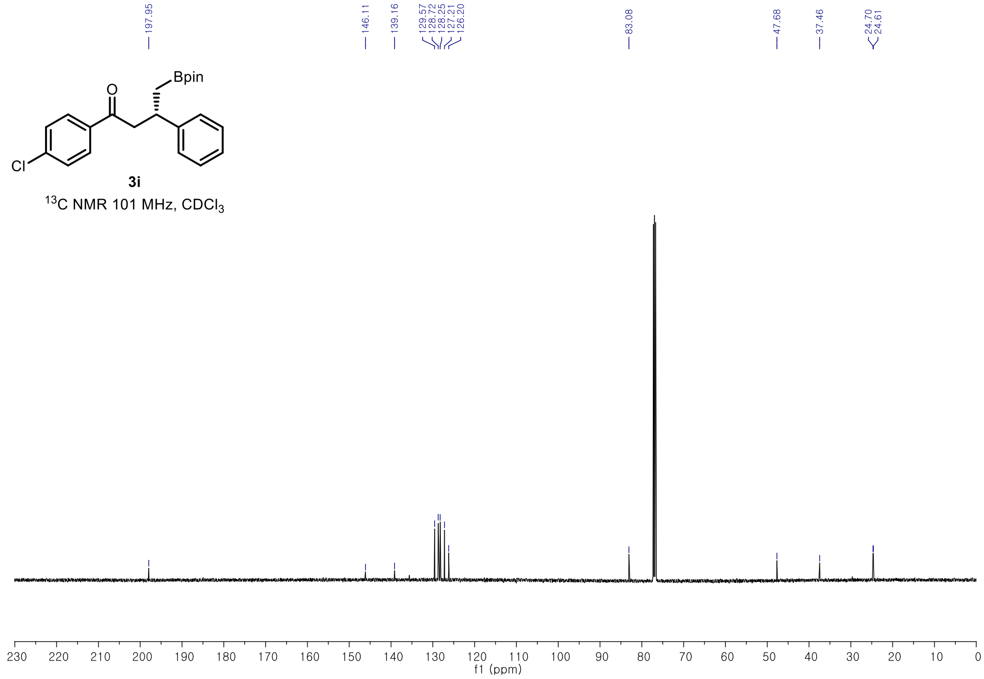

3i

^{13}C NMR 101 MHz, $CDCl_3$

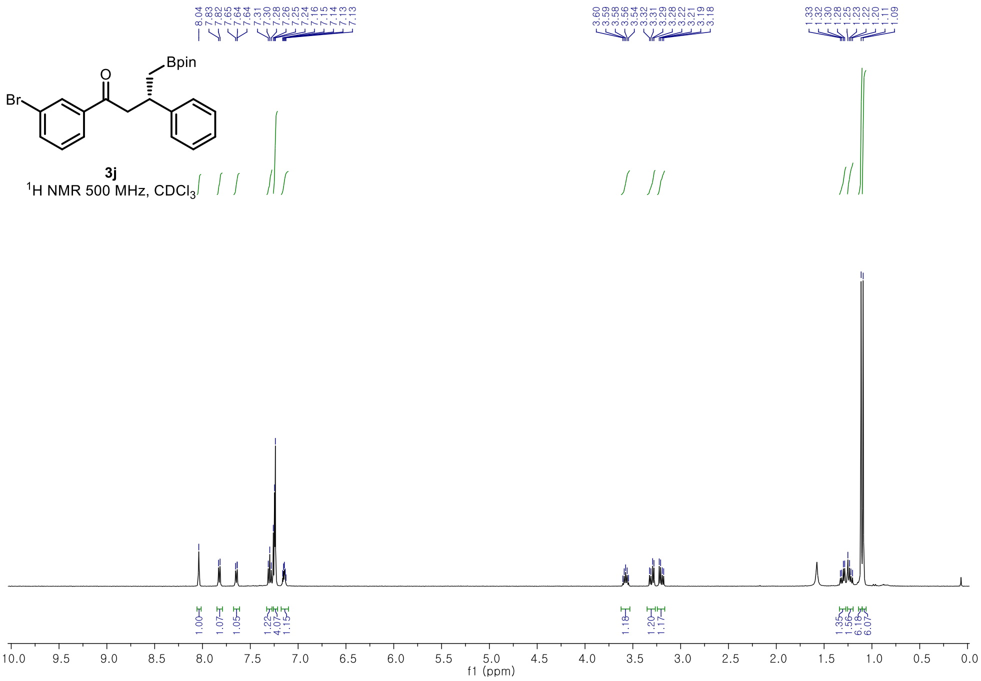
Bpin
O
Br
3j
^{1}H NMR 500 MHz, $CDCl_3$
8.04 7.83 7.82 7.65 7.64 7.31 7.30 7.28 7.26 7.25 7.24 7.16 7.15 7.14 7.13 7.13
3.60 3.59 3.58 3.56 3.54 3.32 3.31 3.29 3.28 3.22 3.21 3.19 3.18
1.33 1.32 1.30 1.28 1.25 1.23 1.22 1.20 1.11 1.09
1.00 1.07 1.05 1.22 4.07 1.15 1.18 1.20 1.17 1.35 1.56 6.18 6.07
f1 (ppm)

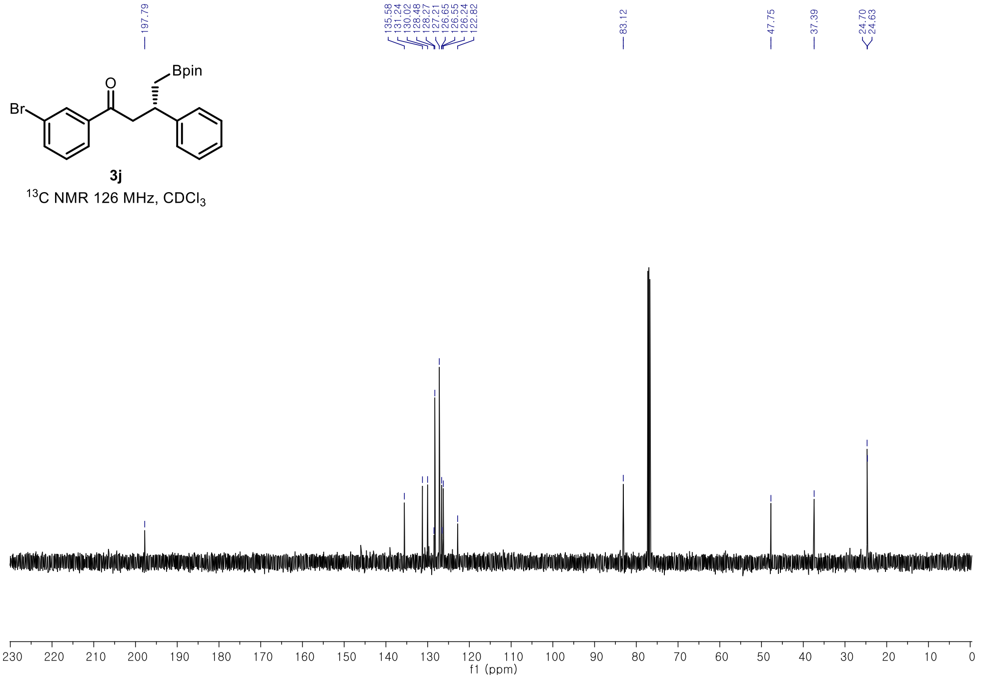
Bpin
O
Br
3j
^{13}C NMR 126 MHz, $CDCl_3$
197.79
135.58 131.24 130.02 128.48 128.27 127.21 126.65 126.55 126.24 122.82
83.12
47.75
37.39
24.70 24.63
f1 (ppm)

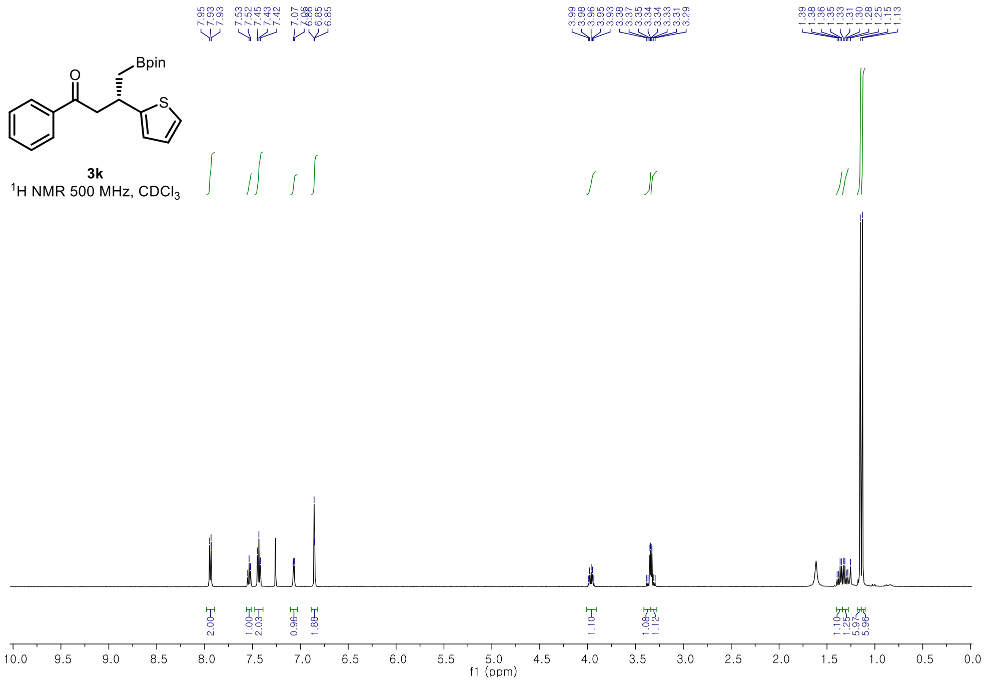
7.95
7.93
7.93
7.53
7.52
7.45
7.43
7.42
7.07
7.06
6.86
6.85
6.85
3.99
3.98
3.96
3.95
3.93
3.38
3.37
3.35
3.34
3.34
3.33
3.31
3.29
1.39
1.38
1.36
1.35
1.33
1.31
1.30
1.28
1.25
1.15
1.13
Bpin
O
S
3k
^{1}H NMR 500 MHz, $CDCl_3$
2.00
1.00
2.03
0.96
1.88
1.10
1.06
1.12
1.10
1.25
5.97
5.96
f1 (ppm)

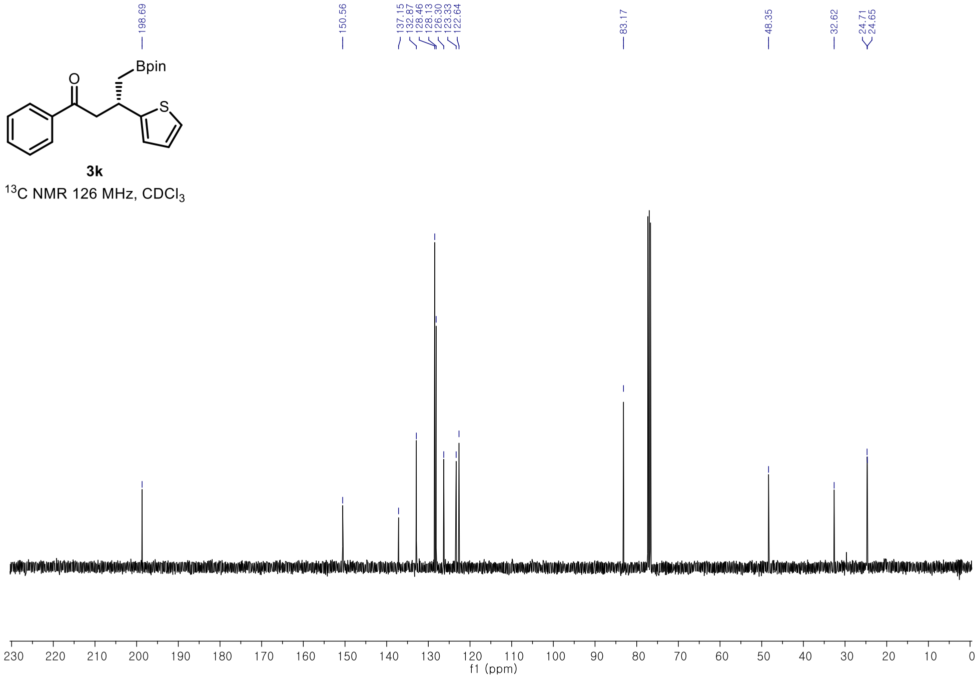
198.69
150.56
137.15
132.87
128.46
128.13
126.30
123.33
122.64
83.17
48.35
32.62
24.71
24.65
Bpin
O
S
3k
^{13}C NMR 126 MHz, $CDCl_3$
f1 (ppm)

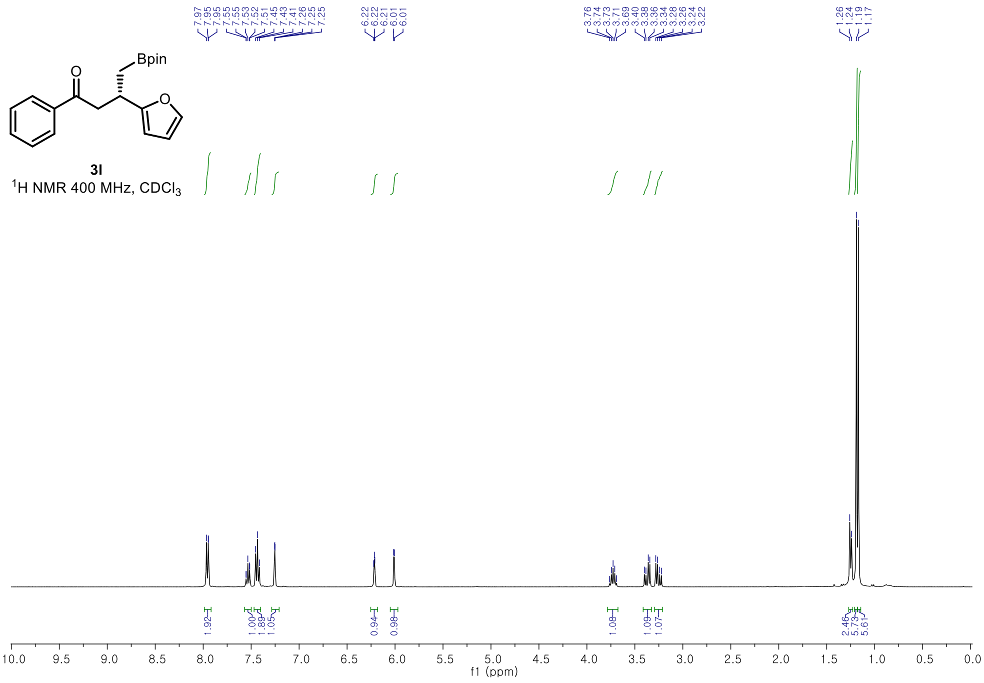
Bpin
O
3l
1H NMR 400 MHz, CDCl3
7.97
7.95
7.95
7.55
7.53
7.52
7.51
7.45
7.43
7.41
7.26
7.25
7.25
6.22
6.22
6.21
6.01
6.01
3.76
3.74
3.73
3.71
3.69
3.40
3.38
3.36
3.34
3.28
3.26
3.24
3.22
1.26
1.24
1.19
1.17
1.92
1.00
1.89
1.05
0.94
0.98
1.08
1.09
1.07
2.46
5.73
5.61
10.0
9.5
9.0
8.5
8.0
7.5
7.0
6.5
6.0
5.5
5.0
4.5
4.0
3.5
3.0
2.5
2.0
1.5
1.0
0.5
0.0
f1 (ppm)

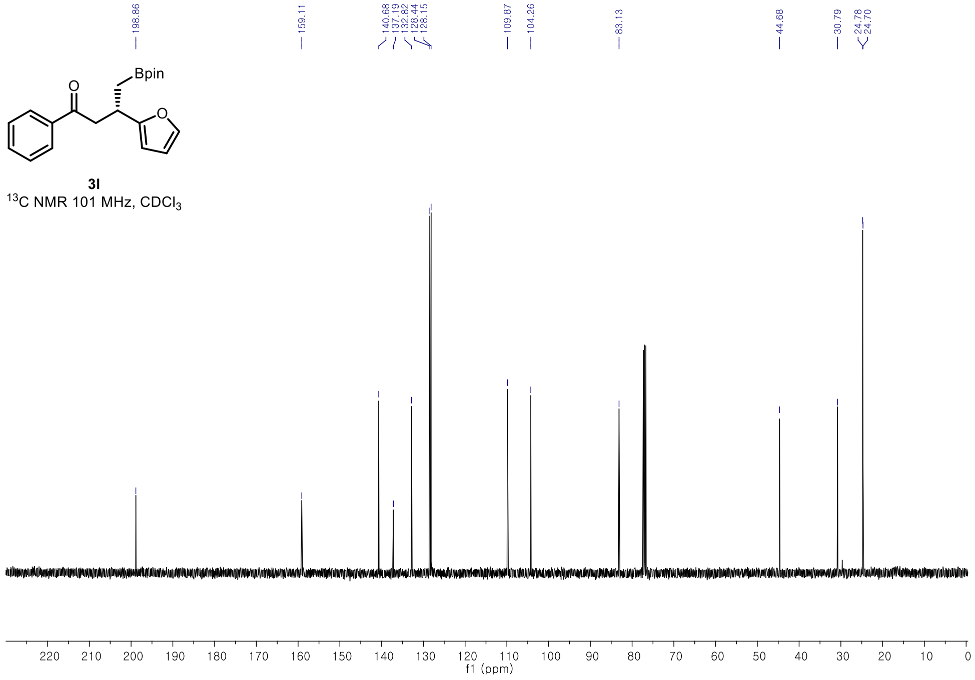
Bpin
O
3l
13C NMR 101 MHz, CDCl3
198.86
159.11
140.68
137.19
132.82
128.44
128.15
109.87
104.26
83.13
44.68
30.79
24.78
24.70
220
210
200
190
180
170
160
150
140
130
120
110
100
90
80
70
60
50
40
30
20
10
0
f1 (ppm)

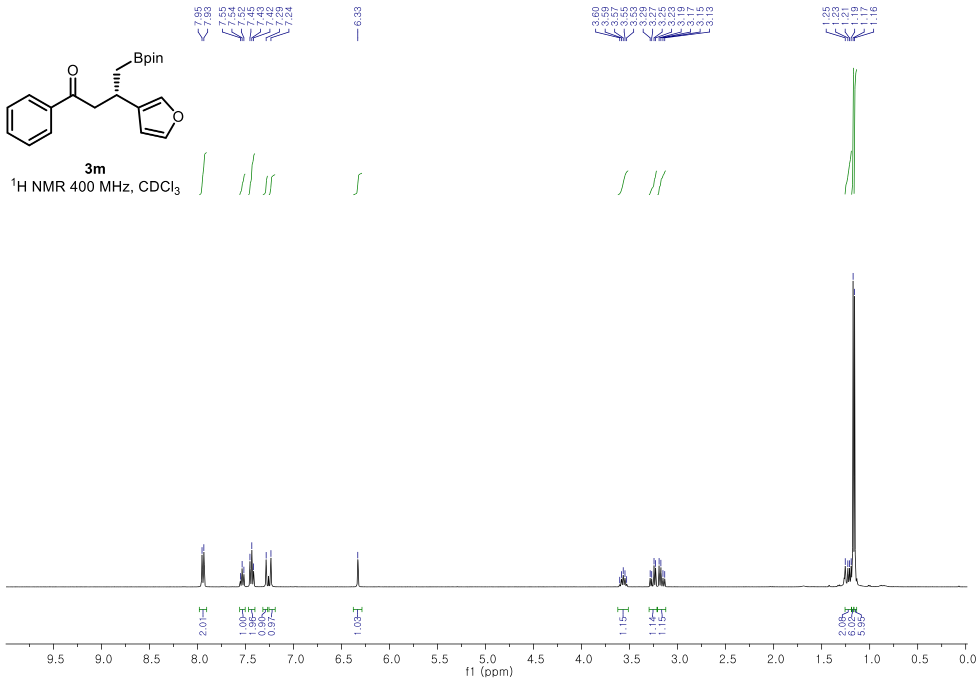

3m
^{1}H NMR 400 MHz, $CDCl_3$

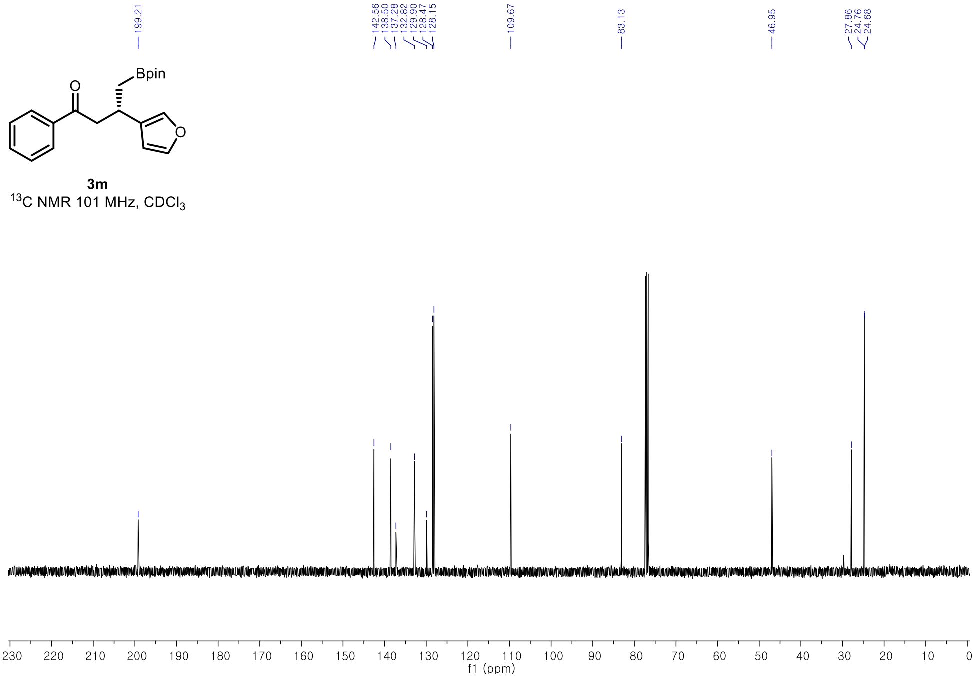

3m
^{13}C NMR 101 MHz, $CDCl_3$

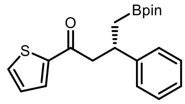

3n

^{1}H NMR 500 MHz, $CDCl_3$

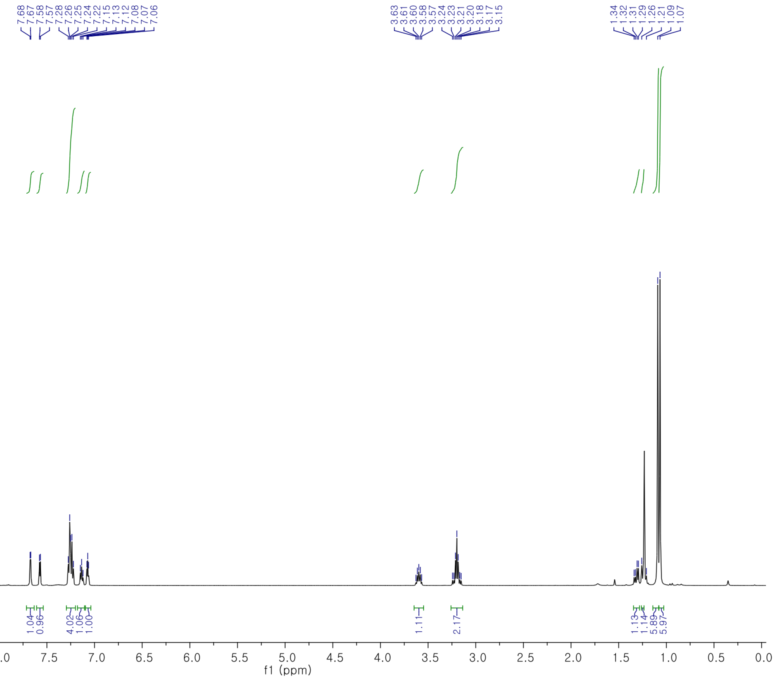

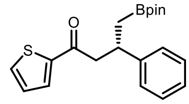

3n

^{13}C NMR 126 MHz, $CDCl_3$

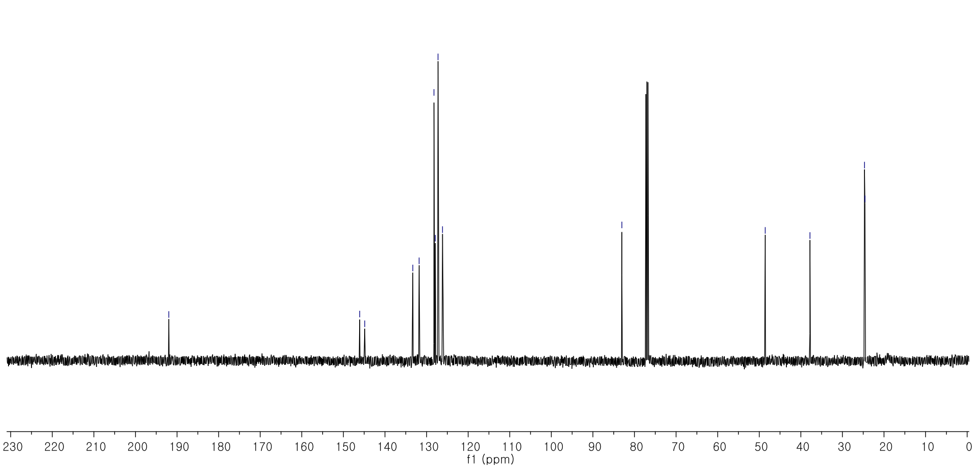

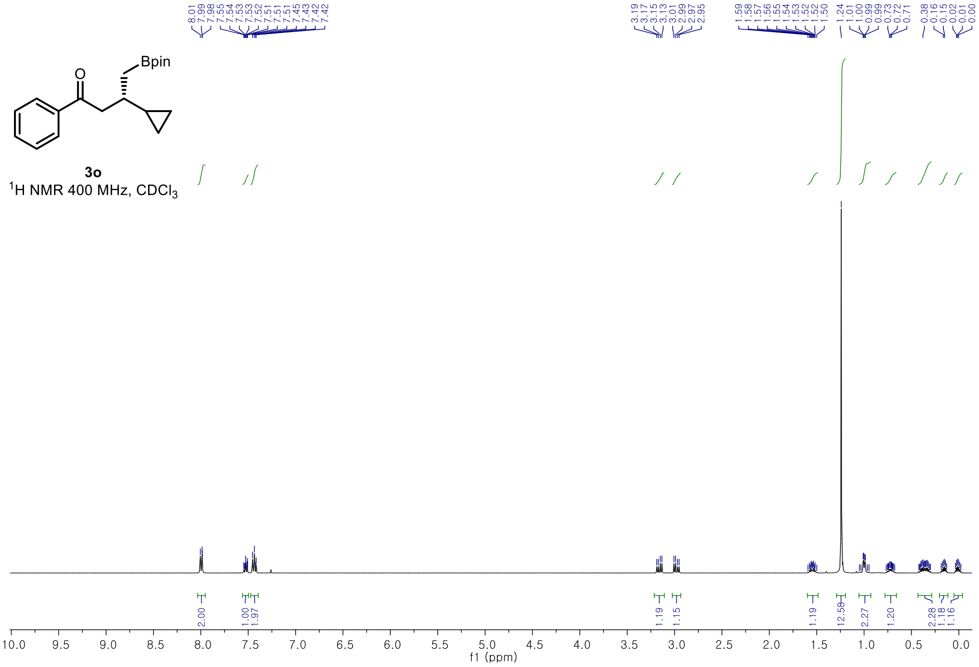
Bpin
O
3o
^{1}H NMR 400 MHz, $CDCl_3$
f1 (ppm)

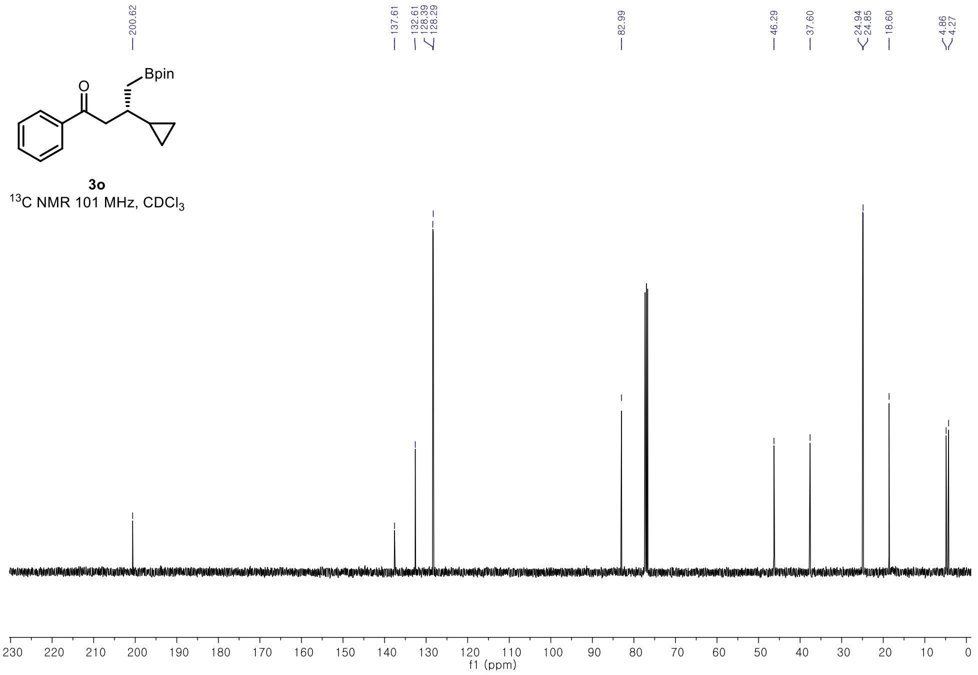
Bpin
O
3o
^{13}C NMR 101 MHz, $CDCl_3$
f1 (ppm)

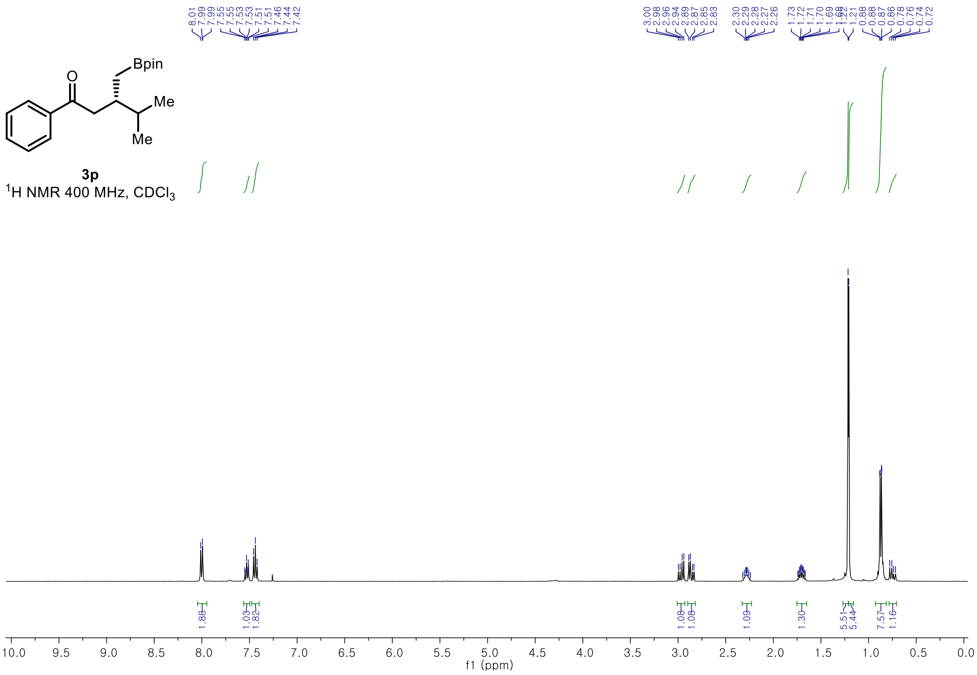

3p
^{1}H NMR 400 MHz, $CDCl_3$

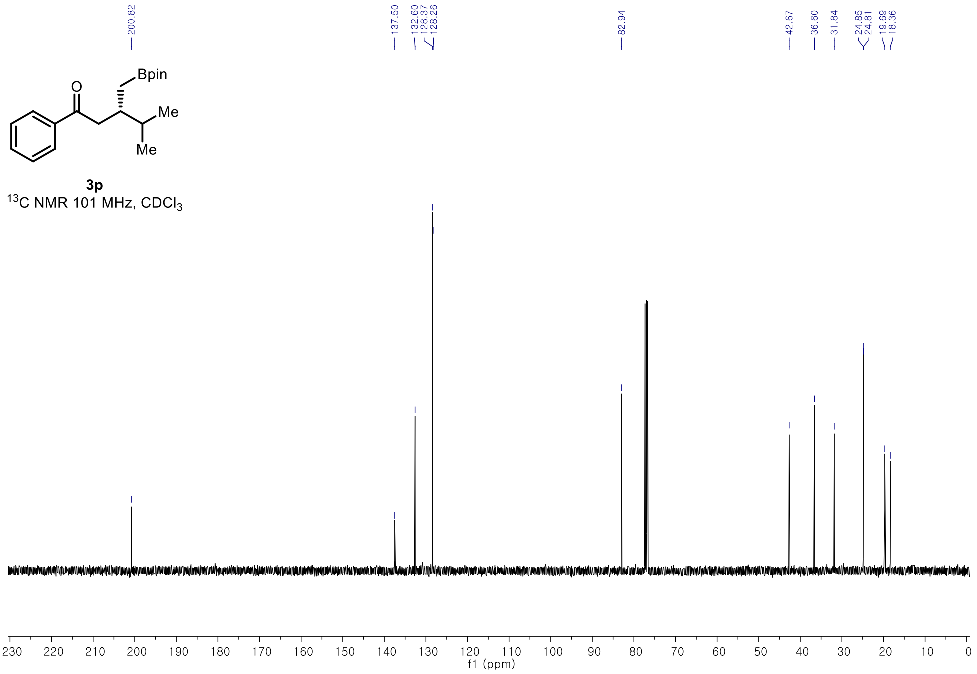

3p
^{13}C NMR 101 MHz, $CDCl_3$

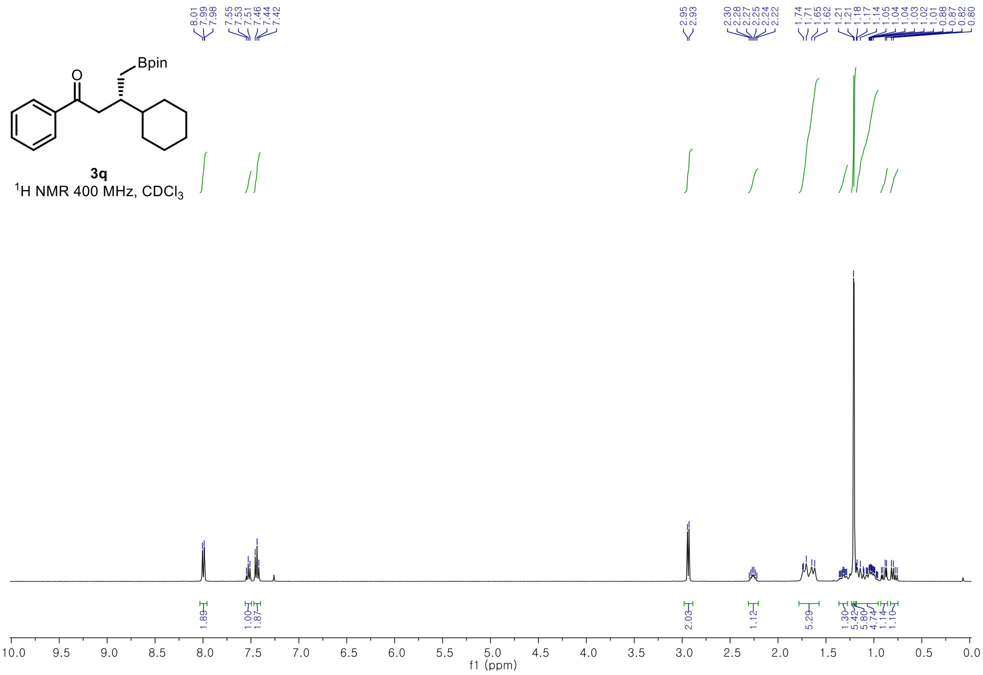

3q
^{1}H NMR 400 MHz, $CDCl_3$

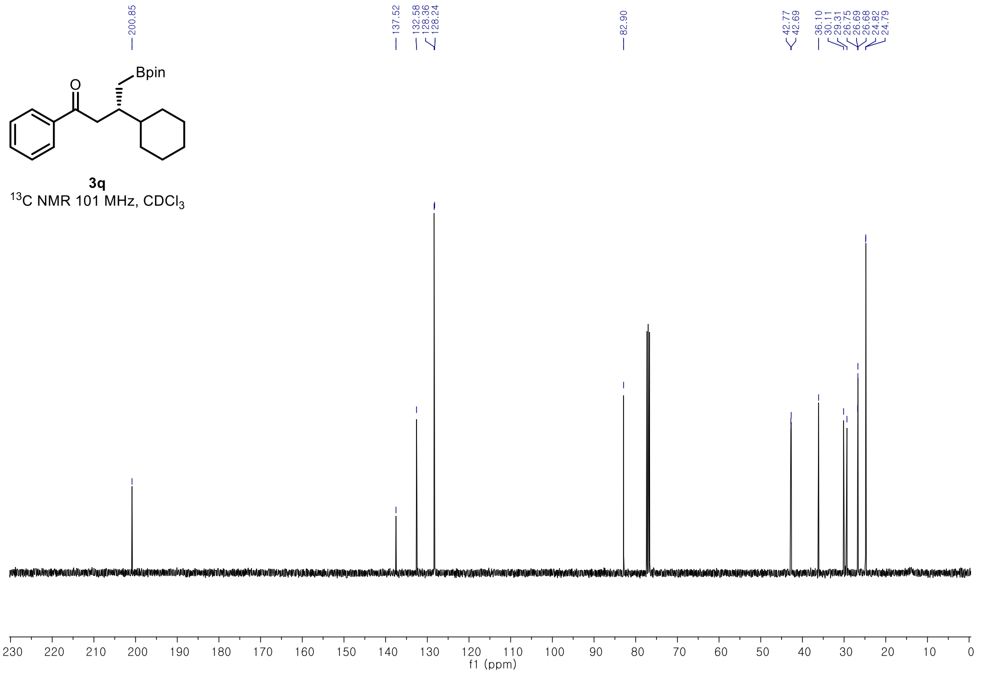

3q
^{13}C NMR 101 MHz, $CDCl_3$

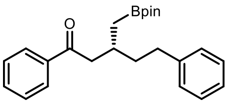

3r
^{1}H NMR 400 MHz, $CDCl_3$

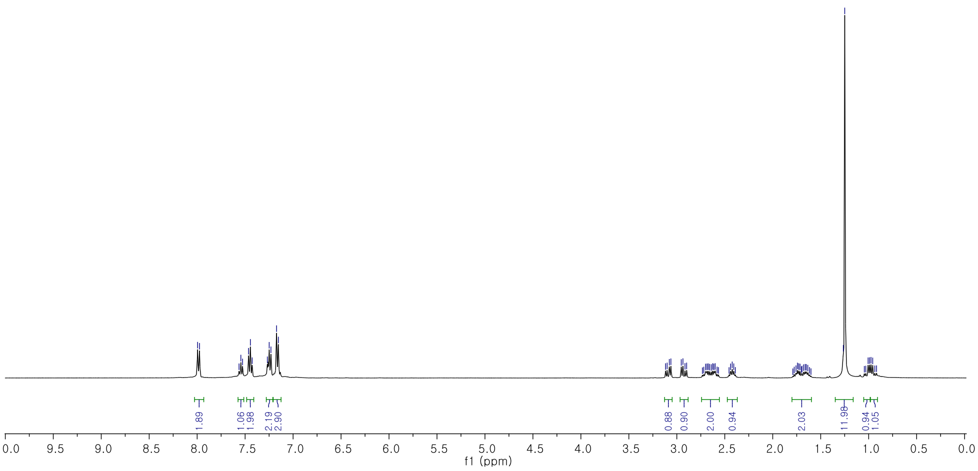

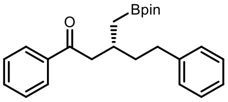

3r
^{13}C NMR 101 MHz, $CDCl_3$

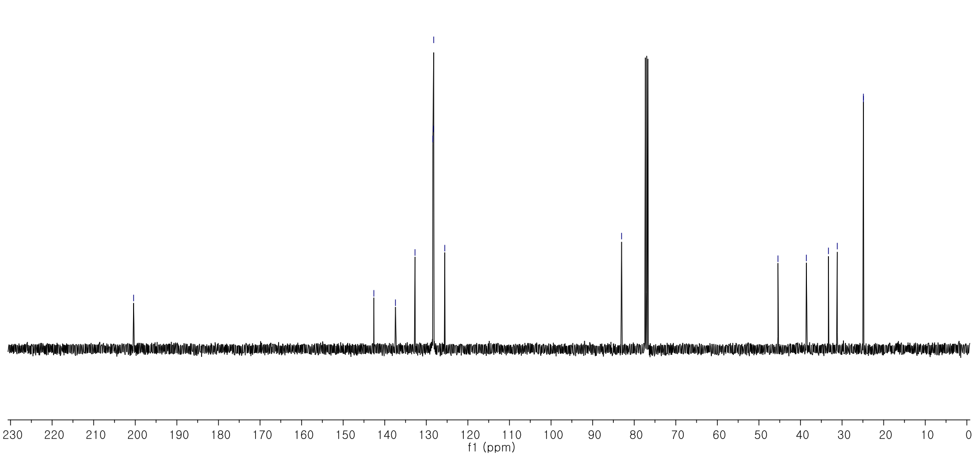

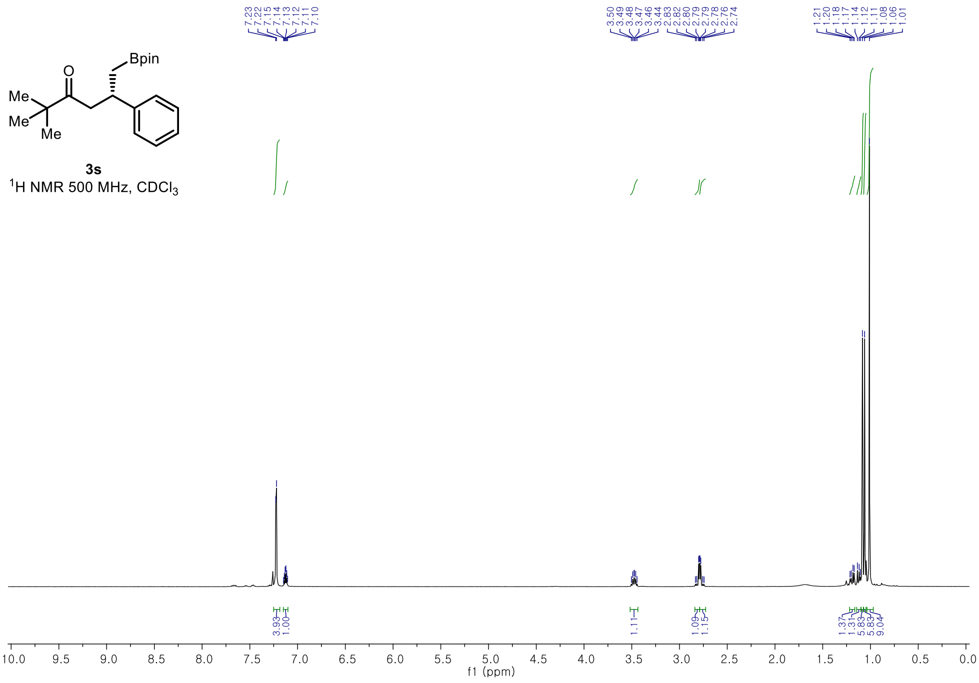
7.23
7.22
7.15
7.14
7.13
7.12
7.11
7.10
3.50
3.49
3.48
3.47
3.46
3.44
2.83
2.82
2.80
2.79
2.79
2.78
2.76
2.74
1.21
1.20
1.18
1.17
1.14
1.12
1.11
1.08
1.06
1.01
Bpin
O
Me
Me
Me
3s
^{1}H NMR 500 MHz, $CDCl_3$
3.93
1.00
1.11
1.09
1.15
1.37
1.31
5.83
5.83
9.04
f1 (ppm)

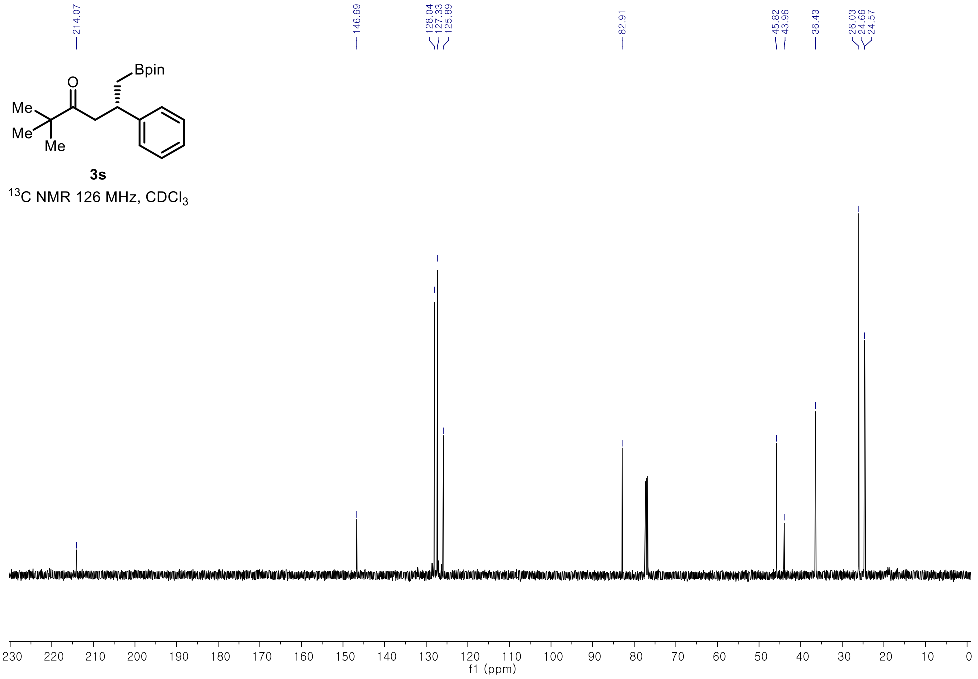
214.07
146.69
128.04
127.33
125.89
82.91
45.82
43.96
36.43
26.03
24.66
24.57
Bpin
O
Me
Me
Me
3s
^{13}C NMR 126 MHz, $CDCl_3$
f1 (ppm)

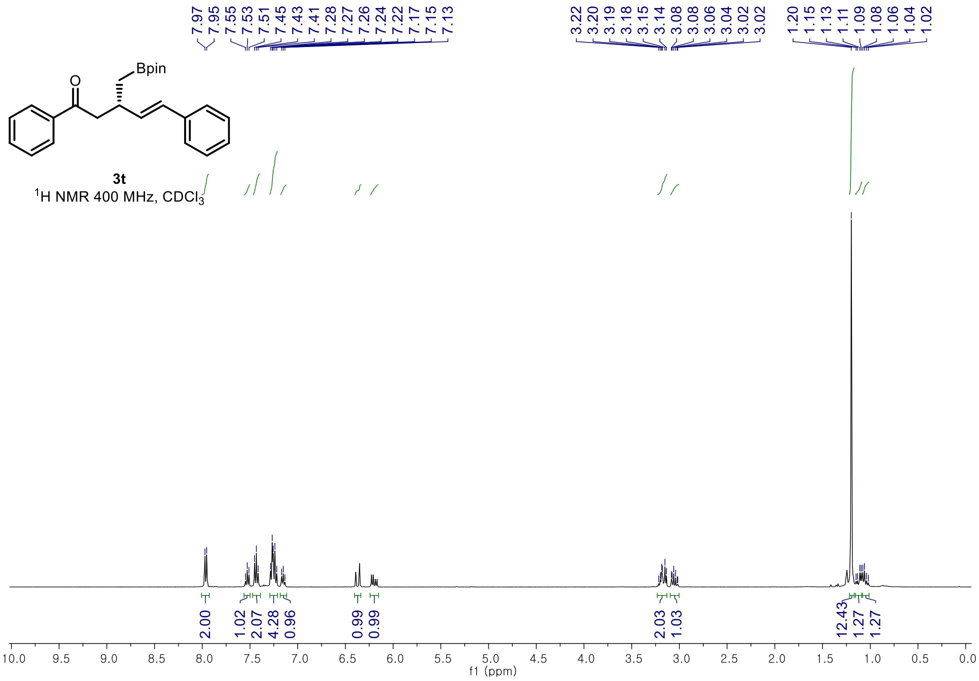
7.97
7.95
7.55
7.53
7.51
7.45
7.43
7.41
7.28
7.27
7.26
7.24
7.22
7.17
7.15
7.13
3.22
3.20
3.19
3.18
3.15
3.14
3.08
3.06
3.04
3.02
3.02
1.20
1.15
1.13
1.11
1.09
1.08
1.06
1.04
1.02
O
Bpin
3t
^{1}H NMR 400 MHz, $CDCl_3$
2.00
1.02
2.07
4.28
0.96
0.99
0.99
2.03
1.03
12.43
1.27
1.27
f1 (ppm)

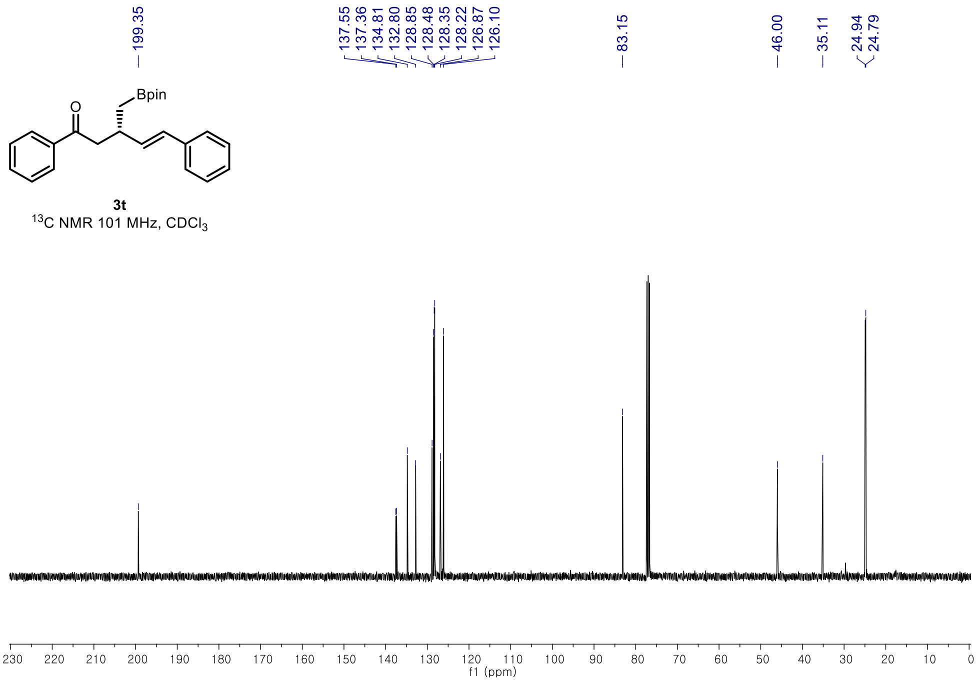
199.35
137.55
137.36
134.81
132.80
128.85
128.48
128.35
128.22
126.87
126.10
83.15
46.00
35.11
24.94
24.79
O
Bpin
3t
^{13}C NMR 101 MHz, $CDCl_3$
f1 (ppm)

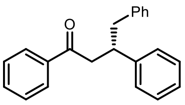

3ab
^{1}H NMR 400 MHz, $CDCl_3$

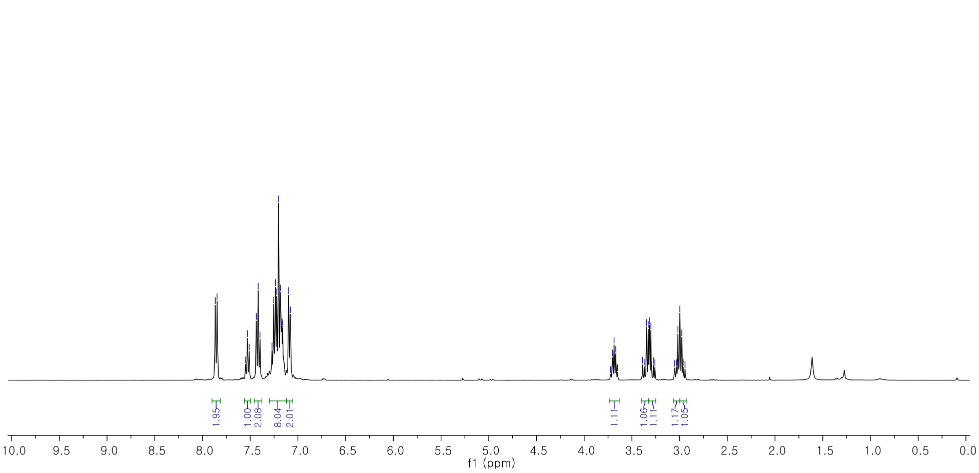

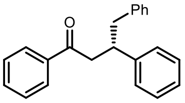

3ab
^{13}C NMR 101 MHz, $CDCl_3$

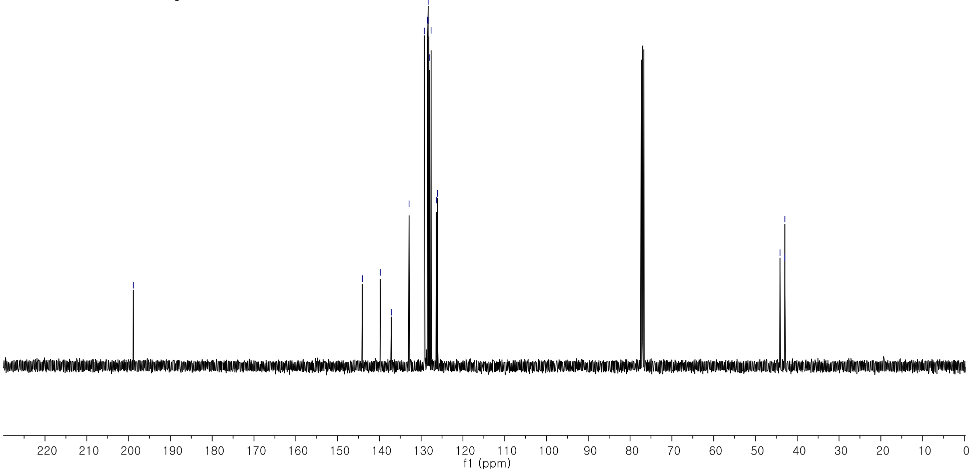

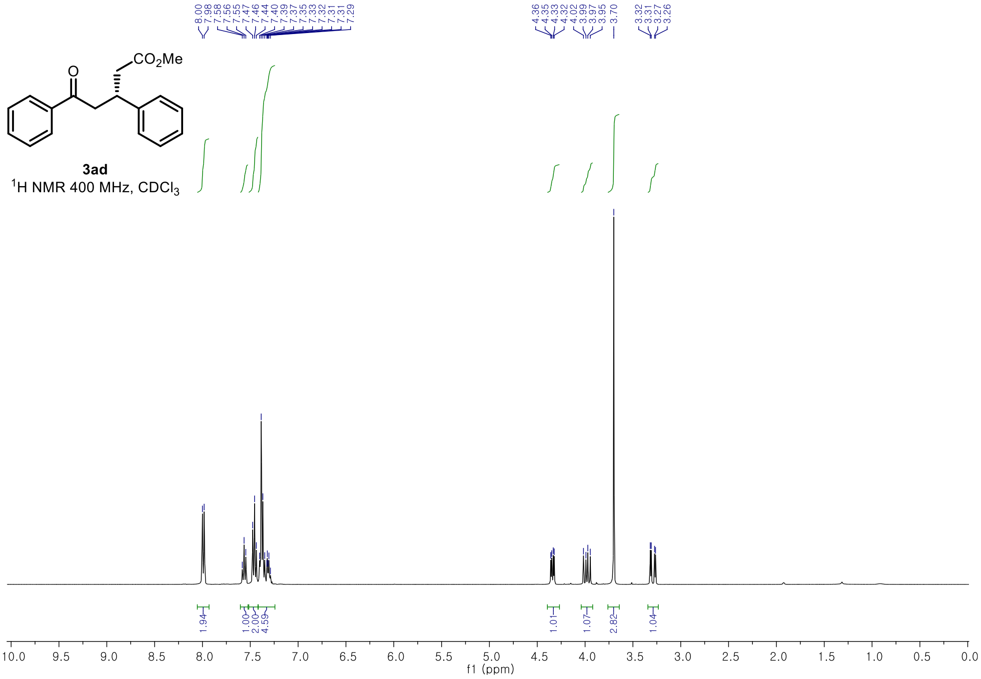

3ad

^1H NMR 400 MHz, $CDCl_3$

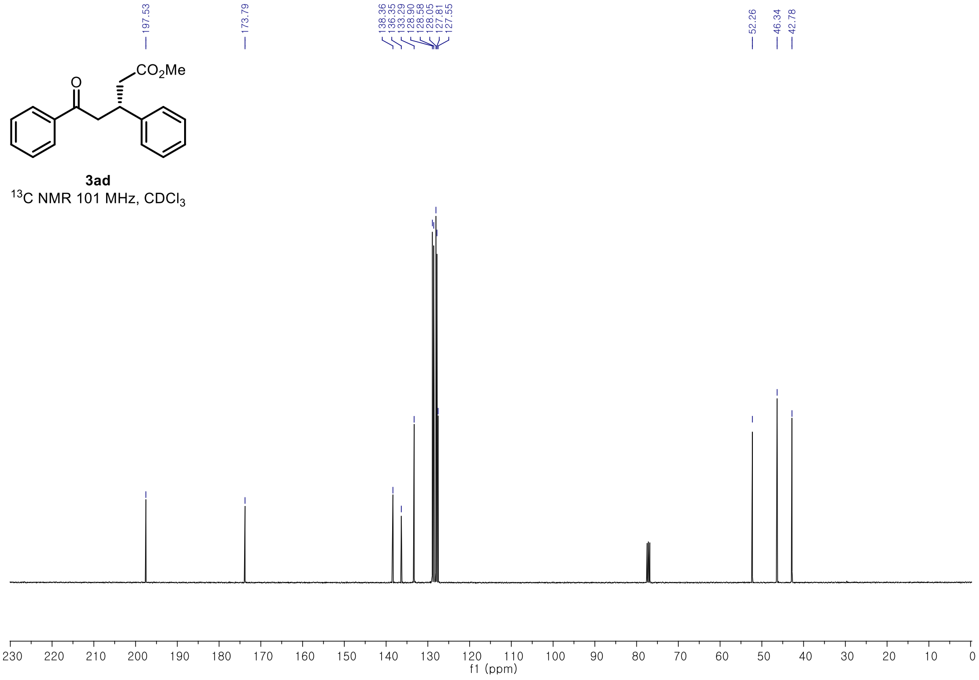

3ad

^{13}C NMR 101 MHz, $CDCl_3$

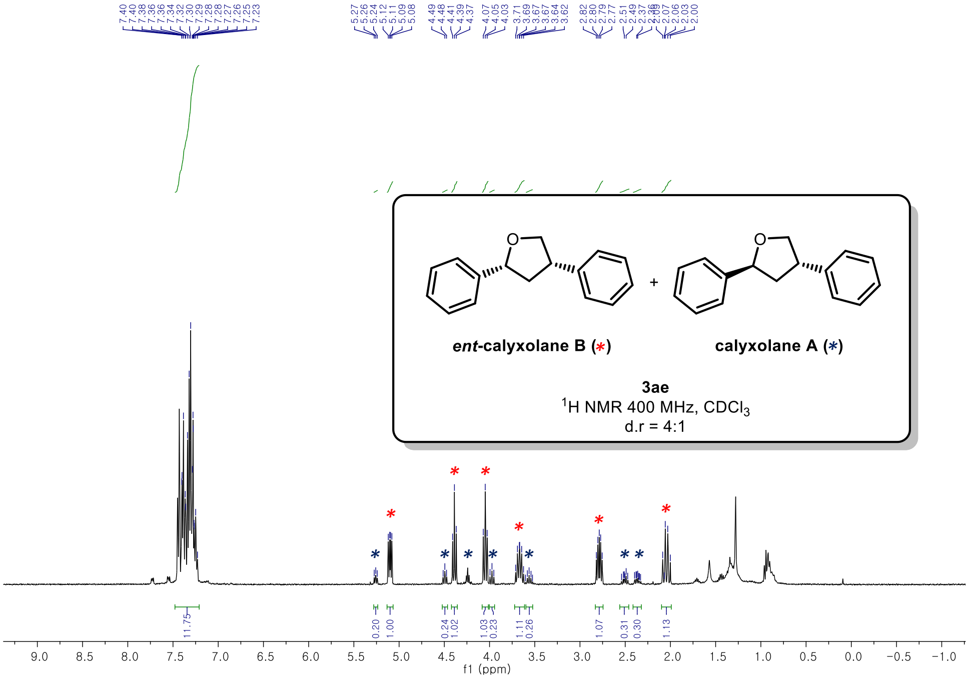

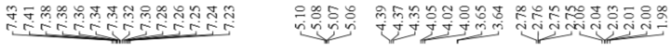

Angew. Chem., Int. Ed., 2017, **56**, 3247.

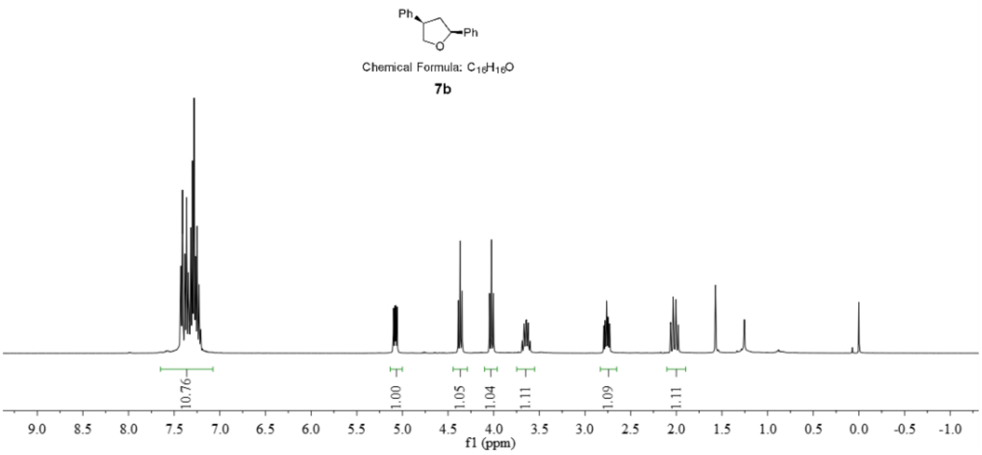

Angew. Chem., Int. Ed., 2017, **56**, 3247.

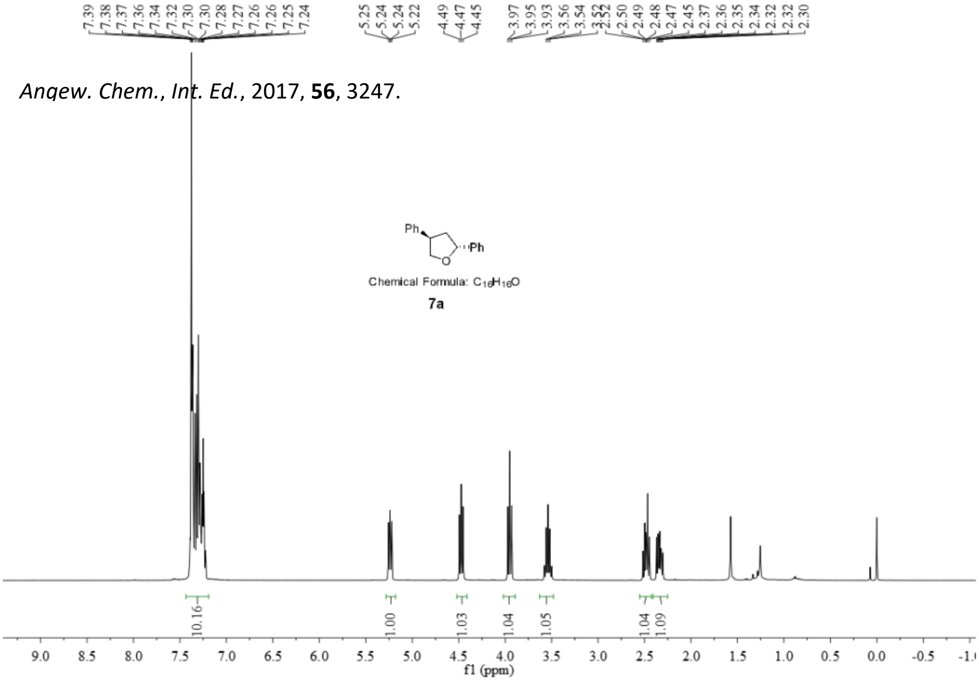

Supplement: SC-012-D0SC06543A-s001 [file SC-012-D0SC06543A-s001.pdf]
